# Venomics Approach Reveals a High Proportion of *Lactrodectus*-Like Toxins in the Venom of the Noble False Widow Spider *Steatoda nobilis*

**DOI:** 10.3390/toxins12060402

**Published:** 2020-06-18

**Authors:** John P. Dunbar, Antoine Fort, Damien Redureau, Ronan Sulpice, Michel M. Dugon, Loïc Quinton

**Affiliations:** 1 Venom Systems & Proteomics Lab, School of Natural Sciences, Ryan Institute, National University of Ireland Galway, H91 TK33 Galway, Ireland; J.DUNBAR2@nuigalway.ie (J.P.D.); michel.dugon@nuigalway.ie (M.M.D.); 2 Plant Systems Biology Lab, Plant and AgriBiosciences Research Centre, School of Natural Sciences, Ryan Institute, National University of Ireland Galway, H91 TK33 Galway, Ireland; antoine.fort@nuigalway.ie (A.F.); ronan.sulpice@nuigalway.ie (R.S.); 3 Mass Spectrometry Laboratory, MolSys RU, University of Liège, 4000 Liège, Belgium; damien.redureau@edu.mnhn.fr

**Keywords:** *Steatoda nobilis*, envenomation, α-latrotoxin, venom, toxins, neurotoxins, necrosis, mass spectrometry, transcriptomics, venomics

## Abstract

The noble false widow spider *Steatoda nobilis* originates from the Macaronesian archipelago and has expanded its range globally. Outside of its natural range, it may have a negative impact on native wildlife, and in temperate regions it lives in synanthropic environments where it frequently encounters humans, subsequently leading to envenomations. *S. nobilis* is the only medically significant spider in Ireland and the UK, and envenomations have resulted in local and systemic neurotoxic symptoms similar to true black widows (genus *Latrodectus*). *S. nobilis* is a sister group to *Latrodectus* which possesses the highly potent neurotoxins called α-latrotoxins that can induce neuromuscular paralysis and is responsible for human fatalities. However, and despite this close relationship, the venom composition of *S. nobilis* has never been investigated. In this context, a combination of transcriptomic and proteomic cutting-edge approaches has been used to deeply characterise *S. nobilis* venom. Mining of transcriptome data for the peptides identified by proteomics revealed 240 annotated sequences, of which 118 are related to toxins, 37 as enzymes, 43 as proteins involved in various biological functions, and 42 proteins without any identified function to date. Among the toxins, the most represented in numbers are α-latrotoxins (61), δ-latroinsectotoxins (44) and latrodectins (6), all of which were first characterised from black widow venoms. Transcriptomics alone provided a similar representation to proteomics, thus demonstrating that our approach is highly sensitive and accurate. More precisely, a relative quantification approach revealed that latrodectins are the most concentrated toxin (28%), followed by α-latrotoxins (11%), δ-latroinsectotoxins (11%) and α-latrocrustotoxins (11%). Approximately two-thirds of the venom is composed of *Latrodectus*-like toxins. Such toxins are highly potent towards the nervous system of vertebrates and likely responsible for the array of symptoms occurring after envenomation by black widows and false widows. Thus, caution should be taken in dismissing *S. nobilis* as harmless. This work paves the way towards a better understanding of the competitiveness of *S. nobilis* and its potential medical importance.

## 1. Introduction

Animal venoms are complex cocktails of toxic proteins that evolved as a primary means to immobilize and subdue prey [1,2] and potentially assist in predigesting the tissues of prey [3,4,5]. However, venoms are also often extremely effective weapons for defence against perceived predators, including humans. Virtually all of the 48,000 species of spiders described so far are venomous [6]. Among these, black widow spiders from the genus *Latrodectus* represent a significant risk to human health due to the synanthropic habits of some species and their highly potent neurotoxic venom [7].

**Figure 1:**
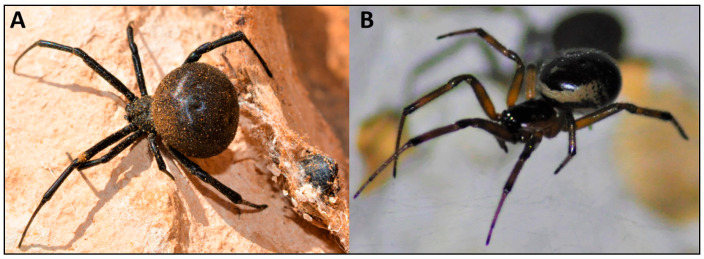
Similarities between black widow and false widow spiders. (**A**) Mature female black widow *Latrodectus lilianae*, Morocco (photo taken by M. Dugon), (**B**) Mature female false widow *Steatoda nobilis*, Ireland (photo taken by J.P. Dunbar).

In recent years, another spider from the widow family (Latrodectinae), the noble false widow *Steatoda nobilis* (Thorell, 1875), which looks superficially like a true black widow (Figure 1) has extended its range globally and may represent a potential risk to native ecosystems and human health [8,9,10,11]. *S. nobilis* is now regarded as potentially being one of the world’s most invasive species of spiders [8]. This species originates from the Macaronesian archipelago [12] and has established populations across Western Europe including Ireland and Great Britain [8,12,13,14,15], through Western Asia (Turkey and Iran) [16,17], and North and South America [10,18,19,20]. *S. nobilis* has an exceptional longevity (up to five years) [21], a fast reproductive rate, is cold tolerant with year-round activity [12], and has a fast-acting venom that allows it to subdue a broad range of invertebrate and even vertebrate prey [12,13]. Outside of its native range, *S. nobilis* has been demonstrated to have a negative impact on native species [8,12,13,22]. In temperate regions, *S. nobilis* has a typical synanthropic lifestyle which brings it in close contact with humans [12]. 

In Europe and South America, *Steatoda nobilis* has been involved in envenomations [9,10,11] commonly resulting in prolonged, moderate to intense pain, swelling and erythema. Other symptoms can include piloerection, diaphoresis, facial flushing, feverishness, vasodilation of the blood capillaries and minor necrosis localised at the bite site [9]. Although the venom of *S. nobilis* has never been investigated before, it has been suggested that symptoms may be triggered by neurotoxins present in their venom [11]. This is because members of the genus *Latrodectus*, the sister genus to *Steatoda*, possess a fast-acting neurotoxic venom (Table 1) that can induce extreme pain and neuromuscular paralysis in humans which can occasionally result in death [23]. The toxicity of *Latrodectus* venom towards vertebrates is mainly due to the presence of α-latrotoxin, a large (130 kDa) neurotoxin which binds to receptors on presynaptic neurones, then forms a pore that allows an influx of Ca^2+^ which triggers an efflux of neurotransmitters [24,25,26]. The α-latrotoxin was first documented in *Latrodectus* and has recently been described in *Steatoda grossa* [7]. It seems likely that it is the presence of α-latrotoxin that allows Latrodectinae spiders (black widows and false widows) to subdue and feed on vertebrate prey [13,27]. 

As this species continues to expand its range, it is inevitable that encounters with humans and subsequent envenomations will increase. Therefore, it has become important to characterise the venom of *Steatoda nobilis* and determine the true medical impact of envenomations. Here, we carried out the first in-depth investigation into the composition of *S. nobilis* venom using a venomics approach, combining venom gland transcriptome and crude venom proteome. The advances of next generation sequencing of mRNA, combined with accurate tandem mass spectra provided by cutting-edge spectrometers, represent the current gold standard method to characterize venom from various species, from small animals such as cone snails [28] to larger ones such as snakes [29]. In recent studies, such approaches led to the unambiguous identification of hundreds of proteins from single specimens. Sticking to these previsions, our results describe with accuracy the molecular composition of the venom of *S. nobilis*, providing a more resolved picture of its potency and a better understanding of its toxic effects. This knowledge is of prime interest to help in the treatment of envenomations, and to understand the competitiveness of *S. nobilis* against native species where it has become invasive. 

## 2. Results

To investigate the diversity and expression of venom proteins in *Steatoda nobilis*, the assembly of the transcriptome obtained from the venom gland mRNAs was used as a database to filter the proteomic results. 

### 2.1. Protein Identification from Transcripts

**Figure 2:**
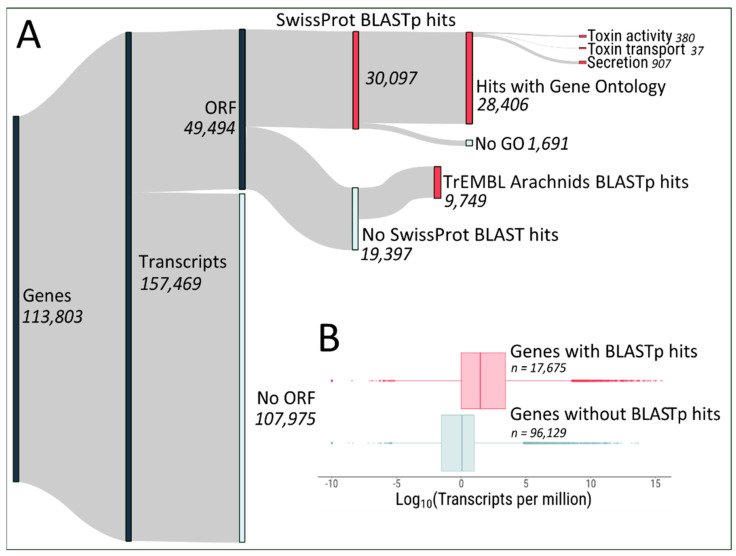
Transcriptomic analysis pipeline of *Steatoda nobilis* venom glands. (**A**) Trinity assembly and annotation metrics. Nodes represent features and their associated numbers found in the assembly. (**B**) Expression level of predicted protein-coding genes versus non-coding genes.

The transcriptome assembly yielded 113,803 genes and 157,469 transcripts (Figure 2A). Out of those, 49,494 contain Open Reading Frames (ORFs) encoding proteins of >75 amino acids in length. Homologies between the predicted proteins and proteins found in the Uniprot database found 30,097 proteins with homology against the high-quality manually curated SwissProt database. Out of the remaining 19,397 predicted proteins, 9749 had matches against the computationally analysed TrEMBL database (restricted to Arachnids). The set of ~40,000 transcripts encoding proteins with BLASTp hits against either database likely represent the biologically relevant transcriptome of *Steatoda nobilis* venom glands, and is encoded by 17,675 genes. Indeed, the expression level of genes coding for predicted proteins with BLASTp hits is generally higher than the expression level of genes without ORFs/BLASTp hits (Figure 2B). 

**Figure 3:**
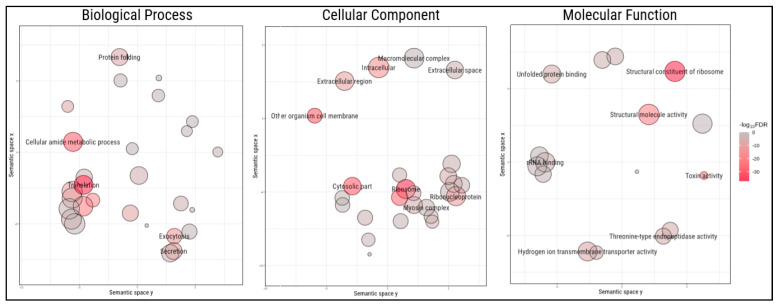
Enriched gene ontologies among highly expressed genes in *Steatoda nobilis* venom glands. Circles represent significant gene ontologies, arranged according to their semantic space. The size of the circles represents the number of genes associated to the given ontology in the dataset, while the colours represent the −log_10_ false discovery rate for the ontologies’ enrichment.

Among the protein-coding genes, we found 380 genes associated with toxin activity, 37 genes with toxin transport and 907 genes associated with secretion, indicating that our assembly likely represents a good overview of the venom gland transcriptome of *Steatoda nobilis*. Indeed, gene ontology analysis of the most expressed genes (arbitrarily chosen as > 100 transcripts per million (TPM), 872 genes) compared with the entire set of protein-coding genes with gene ontologies (28,406 genes) showed enrichments for toxin activity, exocytosis, myosin filament, toxin transport and peptidase activity (false discovery rate (FDR) < 0.05), (Figure 3). The full list of GO enrichment can be found in Dataset S1. 

**Figure 4:**
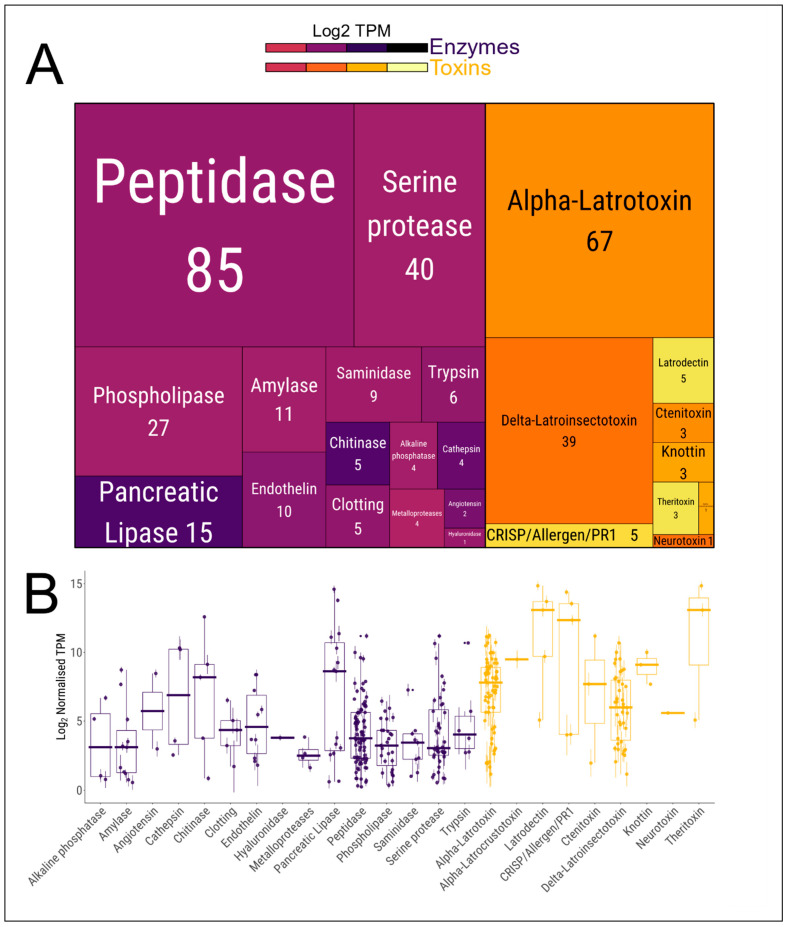
Relative abundance and expression level of genes encoding predicted venom enzymes and toxins. (**A**) Treemap chart of the main classes of venom-related enzymes and toxins present in *Steatoda nobilis* venom gland transcriptome. The size of the rectangles is proportional to the number of genes in each category, indicated under the labels. The colour represents the log_2_ transcripts per millions (TPM) of the median expression of the genes in each category. (**B**) Expression levels of each gene in each enzyme/toxin category. Dots represent the mean expression of individual genes across three biological replicates ± SD; purple: enzymes, orange: toxins.

Potential genes encoding venom proteins were identified based on homology with known toxin/enzymes identified in previous venom studies. The list of putative venom-encoding genes was further reduced by retaining only those genes with an expression level > 1 TPM across three biological replicates. We found 228 and 127 genes encoding for enzymes and toxins, respectively (Figure 4A). Among the enzymes, peptidases and serine proteases are the most abundant (85 and 40 genes, respectively), with the most expressed classes of enzymes being pancreatic lipases and chitinases (Figure 4B). The toxin genes are mainly comprised of α-latrotoxin and δ-latroinsectotoxins (67 and 39, respectively), (Figure 4A), with a generally higher expression for latrodectin, CRISP/Allergen/PR1 and theritoxin genes (Figure 4B). Altogether, the transcriptomic data indicates a diverse arsenal of venom enzymes and toxins produced by the venom gland of *Steatoda nobilis*.

### 2.2. Shotgun Proteomics of Steatoda nobilis

Crude venom extracted from a female *Steatoda nobilis* was reduced, alkylated and digested with trypsin before being separated using micro-HPLC hyphenated to Q-Exactive, leading to the acquisition of 10,225 MS and 34,343 MS/MS scans. High-resolution mass spectrometers, such as Orbitraps ensure both high efficiency in fragmentation and accurate mass measurements of parent and fragment ions. In these conditions, *de novo* sequencing of peptides, performed with Peaks X+, becomes more powerful and accurate. Imposing a precursor mass tolerance < 5 ppm and fragment ions mass tolerance < 0.015 Da, 7759 peptides were sequenced with a *de novo* score >50, of which 2974 (38.3%) displayed a score above 80. *De novo* score is a criterion of quality, expressed in percentages, linked to residue local confidence (the higher the score, the higher the confidence in the sequencing). The sequence tags contain from 6 to 23 amino-acids, and have masses ranging from 799.4 to 2524.24 Da. 

#### Qualitative Data Analysis

To evaluate the quality of the proteomics data, the sequencing was compared to two databases of protein sequences, extracted from Uniprot (3 March 2020, https://www.uniprot.org/). The first database was obtained by collecting all the protein sequences returned using the keyword “Spider” (n = 317,450 sequences). The second database contained 2778 sequences selected from Uniprot using the keywords “Spider AND Toxins”. For each analysis, a false discovery rate of 0.1% was applied, to only keep the best matches. With these parameters, the database « Spider » identified 53 proteins, with the help of 773 peptides from the 7759 *de novo* sequences (10.0%). Of those proteins, 23 were identified as various hemocyanins (43%). The presence of these proteins may result from the venom extraction process, when haemolymph is accidently drawn into the venom duct and excreted with the venom. Hemocyanins participate in the formation of the arthropod cuticles and in the wound healing process. The best identification however is a δ-latroinsectotoxin-Lt1a (AN = Q25338) from *Latrodectus tredecimguttatus*, the Mediterranean black widow. The toxin family is unambiguously identified with 16 unique peptides describing 10% of the whole protein sequence (−10lgP = 208.57). U11-Theriditoxin-Lha1d, from the Australian black widow *Latrodectus hasselti* is also identified within the best matches (4 unique peptides, 26% of sequence coverage, −10lgP = 112.71, AN = A0A482ZCV4). These two toxins clearly confirm *Steatoda nobilis* as a close relative to the species of the *Latrodectus* genus. The database “Spider+Toxin” aimed at focusing the search on spider toxins, excluding additional proteins such as hemocyanins. Using the same parameters, only six proteins were identified from 476 peptides (6.1%). These proteins are two isoforms of δ-latroinsectotoxin-Lt1a, an α-latrocrustotoxin-Lt1a and an α-latrotoxin-Lt1a from *Latrodectus tredecimguttatus,* an α-latrotoxin-Lh1a from *Latrodectus hasselti* and finally a toxin from *Cupennius salei* named Toxin 21. Even if *Ladrodectus* toxins are mainly identified, the results of this qualitative analysis collected with the help of the Uniprot database are relatively poor. This clearly highlights the need to combine proteomics and transcriptomic data for unsequenced organisms, and in our case, to gain a better overview of the venom of *Steatoda nobilis*. 

### 2.3. Integration of Transcriptomics and Proteomics Data 

#### 2.3.1. Qualitative Data Analysis 

**Figure 5:**
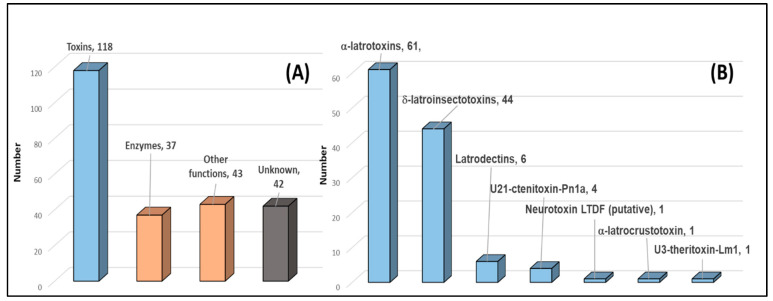
(**A**) Distribution of the 240 proteins identified from *S. nobilis* venom gland transcriptome and proteome, classified into four major protein groups showing the predominant presence of toxins and enzymes in the venom. (**B**) Distribution of the 118 toxin sequences grouped into seven different families, where α-latrotoxin, δ-latroinsectotoxins and latrodectins combined make up 94% of the identified toxins and more than 46% of the 240 identified protein sequences.

Transcriptomic data were used as a sequence database for analysing proteomics *de novo* sequences. Using the same search parameters, 240 proteins were identified with significant peptides, from 4161 of the 7759 *de novo* sequenced peptides (53.6%). This first result was already very encouraging as many more proteins were identified from both approaches, validating the quality of not only the transcriptomic data but also the proteomics-based sequencing. A BLAST of each of the 240 sequences identified 118 of these as toxins (49.2%), 37 as enzymes (15.4%), 43 as proteins with other functions (17.9%), such as cysteine-rich secretary proteins (CRISPs, ×8), hemocyanines (×5) or histones (×2). There were also 42 identified from mRNA and from the venom, but those proteins present unknown biological activities (17.5%) (Figure 5A). The 118 toxin-annotated sequences were classified into 9 different families (Figure 5B). Among these, a large number of α-latrotoxins, δ-latroinsectotoxins and latrodectins, commonly expressed in the venom of *Latrodectus* species, were again identified. Importantly, the 118 identified toxin proteins using proteomic data to mine transcript data closely matched the 127 predicted toxin transcripts from the venom gland transcriptome (identified against SwissProt and TrEMBL databases), highlighting the power of the approach. Altogether they represent 94% of the identified sequences; other toxins are a minority in the venom.

**Figure 6:**
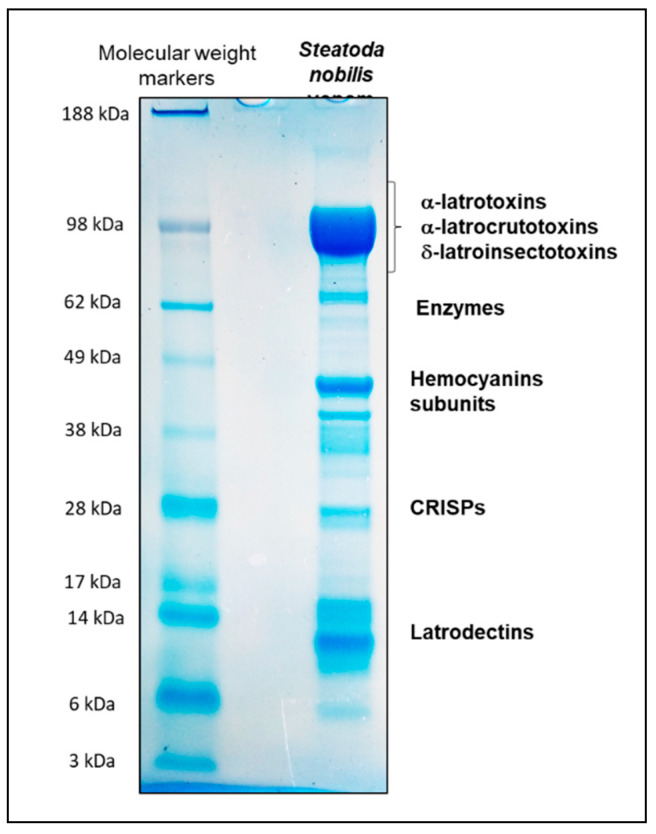
1D SDS-PAGE analysis of *Steatoda nobilis* crude venom showing the most abundant protein families of this venom.

This data suggests that the venom of *Steatoda nobilis* contains plenty of highly bioactive toxins. However, it is difficult to fully appreciate these results without quantifying each kind of toxin. A 1D SDS-PAGE analysis of crude *S. nobilis* venom provided a rough idea of the abundance of each family of proteins (Figure 6). The electrophoretic separation of the crude venom led to a large number of intense bands which were interpreted based on their molecular weights. The most intense band (90 and 140 kDa) corresponded to the presence of α-latrotoxin, δ-latroinsectotoxins and α-latrocrustotoxin, which have molecular masses in this range. The intensity of the bands indicates that these toxins constitute the most concentrated group of proteins in the venom. Two others intense bands were detected at 40 kDa and 10 kDa respectively, associated with hemocyanins and latrodectins. Other proteins such as CRISPs were also potentially among the most abundant compounds present in the venom. Although all of these observations were consistent with our previous results, the 1D SDS-PAGE gel did not provide any information on the relative abundance of each kind of protein family present in a single band (for example, the band around 100 kDa). In this context, a relative quantitative approach is necessary to get a better overview of the molecular composition of this venom. 

#### 2.3.2. Relative Quantitative Analysis

**Figure 7:**
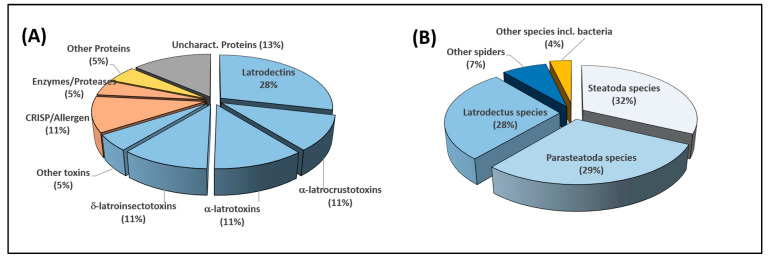
(**A**) Pie-chart showing the relative abundance of the 199 proteins quantified in the venom of *Steatoda nobilis*, arranged into families. The blue slices detail the toxin content of the venom. The orange slices are non-toxin proteins that are important to venom activity. The yellow slice represents the proteins not directly related to venom (e.g., hemocyanins). The grey slice comprises all the proteins with undetermined biological activity. (**B**) Pie-chart showing the relative abundance of the 199 quantified proteins, arranged by genera with matching sequences (determined by BLAST).

Absolute protein quantification by mass spectrometry is usually done by adding isotopically labelled internal standards to the sample. However, here it would have been very impractical to use this method to consider each of the 240 proteins identified in the venom of *Steatoda nobilis*. Instead, we used relative quantification of venom proteins. Quantifying the proteins based on the signal intensities of their three most intense ions provides a good evaluation of protein abundances, even in complex mixtures [30]. This approach has demonstrated its usefulness to quantify totally different classes of proteins, with low sequence identity and without common peptides. Unfortunately, in the case of venoms, this way of quantification can create a bias due to the presence of many isoforms. For example, each of the α-latrotoxins sharing a high sequence identity with others would be quantified according to the three most intense ions of the α-latrotoxin population. In other words, if one α-latrotoxin isoform is very abundant in the venom, all the isoforms, even those with low concentration would be quantified at the same quantity because they share the same three most intense ions. To avoid this issue, here the relative quantification has been expressed from the three most intense signals coming from unique peptides for each toxin. A “unique peptide” is a peptide that does not share its sequence with any other toxin identified in the experiment. The use of unique peptides greatly improves the selectivity of the quantification. The drawback of this approach is the loss of some identifications based on relevant peptides shared between sequences. Here, only 199 out of the 240 identified proteins displayed at least one unique peptide. As a result, only those were considered for relative quantification. It is however an acceptable compromise regarding the complexity of the venom. Out of these 199 proteins, 118 possess at least three unique peptides, 37 only two and 44 one unique peptide. To normalize the data, the average intensity was set to quantify the 155 proteins possessing two or three unique peptides. Figure 7A presents the quantified proteins expressed in percentages and classified according to their families. Even if the quantification is relative and must be interpreted with care, the figure clearly shows that two thirds of the venom is composed of toxins (66% of the total intensity of the signal). Interestingly, the most concentrated toxins in the venom of *Steatoda nobilis* are *Latrodectus*-like toxins. This finding is in good agreement with the transcriptomic data, suggesting that venom composition is largely controlled at gene expression level. Figure 5B displays the genera from which the 199 proteins are the closest. While the genera *Steatoda* and *Parasteatoda* are the two closest (32% and 29%, in relative signal intensity), the genus *Latrodectus* represents more than a quarter of the whole venom, thus confirming a close relationship between the “false” and the “true” widow spiders. 

The three most abundant individual toxins represent 43.5% of the overall quantified venom and the twelve most abundant account for more than 70% of the quantified content (Table 2). The main toxin families are latrodectins, α-latrocrustotoxins, CRISPs, α-latrotoxins and δ-latroinsectotoxins. The most abundant single toxin, latrodectin-Sno1a, accounts for 23.5% of the quantified toxins. Also, two of the most important compounds have uncharacterized activities. If these compounds are secreted in such an important proportion, their function, which has yet to be determined, is probably essential to the potency of the spider’s venom. 

Alignments of the main sequences found in this study with some already known toxins are provided as Dataset S2. 

## 3. Discussion

We investigated the venom composition of the noble false widow spider *Steatoda nobilis* using transcriptomics and proteomics. The ability of venomous animals to target various pathways of multiple prey types is facilitated by a diverse toxin repertoire [1,2]. As seen with *Latrodectus* species, *S. nobilis* is also capable of subduing a diverse range of invertebrates but also vertebrate prey [12,13]. The ability for *Latrodectus* to subdue vertebrates is due to α-latrotoxin which subsequently can be highly potent to humans [7]. Advanced venomic techniques combining transcript libraries produced from next generation sequencing of venom-gland RNA with tandem mass spectrometry of crude venom allowed us to identify 240 molecules representing four protein groups, of which 49% are toxins, 15% are enzymes, 18% are proteins with other functions and 18% are proteins with unknown biological functions. Comparison of the transcriptome of *S. nobilis* (presented here) with the transcriptome/proteome of other spiders using spider-specific tools such as ArachnoServer [31] will represent an important follow-up study to compare the evolution of spider venom systems. We confirm for the first time the presence in the venom of *S. nobilis* of various toxins already described from *Latrodectus* species, such as α-latrotoxin which is among the most dominantly expressed toxins. Unsurprisingly, and in addition to arthropod-specific neurotoxins such as latrodectins, α-latrocrustotoxin and δ-latroinsectotoxins, these *Latrodectus*-like toxins made up over two thirds of overall toxin composition. 

### 3.1. Toxins

From a biological point of view, when α-latrotoxin binds to receptors such as neurexins and latrophilins on presynaptic neurones, it permeates the membrane by penetrating the lipid bilayer and forming a pore that allows an influx of Ca^2+^ which triggers an important efflux of neurotransmitters. Once the vesicles are depleted, the signals between nerve and muscles are blocked, leading to neuromuscular paralysis [25,32]. Latrodectins also known as α-latrotoxin associated LMWPs are suspected of enhancing the potency of latrotoxins by altering the ion balance near different channel types, thus regulating Ca^2 +^ influx and neurotransmitter release. However, they are not known to be toxic to insects or mammals in their purified form [33]. Other proteins detected in the venom that target neurones include CRISP/Allergen/PR-1 which block Ca^2+^ channels [34], U21-ctenitoxin-Pn1a which has a protease activity through the hydrolysis of peptide bonds [35], as well as a putative neurotoxin LTDF 06-01, and U3-theritoxin-Lm1 which have a neurotoxin-like activity. Collectively, these toxins act simultaneously to facilitate targeting and disrupting various aspects of normal nerve function. *Steatoda nobilis* is a very generalist predator. In addition to producing strong three-dimensional cobwebs [13], they also use a very effective “attack wrap” strategy to immobilize would-be prey or predators alike [36]. Consequently, as an opportunist, they are often faced with tackling invertebrate and/or vertebrate prey that can be strong, fast, aggressive, and many times larger than them. Therefore, the most effective way to immobilize captured prey safely and efficiently is by inducing rapid paralysis. We previously observed wild caught specimens of *S. nobilis* biting insects and spiders and causing a rapid reduction in motor function (unpublished data). The mechanical bite from an adult *S. nobilis* is almost painless, however, the rapid release of neurotransmitters induces intense pain and is therefore also an effective weapon for defence. Victims of *Latrodectus* and *Steatoda* bites typically experience immediate sharp pain, which are attributed to the effects of α-latrotoxins [24].

While necrosis has not been reported as a symptom of envenomation by *Latrodectus* species [6,37], in high doses (>10 nM), α-latrotoxin can reduce ATP levels in the nerve terminal, compromising plasma membrane integrity and releasing cytoplasmic markers, including glutamate, y-aminobutyric acid, aspartate and α-aminoisobutyrate. This can result in morphological alterations, swelling of mitochondria and subsequent cell death [26,32,38]. As such, α-latrotoxin may indirectly result in cell death. In *Latrodectus hesperus*, transcriptomic studies showed that 39 latrotoxin sequences account for 16% of venom gland expression [24], whereas our study showed that 61 α-latrotoxin sequences, accounting for 52% of the identified toxins, were detected in *Steatoda nobilis* venom. This higher concentration of α-latrotoxins in *S. nobilis* venom might explain the minor necrosis localised around the bite site previously described in the literature [9]. The high concentration of α-latrotoxin in *S. nobilis* venom is significant as it most likely plays a primary role in prey immobilisation, defence and subsequent medical envenomations. The venom of *Latrodectus* can induce intense pain, diaphoresis, paresthesia, hypertension, fasciculations of muscles and ultimately neuromuscular paralysis occasionally leading to death [24]. In the first case of envenomation by *S. nobilis* [11], some neurotoxic symptoms were reported as systemic. While severe neurotoxic symptoms have not yet been reported from *S. nobilis*, increasing reports of envenomation by the latter should be a concern, especially given their close relatedness to *Latrodectus*. In the overwhelming majority of envenomations by *Latrodectus*, the victims do not require medical attention; only a small percentage result in severe symptoms and death. Only seven medically assessed cases of *S. nobilis* envenomations have been reported so far. As this species is becoming more widespread, envenomation occurrences are likely to rise, and reports of severe envenomations, although rare, should be expected. Therefore, we recommend caution on dismissing these spiders as harmless until larger scale reporting of case studies is compiled, and clinical assays are carried out to further verify the medical potential this highly neurotoxic venom has on human health. 

### 3.2. Enzymes

A range of enzymes making up 15% of *Steatoda nobilis* venom suggests that the venom not only plays a role in prey immobilization, but also assists in the predigestion of the prey’s tissue. These include a pancreatic lipase-related protein which hydrolyses both phospholipids and galactolipids. Chitinase, also represented, is involved in the breakdown of the exoskeletons of arthropods [24], Carboxypeptidase is thought to remove the C-terminal Arg-residues from immature venom peptides [6]. Astacin-like metalloprotease are proteases that function in the metabolism of extracellular matrix components. The astacins are proteases that may aid as a spreading factor for other venom toxins [39]. Proclotting enzyme is most likely involved in promoting coagulation of haemolymph in prey, which may aid as an immobilizing toxin. Cathepsin has protease activity and an endothelin-converting enzyme homologue degrades large endothelins into smaller forms, which display vasoconstriction activity. Pancreatic α-amylase is involved in endohydrolysis of (1->4)- α-D-glucosidic linkages in polysaccharides, such as cellulose and chitin, suggesting a role in the breakdown of cytoskeleton. The β-hexosaminidase subunit β is involved in the degradation of gangliosides on the cellular surfaces of neuronal cells, this may indicate a toxin function for immobilization. Angiotensin-converting enzymes interact with metabolic pathways causing disturbances of the cellular homeostasis and thus contributing to prey immobilization [6]. Alkaline phosphatase has a proteolytic activity, peptidylglycine α-hydroxylating monooxygenase-like is involved in electron transport and anionic trypsin-2-like cleaves peptide bonds in proteins. Altogether, this array of enzymatic proteins suggests that, like other venomous animals such as snakes [3], the venom of *S. nobilis* does likely play a role in the predigestion of prey [4,5] and may possibly contribute towards cell death at the bite site.

## 4. Conclusions

This study proposes for the first time an in-depth investigation of the venom of a *Steatoda* species, a close relative of *Latrodectus*. We reveal the striking similarity between the toxins found in *Steatoda nobilis* venom and that of black widow spiders (Table 1). The most powerful toxin classes (α-LTX, α-LCT, α,δ-LIT) and the enzymatic machinery allowing the venom to more easily spread into the prey (metalo and serine proteases, chitinases) are both present in large quantities. This however does not mean that *Steatoda* is as dangerous to human beings as some members of the genus *Latrodectus*. If isoforms of potent toxins are present, our study does not provide information about their potency. Evaluation of toxin toxicity would need to be performed before any conclusion could be reached. Nevertheless, given the composition of the venom depicted in this study, *S. nobilis* should be considered a species of medical importance and there is no doubt that *S nobilis* (with *Latrodectus tredecimguttatus)* is one of the most dangerous spiders in Western Europe. Moreover, as *S. nobilis* continues to expand its range, its impact on native wildlife needs to be monitored and its potential invasiveness assessed. In temperate regions where it occupies synanthropic habitats, envenomations of medical importance will undoubtedly rise as the species becomes more prevalent in and around human habitations. The work carried out in this study will hopefully help researchers across disciplines to better understand the evolution of the Latrodectinae family, the competitiveness of *S. nobilis*, and the potential medical importance of envenomations.

## 5. Materials and Methods 

### 5.1. Spider Collection and Venom Extraction

All specimens of *Steatoda nobilis* used in this study were collected in the Republic of Ireland, from street furniture, garden walls and park railings in the general area of Lucan, Co. Dublin. In total, 80 specimens were collected and identified as female from the presence of the epigyne. The spiders were anesthetized using CO_2_ for 2 min and venom was extracted by electrostimulation with repeated pulses delivered at 15–20 V. Venom droplets were collected from the venom pores located on the outer subterminal part of the fangs using 5 µL microcapillary tubes modified with a tapered end for maximum efficiency. The venom was pooled, then flash-frozen in liquid nitrogen, lyophilized and stored at −20 °C.

### 5.2. Venom Gland Removal and RNA Extraction

Adult *Steatoda nobilis* specimens were collected from the same location as above. Venom extraction was carried out on 25 females, and three days later the spiders were euthanised with an overdose of CO_2_ and once dispatched, using micro-dissection tweezers the dorsal exoskeleton was removed exposing the venom glands. The glands were removed and pooled together in a 2 mL tube containing RNA later (Ambion), with three biological replicates. Venom glands were then flash-frozen in liquid nitrogen and ground to a fine powder using a bead mill (Qiagen Tissue Lyser II). Immediately after grinding, 0.5 mL of TRIzol was added before samples thawed. Samples were shaken for 15 s, incubated at room temperature for 5 min and then centrifuged (25,000× *g*) at 4 °C for 10 min. The supernatant was collected and transferred into new tubes. Nucleic acids were separated by the addition of 100 µL of chloroform, followed by 30 s of shaking, and 3 min incubation at room temperature. Then, the two phases were separated by centrifugation for 10 min at 4 °C. Nucleic acid precipitation was performed on 200 µL of the upper aqueous phase with 200 µL of isopropanol, mixed for 10 s, incubated in ice for 15 min and centrifuged for 15 min. The pellets were washed twice with 75% EtOH (prepared in DEPC water). Finally, the pellets were air dried at 37 °C and resuspended in 50 µL of RNAse-free water. Following extraction, DNA was removed from the nucleic acids using DNAse 1 (Sigma-aldrich AMPD1), following manufacturer’s instructions. 

Finally, DNA-free total RNAs were cleaned up and concentrated using a silica column-based kit (Zymo Research RNA Clean & Concentrator). 

### 5.3. Transcriptomics Assembly and Analysis

For the preparation of RNA transcript libraries and sequencing, samples were sent to Novogene Company Limited, Cambridge Science Park, Milton Road, Cambridge, CB4 0FW, UK. Libraries were generated using mRNA enrichment, and sequencing was performed using Illumina technology (150 bp paired-end reads). For all three libraries, >50 million 150 bp paired-end reads were obtained.

Raw reads were corrected using Rcorrector [40] and leftover Illumina adapters removed using trimmomatic [41]. Then, reads mapping to ribosomal RNAs (using bowtie2 [42] mapping against *Latrodectus* and *Steatoda* ribosomal RNAs sequences available in NCBI) were removed to ensure good representation of reads belonging to mRNA transcripts in the transcriptome assembly. The transcriptome of one of the three venom gland samples was assembled using the Trinity pipeline [43,44]. A single sample was used for assembly to limit the hardware requirements of such a method. Transdecoder [45] was used to identify Open Reading Frames (ORFs) originating from the transcripts, with a minimum protein length of > 75 amino acids. We intentionally lowered the minimum ORF prediction from Transdecoder (100) to 75 amino acids in length, to account for the presence of potentially relevant small proteins, such as those described in [46]. The assembly was annotated using Trinotate [47], based on ORFs homology using BLASTp (e-value cut-off of 1 × 10^−3^) against (i) SwissProt curated database [48] and (ii) TrEMBL Arachnids database (also [48]). In addition, ORFs were compared to the Protein family (Pfam) database [49]. A summary of the annotation results is shown in Figure 2.

Gene expression analysis was performed as part of the Trinity pipeline using Kallisto [50] on the three venom gland libraries, using the assembled transcriptome generated by Trinity. The gene expression matrix obtained was used to extract genes with high expression level (>100 transcripts per million (TPM)) for Gene Ontology (GO) analysis. GO terms associated with the annotated genes were retrieved using Trinotate’s extract_GO_assignments_from_Trinotate_xls.pl script. GO analysis was performed using GOseq [51], with the genes > 100 TPM as input (~900 genes), against the background of all ~17,000 genes with GO annotations. Over-representation of Gene Ontologies was analysed using ReviGO [52], and plotted in R [53]. For venom-encoding genes, a relative abundance of enzyme and toxin-producing genes was generated using the Treemap package in R [54].

### 5.4. SDS-PAGE of Steatoda nobilis Female Venom

The 10 µg sample of *S. nobilis* venom was diluted in Laemlu buffer, heated for 3 min to 100°C, before being separated using 1D SDS-PAGE NuPage (ThermoFisher Scientific) in MES SDS buffer. A 6 µg sample of a standard composed of insulin beta-chain (3 kDa), aprotinine (6 kDa), lysozyme (14 kDa), red myoglobin (17 kDa), carbonic anhydrase (28 kDa), alcohol dehydrogenase (38 kDa), glutamic dehydrogenase (49 kDa), bovine serum albumin (62 kDa), phosphorylase (98 kDa) and myosin (188 kDa), was used as a molecular weight marker. The electrophoresis was performed by applying 200 V during 40 min to the system. The resulting gel was firstly dehydrated with 50% EtOH and phosphoric acid 3% during 3 h, then rehydrated by means of a 20 min bath of ultrapure water (MilliQ). The colouration of the proteins was performed overnight with Coomassie blue (360 g/L, in an aqueous buffer with 34% MeOH, 3% phosphoric acid and 17% ammonium sulphate). The gel was finally conserved at 5 °C in 5% of acetic acid for further experiments.

### 5.5. Shotgun Proteomics of Steatoda nobilis Female Venom

A 0.2 mg sample of lyophilized venom was dissolved in 100 μL of pure water (MilliQ). A 3 μL portion corresponding to roughly 6 µg was lyophilized and dissolved into 20 µL of 50 mM NH_4_HCO_3_ pH 7.8. The sample was then reduced with 5 µL of 500 mM dithiothreitol (DTT) for 45 min at 56 °C under shaking at 300 rpm. The reduced venom was then alkylated with 6 µL of 500 mM iodoacetamide for 30 min, at room temperature, in the dark. The venom was then submitted to enzymatic digestion with trypsin at a ratio of 1:50, incubated overnight, at 37 °C, under shaking at 300 rpm. Reactions were stopped by acidifying the medium using 10% TFA. The digested sample was finally dried on speed vacuum. Before the mass spectrometry analysis, the samples were suspended in 20 µL of 0.1% TFA for desalting on ZipTip pipette tips with C18 resin. The elution was made by 18 μL of TFA 0.1%/ACN (50/50), to reach a theoretical concentration de ~3 μg/9 μL, suitable for LC-MS analysis (9 μL injected, 100 min run). The efficiency of the digestion was controlled by MALDI-TOF, using saturated CHCA (70/30 ACN/FA 0.1 %) as the matrix.

The purified material was analysed using an Acquity UPLC M-Class (Waters, Milford, MA, USA) coupled to the Q-Exactive Plus Hybrid Quadrupole-Orbitrap Mass Spectrometer (Thermo Scientific, Bremen, Germany). The trap column was a Symmetry C18 5 μm (180 μm × 20 mm) and the analytical column was an HSS T3 C18 1.8 μm (75 μm × 250 mm) (Waters, Corp., Milford, MA, USA). The samples were loaded at 20 μL/min on the trap column in 98% solvent A (water/0.1% formic acid) for 3 min and subsequently separated on the analytical column. Peptides were eluted using a gradient of 2–85% of solution B in 73 min (B: acetonitrile/0.1% formic acid), at a flow rate of 0.6 mL/min. Regarding mass spectrometry, all the analyses were performed in data dependent analysis (DDA) mode that automatically triggers the MS/MS experiments. The automatic gain control (AGC) target values were 3.10^6^ for MS spectra and 2.10^5^ for MS/MS spectra. The maximum injection times were set at 200 ms for the MS step and 1000 ms for MS/MS events. For MS/MS, a “Top 12” experiment was applied, meaning that the twelve most intense ions of each MS scan have been selected for fragmentation. Singly charged ions, ions with undetermined charge (for example, electronic noise) and ions with signal intensities below the AGC threshold set at 1 × 10^3^ were excluded from the selection. For precursor ions, the selection windows were 2.0 m/z, the AGC target was 1 × 10^5^ (or 50 ms as a maximum of injection time) and the resolving power of 17,500 @m/z 200. Normalized collision energy was 25. A dynamic exclusion of 10 s was also applied to avoid the redundancy of MS/MS spectra of the same ions. 

Bioinformatic analysis of proteomic data PEAKS Studio X+ (Bioinformatics solutions, Waterloo, ON, Canada), a *de novo* assisted database software [27,28] was chosen to analyse MS/MS data from *Steatoda nobilis* venom. The database chosen for analysing the proteomics data is composed of the translated sequences obtained from the assembled transcriptome. PEAKS studio initially produces *de novo* sequences from MS/MS spectra without relying on a database. The confidence of each peptide sequence obtained by this process is given by an ALC (Average Local Confidence) score. These *de novo* sequences are then corrected by comparing them to the database to provide additional information about post-translational modifications (PTMs), mutations, homologous peptides, and novel peptides. Carbamidomethylation was set as fixed modification, while oxidation (M) was set as a variable modification, with maximum missed cleavages at 3 for trypsin digestion. Parent mass and fragment mass error tolerance were set at 5 ppm and 0.015 Da, respectively. A false discovery rate (FDR) of 1% [29,30] and a unique peptide ≥ 2 were used for filtering out inaccurate proteins. A−10lgP > 120 indicated the detected proteins by enough reliable peptides MS/MS spectra.

## Figures and Tables

**Table 1:** Main toxins identified in the venom of true black widow spiders by Haney et al. [24].

*Latrodectus* Toxins	Abbreviation	Function/Activity
α-latrotoxin	α-LTX	Toxic to vertebrates, forms calcium channels on pre-synaptic neurons, triggers neurotransmitter release.
α- latrocrustotoxin	α-LCT	Toxic to crustaceans, forms calcium channels on pre-synaptic neurons, triggers neurotransmitter release.
α,d-latroinsectotoxin	α-LIT / d-LIT	Toxic to insects, forms calcium channels on pre-synaptic neurons, triggers neurotransmitter release.
Latrodectin	α-LTX LMWPs	Enhances potency of latrotoxins
Cystein Rich Secretory Protein	CRISPs	Block Calcium channels
Metalloprotease	MPs	Tissue lysis, facilitates spread of latrotoxins
Serine protease	SPs	Tissue lysis, facilitates spread of latrotoxins
Hyaluronidase	--	Tissue lysis, facilitates spread of latrotoxins
Chitinase	--	Degrades arthropod exoskeletons
Inhibitor cystine knot	ICK	Alters ion channel function

**Table 2:** The twelve most abundant toxins quantified in the venom.

*Steatoda nobilis* Proteins	Best Match (BLAST)	Accession Number	Species	Quantification in %	Sequence Coverage %	−10lgP
Latrodectin-Sno1a	Latrodectin; Alpha-latrotoxin associated LMWP-2	V9QFH8	*Steatoda grossa*	23.5	63	210.63
Alpha-latrocrustotoxin-Sno1a	Alpha-latrocrustotoxin-Lt1a	Q9XZC0	*Latrodectus tredecimguttatus*	10.6	70	447.19
CRISP-Sno1a	CRISP/Allergen/PR-1-like	XP_015912134	*Parasteatoda tepidariorum*	9.4	81	274.42
Uncharacterized Protein-Sno1a	Uncharacterized protein LOC107442339	XP_015911366	*Parasteatoda tepidariorum*	6.1	66	242.45
Alpha-latrotoxin-Sno1a	Alpha-latrotoxin-Lhe1a	P0DJE3	*Latrodectus hesperus*	4.7	60	295.77
Putative neurotoxin-Sno1a	Putative neurotoxin LTDF 06-01	A0A0K1D8C3	*Dolomedes fimbriatus*	3.7	48	167.43
Delta-latroinsectotoxin-Sno1a	Delta-latroinsectotoxin-Lt1a	Q25338	*Latrodectus tredecimguttatus*	2.8	55	369.06
Latrodectin-Sno1b	Latrodectin; Alpha-latrotoxin associated LMWP-2	V9QFH8	*Steatoda grossa*	2.5	60	222.56
Uncharacterized Protein-Sno1b	Uncharacterized protein LOC107437515	XP_015905073	*Parasteatoda tepidariorum*	2.4	56	175.17
Latrodectin-Sno1c	Latrodectin; Alpha-latrotoxin-associated LMWP	AHC13266.1	*Steatoda grossa*	2.1	63	178.46
Delta-latroinsectotoxin-Sno1b	Delta-latroinsectotoxin-Lt1a	Q25338	*Latrodectus tredecimguttatus*	1.2	47	352.89
CRISP-Sno1b	CRISP/Allergen/PR-1-like	XP_015912134	Parasteatoda tepidariorum	1.1	69	209.5

## Supplementary Materials

The following are available online at https://www.mdpi.com/2072-6651/12/6/402/s1, Dataset S1: Complete Gene Ontology enrichment of highly expressed genes (>100 TPM) in *Steatoda nobilis* venom glands; Dataset S2: Sequence alignments of the main toxins found in *Steatoda nobilis* venom with some already published.
