# Supplementary material for: Venomics Approach Reveals a High Proportion of Lactrodectus-Like Toxins in the Venom of the Noble False Widow Spider Steatoda nobilis
Source: Toxins (Basel). 2020 Jun 18;12(6):402. doi: 10.3390/toxins12060402 (PMC7354476; doi:10.3390/toxins12060402)
Supplement: Supplementary file 1 [file toxins-12-00402-s001.zip › SupplementaryDataset1.pdf]

GO ID	GO term	Ontology Type	Number of Genes	Number of Genes in background	P value	False Discovery Rate
GO:0003735	structural constituent of ribosome	MF	74	152	1.00E-46	1.86E-42
GO:0005840	ribosome	CC	68	145	1.22E-41	1.13E-37
GO:0043043	peptide biosynthetic process	BP	74	190	2.20E-38	1.36E-34
GO:0006412	translation	BP	72	181	4.28E-38	1.98E-34
GO:0006518	peptide metabolic process	BP	82	268	1.56E-33	4.94E-30
GO:0043604	amide biosynthetic process	BP	75	225	1.60E-33	4.94E-30
GO:0044218	other organism cell membrane	CC	52	109	1.58E-32	3.67E-29
GO:0044279	other organism membrane	CC	52	109	1.58E-32	3.67E-29
GO:0044445	cytosolic part	CC	59	146	8.02E-32	1.65E-28
GO:0022625	cytosolic large ribosomal subunit	CC	28	32	2.02E-29	3.74E-26
GO:0044391	ribosomal subunit	CC	56	146	7.35E-29	1.24E-25
GO:0090729	toxin activity	MF	57	154	2.09E-28	3.23E-25
GO:0043603	cellular amide metabolic process	BP	88	357	3.27E-28	4.66E-25
GO:0005198	structural molecule activity	MF	97	459	1.73E-25	2.29E-22
GO:0044217	other organism part	CC	53	169	1.42E-22	1.75E-19
GO:1901566	organonitrogen compound biosynthetic process	BP	105	594	3.58E-21	4.14E-18
GO:0022627	cytosolic small ribosomal subunit	CC	19	22	4.52E-20	4.94E-17
GO:0005622	intracellular	CC	37	108	1.40E-17	1.45E-14
GO:0015934	large ribosomal subunit	CC	34	93	2.92E-17	2.85E-14
GO:0002181	cytoplasmic translation	BP	20	30	3.59E-17	3.33E-14
GO:0006887	exocytosis	BP	59	278	5.51E-16	4.64E-13
GO:1990904	ribonucleoprotein complex	CC	109	756	2.71E-15	2.18E-12
GO:0015935	small ribosomal subunit	CC	23	56	2.22E-13	1.72E-10
GO:0032940	secretion by cell	BP	65	375	4.25E-13	3.15E-10
GO:0005576	extracellular region	CC	108	813	8.34E-13	5.95E-10
GO:0034645	cellular macromolecule biosynthetic process	BP	91	640	1.38E-12	9.49E-10
GO:0044271	cellular nitrogen compound biosynthetic process	BP	109	853	7.48E-12	4.95E-09
GO:0015078	proton transmembrane transporter activity	MF	23	66	1.25E-11	7.99E-09
GO:0046903	secretion	BP	65	410	2.41E-11	1.49E-08
GO:0015985	energy coupled proton transport, down electrochemical	BP	11	14	3.16E-11	1.83E-08
GO:0015986	ATP synthesis coupled proton transport	BP	11	14	3.16E-11	1.83E-08
GO:0006754	ATP biosynthetic process	BP	13	22	1.30E-10	7.28E-08
GO:0009059	macromolecule biosynthetic process	BP	96	754	1.73E-10	9.42E-08
GO:0009201	ribonucleoside triphosphate biosynthetic process	BP	15	31	2.15E-10	1.14E-07
GO:0006457	protein folding	BP	30	124	3.61E-10	1.86E-07
GO:0009145	purine nucleoside triphosphate biosynthetic process	BP	14	28	4.98E-10	2.43E-07
GO:0009206	purine ribonucleoside triphosphate biosynthetic process	BP	14	28	4.98E-10	2.43E-07
GO:0010499	proteasomal ubiquitin-independent protein catabolic process	BP	10	14	1.26E-09	6.01E-07
GO:0004298	threonine-type endopeptidase activity	MF	10	15	3.57E-09	1.57E-06
GO:0070003	threonine-type peptidase activity	MF	10	15	3.57E-09	1.57E-06
GO:0005839	proteasome core complex	CC	10	15	3.57E-09	1.57E-06
GO:0009142	nucleoside triphosphate biosynthetic process	BP	15	37	4.60E-09	1.98E-06
GO:0051082	unfolded protein binding	MF	22	79	5.07E-09	2.13E-06
GO:0005832	chaperonin-containing T-complex	CC	7	7	5.63E-09	2.32E-06
GO:0046034	ATP metabolic process	BP	20	67	6.75E-09	2.72E-06
GO:0098800	inner mitochondrial membrane protein complex	CC	21	74	7.79E-09	3.07E-06
GO:0005344	oxygen carrier activity	MF	8	10	1.48E-08	5.73E-06
GO:0009199	ribonucleoside triphosphate metabolic process	BP	22	85	2.22E-08	8.42E-06
GO:0045263	proton-transporting ATP synthase complex, coupling factor F(o)	CC	7	8	4.25E-08	1.58E-05
GO:0032982	myosin filament	CC	8	11	5.12E-08	1.84E-05
GO:0019843	rRNA binding	MF	17	55	5.16E-08	1.84E-05
GO:0009205	purine ribonucleoside triphosphate metabolic process	BP	21	82	5.67E-08	1.98E-05
GO:0000502	proteasome complex	CC	15	44	7.27E-08	2.40E-05
GO:1902600	proton transmembrane transport	BP	16	50	7.38E-08	2.40E-05
GO:0000276	mitochondrial proton-transporting ATP synthase complex,	CC	6	6	8.52E-08	2.73E-05
GO:0098798	mitochondrial protein complex	CC	22	92	1.04E-07	3.27E-05
GO:0009144	purine nucleoside triphosphate metabolic process	BP	21	86	1.38E-07	4.26E-05
GO:1905369	endopeptidase complex	CC	15	46	1.43E-07	4.34E-05
GO:0004129	cytochrome-c oxidase activity	MF	7	9	1.80E-07	5.14E-05
GO:0015002	heme-copper terminal oxidase activity	MF	7	9	1.80E-07	5.14E-05
GO:0016675	oxidoreductase activity, acting on a heme group of donors	MF	7	9	1.80E-07	5.14E-05
GO:0016676	oxidoreductase activity, acting on a heme group of donors,	MF	7	9	1.80E-07	5.14E-05
GO:0009141	nucleoside triphosphate metabolic process	BP	22	98	3.42E-07	9.60E-05
GO:0033177	proton-transporting two-sector ATPase complex, proton-	CC	9	17	3.66E-07	0.000101328
GO:0044455	mitochondrial membrane part	CC	27	142	5.97E-07	0.000162688

GO:0009127	purine nucleoside monophosphate biosynthetic process	BP	14	45	7.16E-07	0.000189593
GO:0009168	purine ribonucleoside monophosphate biosynthetic process	BP	14	45	7.16E-07	0.000189593
GO:0009126	purine nucleoside monophosphate metabolic process	BP	21	95	8.26E-07	0.000212751
GO:0009167	purine ribonucleoside monophosphate metabolic process	BP	21	95	8.26E-07	0.000212751
GO:0009161	ribonucleoside monophosphate metabolic process	BP	22	105	1.20E-06	0.000304572
GO:0005753	mitochondrial proton-transporting ATP synthase complex	CC	5	5	1.29E-06	0.000314388
GO:0045259	proton-transporting ATP synthase complex	CC	5	5	1.29E-06	0.000314388
GO:0098556	cytoplasmic side of rough endoplasmic reticulum membrane	CC	5	5	1.29E-06	0.000314388
GO:0042788	polysomal ribosome	CC	7	11	1.47E-06	0.000353056
GO:0006613	cotranslational protein targeting to membrane	BP	8	15	1.57E-06	0.000373195
GO:0005615	extracellular space	CC	59	474	1.88E-06	0.000436891
GO:0009156	ribonucleoside monophosphate biosynthetic process	BP	15	55	1.88E-06	0.000436891
GO:0042776	mitochondrial ATP synthesis coupled proton transport	BP	6	8	2.12E-06	0.000486345
GO:0009123	nucleoside monophosphate metabolic process	BP	22	109	2.32E-06	0.000525352
GO:0031404	chloride ion binding	MF	8	16	2.96E-06	0.00066052
GO:0101031	chaperone complex	CC	7	12	3.32E-06	0.000732018
GO:0045047	protein targeting to ER	BP	9	21	3.47E-06	0.000755804
GO:1905368	peptidase complex	CC	17	72	3.51E-06	0.000755804
GO:0030018	Z disc	CC	18	80	3.82E-06	0.000813249
GO:0009124	nucleoside monophosphate biosynthetic process	BP	15	59	4.92E-06	0.001036177
GO:0006614	SRP-dependent cotranslational protein targeting to membrane	BP	7	13	6.77E-06	0.001410863
GO:0070203	regulation of establishment of protein localization to telomere	BP	5	6	7.30E-06	0.001456525
GO:1904851	positive regulation of establishment of protein localization to	BP	5	6	7.30E-06	0.001456525
GO:0045277	respiratory chain complex IV	CC	5	6	7.30E-06	0.001456525
GO:0098554	cytoplasmic side of endoplasmic reticulum membrane	CC	5	6	7.30E-06	0.001456525
GO:0016459	myosin complex	CC	13	47	7.71E-06	0.001520034
GO:0098796	membrane protein complex	CC	49	388	1.02E-05	0.001985359
GO:0016460	myosin II complex	CC	7	14	1.28E-05	0.002461206
GO:0072599	establishment of protein localization to endoplasmic reticulum	BP	9	24	1.29E-05	0.002461206
GO:0006413	translational initiation	BP	12	44	2.02E-05	0.003814691
GO:0070202	regulation of establishment of protein localization to	BP	5	7	2.42E-05	0.004349524
GO:1904816	positive regulation of protein localization to chromosome,	BP	5	7	2.42E-05	0.004349524
GO:0046933	proton-transporting ATP synthase activity, rotational	MF	5	7	2.42E-05	0.004349524
GO:0019773	proteasome core complex, alpha-subunit complex	CC	5	7	2.42E-05	0.004349524
GO:0019774	proteasome core complex, beta-subunit complex	CC	5	7	2.42E-05	0.004349524
GO:0035036	sperm-egg recognition	BP	7	16	3.78E-05	0.006741375
GO:0019829	cation-transporting ATPase activity	MF	11	40	4.10E-05	0.007238682
GO:0009055	electron transfer activity	MF	11	41	5.27E-05	0.009216545
GO:0003746	translation elongation factor activity	MF	7	17	6.06E-05	0.01045382
GO:1904814	regulation of protein localization to chromosome, telomeric	BP	5	8	6.09E-05	0.01045382
GO:0070972	protein localization to endoplasmic reticulum	BP	10	35	6.41E-05	0.010900332
GO:0072594	establishment of protein localization to organelle	BP	28	192	6.99E-05	0.01178527
GO:0007339	binding of sperm to zona pellucida	BP	6	13	9.74E-05	0.016272773
GO:0008135	translation factor activity, RNA binding	MF	15	75	0.000103351	0.017111953
GO:0032991	protein-containing complex	CC	280	3491	0.000112965	0.018538297
GO:0042625	ATPase coupled ion transmembrane transporter activity	MF	11	45	0.000132478	0.021362303
GO:0098803	respiratory chain complex	CC	11	45	0.000132478	0.021362303
GO:0009988	cell-cell recognition	BP	7	19	0.000139545	0.022117356
GO:0022626	cytosolic ribosome	CC	7	19	0.000139545	0.022117356
GO:0044449	contractile fiber part	CC	24	160	0.00014504	0.022793415
GO:1901576	organic substance biosynthetic process	BP	136	1523	0.000156796	0.024433796
GO:0003729	mRNA binding	MF	27	191	0.000159352	0.0246252
GO:0042470	melanosome	CC	14	71	0.000204214	0.031297076
GO:0044249	cellular biosynthetic process	BP	129	1443	0.000226207	0.034383472
GO:0022624	proteasome accessory complex	CC	5	10	0.000244757	0.03612846
GO:0008250	oligosaccharyltransferase complex	CC	5	10	0.000244757	0.03612846
GO:0000028	ribosomal small subunit assembly	BP	5	10	0.000244757	0.03612846
GO:0045214	sarcomere organization	BP	11	48	0.00024548	0.03612846
GO:2001242	regulation of intrinsic apoptotic signaling pathway	BP	13	64	0.000250957	0.036643694
GO:0050821	protein stabilization	BP	17	99	0.000261605	0.037624071
GO:0001178	regulation of transcriptional start site selection at RNA	BP	4	6	0.000261729	0.037624071
GO:0048770	pigment granule	CC	14	73	0.000277079	0.039524254
GO:0044769	ATPase activity, coupled to transmembrane movement of ions,	MF	7	21	0.000286321	0.040223825
GO:0060260	regulation of transcription initiation from RNA polymerase II	BP	7	21	0.000286321	0.040223825
GO:0002862	negative regulation of inflammatory response to antigenic	BP	3	3	0.00029339	0.040906882
