# Supplementary material for: Venomics Approach Reveals a High Proportion of Lactrodectus-Like Toxins in the Venom of the Noble False Widow Spider Steatoda nobilis
Source: Toxins (Basel). 2020 Jun 18;12(6):402. doi: 10.3390/toxins12060402 (PMC7354476; doi:10.3390/toxins12060402)
Supplement: Supplementary file 1 [file toxins-12-00402-s001.zip › SupplementaryDataset2.pdf]

Supplementary dataset 2 – Table1- Latrodectins: Sequence alignments of the closest five latrodectins already published and the five most concentrated ones in the venom of *Steatoda nobilis*. Bold-italics positions indicate the signal peptide. Yellow windows indicate positions where at least 50% of the identity and/or the physico-chemical properties of the aminoacids are maintained, bold positions are the ones where 33 to 50% of the aminoacids are conserved. Cysteines are highlighted in red.

IDENTIFICATION	SPECIES	REF	1	2	3	4	5	6	7	8	9	10	11	12	13	14	15	16	17	18	19	20	21	22	23	24	25	26	27	28	29	30	31	32	33	34	35	36	37	38	39	40	41	42	43	44	45	46	47	48	49	50	51	52
Latrodectin V9QFH8	*S. grossa*	[1]	M	F	K	L	I	C	-	I	V	F	I	A	T	I	L	S	I	T	S	A	A	D	N	E	D	E	L	T	I	E	D	F	L	S	Y	E	C	N	E	S	M	D	I	E	E	L	K	E	K	D	K	V
Latrodectin AHC13269	*S. grossa*	[1]	M	F	K	L	I	C	-	I	V	F	I	A	T	I	L	S	I	T	S	A	A	D	N	E	D	E	L	T	M	E	D	F	L	S	Y	E	C	N	E	S	M	D	I	E	E	L	K	E	K	D	K	V
Latrodectin-2 V9QFG7	*L. geometricus*	[1]	M	L	K	L	I	C	-	I	V	F	L	V	T	V	L	T	F	V	V	G	E	D	T	L	-	-	-	-	-	-	D	P	A	E	Y	G	C	P	S	D	V	D	M	A	E	L	T	E	K	N	E	V
Latrodectin-2 Q4U4N3	*L. tredecimguttatus*	[2]	M	L	K	L	I	C	-	I	A	F	L	V	T	V	L	T	L	V	A	G	Q	D	S	L	-	-	-	-	-	-	D	P	A	E	F	G	C	A	D	D	V	N	Q	A	E	L	L	K	N	N	D	I
Latrodectin-2 AHC13255	*L. hesperus*	[1]	M	L	K	L	I	C	-	I	A	F	L	V	T	V	L	T	L	V	A	G	E	D	S	L	-	-	-	-	-	-	D	P	A	E	Y	G	C	A	D	D	I	N	Q	E	E	L	L	K	K	N	D	V
Latrodectin-2 V9QEI7	*L. hesperus*	[1]	M	L	K	L	I	C	-	I	A	F	L	V	T	V	L	T	L	V	A	G	E	D	S	L	-	-	-	-	-	-	D	P	A	E	Y	G	C	A	D	D	I	N	Q	E	D	L	L	K	K	N	D	V
Latrodectin-Sno1a	*S.nobilis*	*This study*	M	F	K	L	I	W	T	V	V	F	L	A	I	I	L	S	F	T	S	A	T	D	D	D	K	A	V	N	V	A	D	F	G	N	Y	G	C	D	D	N	M	D	I	G	E	F	K	E	K	D	A	T
Latrodectin-Sno1b	*S.nobilis*	*This study*	M	G	K	L	H	F	-	-	-	-	L	I	L	L	S	V	I	V	S	-	-	-	-	-	-	-	V	F	C	I	E	P	E	D	I	G	C	Q	-	G	I	S	R	E	E	F	D	K	K	D	A	E
Latrodectin-Sno1c	*S.nobilis*	*This study*	M	N	T	L	H	F	-	-	-	-	L	I	L	L	M	S	V	V	S	-	-	-	-	-	-	-	V	F	C	I	S	P	A	D	I	G	C	T	-	D	I	S	Q	E	E	F	D	E	K	D	S	K
Latrodectin-Sno1d	*S.nobilis*	*This study*	-	-	-	-	-	-	-	-	-	-	-	-	-	-	-	-	-	T	S	A	T	D	D	D	E	A	V	N	V	A	D	F	G	N	F	G	C	E	N	N	M	D	I	G	E	F	K	E	K	D	T	A
Latrodectin-Sno1e	*S.nobilis*	*This study*	M	F	K	L	I	C	-	I	V	F	L	A	T	I	L	S	I	T	S	A	T	-	E	D	D	S	L	T	I	D	D	F	L	S	H	G	C	D	E	S	M	D	I	E	E	L	K	Q	K	D	N	V
Latrodectin-Sno1f	*S.nobilis*	*This study*	M	F	K	L	I	C	-	I	V	F	L	A	T	I	L	S	I	T	S	A	T	-	E	D	D	S	L	T	I	D	D	F	L	S	H	G	C	D	E	S	M	D	I	E	E	L	K	Q	K	D	N	V

IDENTIFICATION	SPECIES	REF	53	54	55	56	57	58	59	60	61	62	63	64	65	66	67	68	69	70	71	72	73	74	75	76	77	78	79	80	81	82	83	84	85	86	87	88	89	90	91	92	93	94	95	96	97	98	99	100	101	102	103
Latrodectin V9QFH8	S. grossa	[1]	C	S	R	C	A	N	L	H	K	T	Q	S	V	I	E	R	C	R	L	N	C	F	T	S	E	Y	F	K	N	C	E	D	N	L	Q	A	K	E	E	E	P	E	E	E	T	L	-	-	-	-	-
Latrodectin AHC13269	S. grossa	[1]	C	S	R	C	A	N	L	H	K	T	Q	S	V	I	E	R	C	R	L	N	C	F	T	S	E	Y	F	K	N	C	E	D	N	L	Q	T	K	E	E	E	P	E	E	E	T	L	-	-	-	-	-
Latrodectin-2 V9QFG7	L. geometricus	[1]	C	L	R	C	E	D	F	H	K	E	G	V	A	F	T	L	C	K	T	N	C	F	T	T	E	Y	Y	K	N	C	V	K	D	L	E	E	A	G	K	E	T	E	E	-	-	-	-	-	-	-	-
Latrodectin-2 Q4U4N3	L. tredecimguttatus	[2]	C	L	Q	C	E	D	L	H	K	E	G	V	V	F	S	L	C	K	T	N	C	F	T	T	E	Y	F	Q	H	C	V	K	D	L	E	E	A	K	K	E	P	P	E	-	-	-	-	-	-	-	-
Latrodectin-2 AHC13255	L. hesperus	[1]	C	L	Q	C	E	D	L	H	K	E	G	V	V	F	S	L	C	K	T	N	C	F	T	T	Q	Y	F	T	N	C	V	K	D	L	E	E	A	E	K	E	P	P	E	-	-	-	-	-	-	-	-
Latrodectin-2 V9QEI7	L. hesperus	[1]	C	L	Q	C	E	D	L	H	K	E	G	V	V	F	S	L	C	K	T	N	C	F	T	T	Q	Y	F	T	N	C	V	K	D	L	E	E	A	E	K	E	P	P	E	-	-	-	-	-	-	-	-
Latrodectin-Sno1a	S.nobilis	*This study*	C	T	E	C	G	Y	L	H	N	A	H	P	A	N	D	M	C	R	S	Q	C	F	K	S	E	Y	F	K	Y	C	Q	D	T	L	Q	G	K	D	K	E	V	E	E	E	T	F	-	-	-	-	-
Latrodectin-Sno1b	S.nobilis	*This study*	C	H	Q	C	G	E	D	Y	S	E	T	D	M	I	D	K	C	R	S	N	C	F	S	G	S	F	Y	K	S	C	V	D	L	I	N	K	V	Y	D	E	K	D	A	N	E	K	P	D	W	-	-
Latrodectin-Sno1c	S.nobilis	*This study*	C	N	K	C	N	D	G	F	E	D	T	N	M	L	E	R	C	R	A	D	C	F	K	G	K	F	F	R	S	C	V	D	H	L	E	G	I	Y	D	E	K	D	N	V	E	G	P	P	P	E	M
Latrodectin-Sno1d	S.nobilis	*This study*	C	T	E	C	G	Y	L	H	N	A	H	S	A	N	D	M	C	R	S	Q	C	F	K	S	E	Y	F	K	Y	C	Q	D	T	L	Q	G	K	D	-	-	-	-	-	-	-	-	-	-	-	-	-
Latrodectin-Sno1e	S.nobilis	*This study*	C	S	R	C	G	Y	L	H	S	A	Y	S	A	N	D	L	C	R	S	A	C	F	N	S	E	Y	F	K	E	C	E	A	N	L	Q	I	K	E	E	D	T	E	E	E	T	S	-	-	-	-	-
Latrodectin-Sno1f	S.nobilis	*This study*	C	S	Q	C	G	Y	F	H	S	A	H	S	V	N	D	L	C	R	S	A	C	F	N	S	E	Y	F	K	E	C	E	A	N	L	Q	I	K	E	E	D	T	E	E	E	T	S	-	-	-	-	-

References

[1] Mc Cowan C. and Garb J.E., 2014, Gene, 536(2), 366-375.

[2] Volkova T.M. et al., 1995, Toxicon, 33(4), 483-489.

Supplementary dataset 2 – Table2a- Alpha-Latrocrustotoxins- Sequences from position 01 to 1167: Sequence alignments of the closest two alpha-latrocrustotoxins already published and the one found in the venom of *Steatoda nobilis*. Yellow windows indicate positions where the identity and/or the physico-chemical properties of the aminoacids are fully maintained, bold positions are the ones where 66% of the aminoacids are conserved. Cysteines are highlighted in red.

IDENTIFICATION	SPECIES	REF	1	2	3	4	5	6	7	8	9	10	11	12	13	14	15	16	17	18	19	20	21	22	23	24	25	26	27	28	29	30	31	32	33	34	35	36	37	38	39	40	41	42	43	44	45	46	47	48	49	50
Alpha-Lacrustotoxin-Lt1a Q9XZC0	L.tredecimguttatus	[1]	E	M	S	K	A	D	Q	C	T	F	L	S	Y	Q	S	V	A	Y	G	T	L	G	D	V	A	G	D	V	S	S	I	E	G	A	D	L	V	A	T	P	I	A	A	G	G	H	L	A	K	G
Alpha-Lacrustotoxin-Lt1a Q02989	L.tredecimguttatus	[1]	E	M	S	R	A	D	Q	C	K	L	L	A	Y	T	A	V	G	Y	E	T	V	G	N	V	A	A	D	I	A	S	I	E	G	A	N	L	V	A	A	P	V	A	A	G	G	H	L	G	K	G
Alpha-Latrocrustotoxin-Sno1a	S. nobilis	This study	E	M	T	R	A	E	Q	C	T	I	L	Q	Y	T	S	I	G	Y	D	T	V	G	N	V	A	A	D	I	S	S	I	D	G	V	N	L	V	G	A	P	I	A	A	G	S	H	L	G	K	G

IDENTIFICATION	SPECIES	REF	51	52	53	54	55	56	57	58	59	60	61	62	63	64	65	66	67	68	69	70	71	72	73	74	75	76	77	78	79	80	81	82	83	84	85	86	87	88	89	90	91	92	93	94	95	96	97	98	99	100
Alpha-Lacrustotoxin-Lt1a Q9XZC0	L.tredecimguttatus	[1]	A	T	D	A	A	M	I	A	M	D	C	S	S	I	P	F	D	E	I	K	Q	Q	L	N	Q	R	F	N	E	V	D	K	K	L	Q	K	G	A	E	A	L	E	N	V	T	E	L	A	E	K
Alpha-Lacrustotoxin-Lt1a Q02989	L.tredecimguttatus	[1]	L	T	D	A	A	M	I	A	M	D	C	S	S	I	P	F	E	E	I	K	E	I	L	N	K	E	F	K	E	M	G	R	K	L	D	K	N	T	E	A	L	E	H	V	S	K	L	V	S	K
Alpha-Latrocrustotoxin-Sno1a	S. nobilis	This study	M	T	D	A	A	M	L	A	M	D	C	A	S	I	P	F	E	E	I	K	Q	I	L	N	K	R	F	N	E	L	D	K	K	L	D	K	G	A	E	A	L	E	S	V	S	K	L	V	D	K

IDENTIFICATION	SPECIES	REF	101	102	103	104	105	106	107	108	109	110	111	112	113	114	115	116	117	118	119	120	121	122	123	124	125	126	127	128	129	130	131	132	133	134	135	136	137	138	139	140	141	142	143	144	145	146	147	148	149	150
Alpha-Lacrustotoxin-Lt1a Q9XZC0	L.tredecimguttatus	[1]	T	Y	S	S	V	E	K	M	R	V	E	M	R	E	G	F	N	H	V	I	A	T	I	E	N	A	N	T	K	Q	I	I	T	G	I	N	Q	I	I	Q	Y	F	N	D	E	R	E	N	I	N
Alpha-Lacrustotoxin-Lt1a Q02989	L.tredecimguttatus	[1]	T	L	S	T	V	E	K	I	R	V	E	M	R	E	G	F	K	L	V	I	E	T	I	E	N	I	A	T	K	E	I	V	F	D	I	N	K	I	V	Q	Y	F	N	N	E	R	E	N	I	N
Alpha-Latrocrustotoxin-Sno1a	S. nobilis	This study	T	Y	R	T	V	E	K	V	R	F	E	M	R	E	G	F	K	R	V	T	E	T	I	E	N	A	A	T	K	Q	I	I	S	Q	I	N	E	I	V	Q	Y	F	S	D	E	K	E	N	I	N

IDENTIFICATION	SPECIES	REF	151	152	153	154	155	156	157	158	159	160	161	162	163	164	165	166	167	168	169	170	171	172	173	174	175	176	177	178	179	180	181	182	183	184	185	186	187	188	189	190	191	192	193	194	195	196	197	198	199	200
Alpha-Lacrustotoxin-Lt1a Q9XZC0	L.tredecimguttatus	[1]	N	R	Q	K	E	D	Y	V	A	K	L	Q	E	P	A	S	G	N	F	L	L	Y	L	R	K	S	R	T	S	E	D	G	S	L	H	S	L	L	F	K	I	I	N	Q	E	L	A	I	P	N
Alpha-Lacrustotoxin-Lt1a Q02989	L.tredecimguttatus	[1]	S	R	Q	K	E	E	F	V	A	K	L	Q	E	P	A	P	G	N	F	L	L	Y	L	R	N	S	R	T	S	E	S	G	T	L	Y	S	L	L	F	R	I	I	D	Q	E	L	A	I	P	N
Alpha-Latrocrustotoxin-Sno1a	S. nobilis	This study	N	R	Q	K	E	D	Y	V	A	K	L	R	E	P	D	S	G	N	F	L	L	Y	L	R	K	S	R	T	L	E	E	G	S	L	H	S	L	L	F	K	I	I	N	Q	E	S	A	I	P	N

IDENTIFICATION	SPECIES	REF	201	202	203	204	205	206	207	208	209	210	211	212	213	214	215	216	217	218	219	220	221	222	223	224	225	226	227	228	229	230	231	232	233	234	235	236	237	238	239	240	241	242	243	244	245	246	247	248	249	250
Alpha-Lacrustotoxin-Lt1a Q9XZC0	L.tredecimguttatus	[1]	N	A	A	D	N	N	A	I	R	A	L	F	A	L	F	Y	G	T	Q	T	F	I	S	I	M	F	Y	L	V	K	Q	Y	S	Y	L	A	D	Y	H	Y	Q	N	G	N	L	A	E	F	N	S
Alpha-Lacrustotoxin-Lt1a Q02989	L.tredecimguttatus	[1]	N	A	G	D	N	N	A	I	Q	A	L	Y	A	L	F	Y	G	T	E	T	F	I	S	I	M	F	Y	L	V	K	Q	Y	S	Y	L	A	E	Y	H	Y	Q	K	G	N	L	E	E	F	N	T
Alpha-Latrocrustotoxin-Sno1a	S. nobilis	This study	K	A	G	D	D	N	A	V	R	A	L	Y	A	L	F	Y	G	T	Q	T	Y	I	S	I	M	L	Y	L	V	K	Q	Y	S	Y	L	A	D	Y	F	Y	Q	E	G	N	L	E	E	F	N	E

IDENTIFICATION	SPECIES	REF	251	252	253	254	255	256	257	258	259	260	261	262	263	264	265	266	267	268	269	270	271	272	273	274	275	276	277	278	279	280	281	282	283	284	285	286	287	288	289	290	291	292	293	294	295	296	297	298	299	300
Alpha-Lacrustotoxin-Lt1a Q9XZC0	L.tredecimguttatus	[1]	N	F	D	H	M	K	T	V	F	Q	D	F	K	F	T	L	I	G	I	N	T	S	N	S	K	P	L	V	N	T	V	L	S	I	I	E	D	V	K	N	K	R	F	I	R	N	L	R	S	N
Alpha-Lacrustotoxin-Lt1a Q02989	L.tredecimguttatus	[1]	N	F	D	H	M	K	I	V	F	Q	D	F	K	F	S	L	I	G	I	N	-	Q	N	T	K	P	L	V	D	E	V	L	N	V	L	N	N	V	K	N	K	S	F	I	R	N	V	Q	N	K
Alpha-Latrocrustotoxin-Sno1a	S. nobilis	This study	N	F	T	H	M	K	T	V	F	K	D	F	K	F	S	L	I	G	I	R	G	Q	S	G	T	P	L	V	D	S	V	I	H	V	L	E	T	V	K	G	K	S	F	I	R	N	L	K	N	N

IDENTIFICATION	SPECIES	REF	301	302	303	304	305	306	307	308	309	310	311	312	313	314	315	316	317	318	319	320	321	322	323	324	325	326	327	328	329	330	331	332	333	334	335	336	337	338	339	340	341	342	343	344	345	346	347	348	349	350
Alpha-Lacrustotoxin-Lt1a Q9XZC0	L.tredecimguttatus	[1]	L	Y	Q	K	I	I	K	S	T	K	S	L	L	D	L	R	E	K	I	T	K	M	D	L	P	I	I	E	D	T	P	K	S	S	-	V	L	I	N	F	R	E	K	S	S	S	V	P	R	I
Alpha-Lacrustotoxin-Lt1a Q02989	L.tredecimguttatus	[1]	L	F	Y	D	L	M	K	Q	T	E	S	L	L	E	L	K	K	E	I	A	N	M	E	L	P	I	I	D	E	T	P	R	L	S	-	-	I	S	I	S	F	K	E	R	S	D	D	K	P	V
Alpha-Latrocrustotoxin-Sno1a	S. nobilis	This study	L	Y	Q	D	I	L	K	Q	T	D	S	L	L	K	L	K	T	E	I	A	D	M	E	L	P	I	I	D	E	T	P	K	S	S	S	V	I	I	S	F	K	E	K	S	P	S	V	V	Q	V

IDENTIFICATION	SPECIES	REF	351	352	353	354	355	356	357	358	359	360	361	362	363	364	365	366	367	368	369	370	371	372	373	374	375	376	377	378	379	380	381	382	383	384	385	386	387	388	389	390	391	392	393	394	395	396	397	398	399	400
Alpha-Lacrustotoxin-Lt1a Q9XZC0	L.tredecimguttatus	[1]	E	T	P	I	L	K	W	T	P	G	T	V	V	K	Y	A	I	Q	Y	E	Q	D	G	K	Y	S	K	I	S	K	W	S	N	P	I	T	V	Q	R	L	A	N	P	Y	I	T	I	D	K	D
Alpha-Lacrustotoxin-Lt1a Q02989	L.tredecimguttatus	[1]	D	T	P	L	L	K	W	D	K	G	K	E	V	K	Y	A	I	Q	F	E	Q	D	G	K	F	S	K	I	S	S	W	S	K	P	V	T	V	Q	H	L	A	C	P	F	I	S	V	D	K	D
Alpha-Latrocrustotoxin-Sno1a	S. nobilis	This study	E	T	P	I	L	K	W	N	P	G	T	D	V	K	Y	A	V	Q	Y	E	Q	D	G	K	Y	S	K	I	S	S	W	S	N	P	I	T	I	Q	G	L	A	C	P	F	I	T	I	D	K	S

IDENTIFICATION	SPECIES	REF	401	402	403	404	405	406	407	408	409	410	411	412	413	414	415	416	417	418	419	420	421	422	423	424	425	426	427	428	429	430	431	432	433	434	435	436	437	438	439	440	441	442	443	444	445	446	447	448	449	450
Alpha-Lacrustotoxin-Lt1a Q9XZC0	L.tredecimguttatus	[1]	R	R	N	R	L	V	F	R	Q	F	G	N	E	K	P	E	L	I	S	I	L	D	S	S	Q	N	E	F	R	D	I	H	R	D	L	Y	N	A	A	Q	M	P	Y	K	E	T	A	L	G	I
Alpha-Lacrustotoxin-Lt1a Q02989	L.tredecimguttatus	[1]	R	R	N	R	L	I	F	R	Q	F	G	D	Q	I	P	E	L	V	G	T	L	R	G	S	Q	V	E	F	R	D	I	H	R	D	L	Y	N	A	A	Q	V	P	Y	A	R	E	A	L	S	I
Alpha-Latrocrustotoxin-Sno1a	S. nobilis	This study	P	R	N	R	L	V	F	R	Q	F	G	S	Q	T	P	E	L	V	A	I	L	N	G	S	Q	N	E	F	R	D	I	H	R	D	L	F	N	A	A	Q	M	L	M	R	E	N	A	A	T	T

IDENTIFICATION	SPECIES	REF	451	452	453	454	455	456	457	458	459	460	461	462	463	464	465	466	467	468	469	470	471	472	473	474	475	476	477	478	479	480	481	482	483	484	485	486	487	488	489	490	491	492	493	494	495	496	497	498	499	500
Alpha-Lacrustotoxin-Lt1a Q9XZC0	L.tredecimguttatus	[1]	C	R	K	L	I	D	S	G	A	Q	V	G	A	S	F	E	M	G	R	K	S	I	H	A	S	A	T	A	G	N	D	D	V	A	R	L	L	L	A	K	N	N	G	L	L	N	V	P	D	K
Alpha-Lacrustotoxin-Lt1a Q02989	L.tredecimguttatus	[1]	S	R	T	L	I	Q	N	G	A	N	V	S	E	T	F	E	L	G	R	G	A	I	H	A	A	A	S	A	G	N	Y	D	V	G	E	L	L	L	N	K	D	I	N	L	L	E	K	A	D	K
Alpha-Latrocrustotoxin-Sno1a	S. nobilis	This study	C	Q	Q	L	I	N	S	G	A	D	V	S	A	S	F	E	M	G	R	K	T	I	H	A	A	A	S	V	G	N	D	N	V	A	S	L	L	L	E	Q	N	S	G	L	L	N	A	A	D	K

IDENTIFICATION	SPECIES	REF	501	502	503	504	505	506	507	508	509	510	511	512	513	514	515	516	517	518	519	520	521	522	523	524	525	526	527	528	529	530	531	532	533	534	535	536	537	538	539	540	541	542	543	544	545	546	547	548	549	550
Alpha-Lacrustotoxin-Lt1a Q9XZC0	L.tredecimguttatus	[1]	N	G	Y	T	P	L	H	I	A	S	E	R	K	N	N	D	F	V	K	F	L	L	E	K	G	A	D	V	N	V	R	T	F	A	N	E	L	T	P	L	H	L	A	A	R	Q	D	F	T	I
Alpha-Lacrustotoxin-Lt1a Q02989	L.tredecimguttatus	[1]	N	G	Y	T	P	L	H	I	A	A	D	S	N	K	N	D	F	V	M	F	L	I	G	N	N	A	D	V	N	V	R	T	K	S	D	L	F	T	P	L	H	L	A	A	R	R	D	L	T	D
Alpha-Latrocrustotoxin-Sno1a	S. nobilis	This study	N	G	Y	T	P	L	H	I	A	S	E	S	K	K	N	D	F	V	K	L	L	L	E	K	G	A	N	V	N	A	Q	T	S	L	N	L	L	T	P	L	H	L	A	A	R	R	D	F	T	I

IDENTIFICATION	SPECIES	REF	551	552	553	554	555	556	557	558	559	560	561	562	563	564	565	566	567	568	569	570	571	572	573	574	575	576	577	578	579	580	581	582	583	584	585	586	587	588	589	590	591	592	593	594	595	596	597	598	599	600
Alpha-Lacrustotoxin-Lt1a Q9XZC0	L.tredecimguttatus	[1]	I	V	K	T	L	M	E	K	R	G	I	D	V	N	A	K	E	R	A	G	F	T	P	L	H	L	S	I	T	S	N	S	R	A	A	R	T	L	I	N	E	T	P	A	G	I	N	I	K	S
Alpha-Lacrustotoxin-Lt1a Q02989	L.tredecimguttatus	[1]	V	T	Q	T	L	I	D	I	T	E	I	D	L	N	A	Q	D	K	S	G	F	T	P	L	H	L	S	I	S	S	T	S	E	T	A	A	I	L	I	R	N	T	N	A	V	I	N	I	K	S
Alpha-Latrocrustotoxin-Sno1a	S. nobilis	This study	I	V	R	T	L	V	K	N	K	D	V	D	L	N	A	K	E	H	A	G	F	T	P	L	H	V	S	I	T	S	N	F	R	T	A	R	T	L	I	R	D	T	D	A	N	I	N	I	K	S

IDENTIFICATION	SPECIES	REF	601	602	603	604	605	606	607	608	609	610	611	612	613	614	615	616	617	618	619	620	621	622	623	624	625	626	627	628	629	630	631	632	633	634	635	636	637	638	639	640	641	642	643	644	645	646	647	648	649	650
Alpha-Lacrustotoxin-Lt1a Q9XZC0	L.tredecimguttatus	[1]	N	S	G	L	T	P	L	H	L	A	V	L	Q	N	N	L	S	A	A	K	V	L	V	K	S	N	K	K	V	K	L	N	E	M	D	N	N	G	M	T	P	L	H	Y	A	S	M	L	G	N
Alpha-Lacrustotoxin-Lt1a Q02989	L.tredecimguttatus	[1]	K	V	G	L	T	P	L	H	L	A	T	L	Q	N	N	L	S	V	S	K	L	L	A	G	-	-	K	G	A	Y	L	N	D	G	D	A	N	G	M	T	P	L	H	Y	A	A	M	T	G	N
Alpha-Latrocrustotoxin-Sno1a	S. nobilis	This study	N	F	G	L	T	P	L	H	L	A	V	L	Q	N	N	L	N	A	A	K	V	L	T	K	S	N	K	G	I	N	L	N	E	A	D	D	N	G	M	T	P	L	H	Y	A	A	M	T	G	S

IDENTIFICATION	SPECIES	REF	651	652	653	654	655	656	657	658	659	660	661	662	663	664	665	666	667	668	669	670	671	672	673	674	675	676	677	678	679	680	681	682	683	684	685	686	687	688	689	690	691	692	693	694	695	696	697	698	699	700
Alpha-Lacrustotoxin-Lt1a Q9XZC0	L.tredecimguttatus	[1]	L	E	F	V	K	Y	F	T	S	E	Q	G	I	D	V	N	A	K	T	K	V	K	N	W	T	P	L	H	L	A	I	L	F	K	K	F	D	V	A	Q	S	L	L	Q	V	R	N	I	D	I
Alpha-Lacrustotoxin-Lt1a Q02989	L.tredecimguttatus	[1]	L	E	M	V	D	F	L	L	N	Q	Q	Y	I	N	I	N	A	A	T	K	E	K	K	W	T	P	L	H	L	A	I	L	F	K	K	N	D	V	A	E	R	L	L	S	D	E	N	L	N	I
Alpha-Latrocrustotoxin-Sno1a	S. nobilis	This study	L	T	L	T	I	F	L	M	A	Q	S	G	I	D	Y	N	A	K	T	K	E	K	N	W	T	P	L	H	L	A	V	L	F	K	H	F	D	V	A	E	R	L	L	M	E	K	N	L	D	V

IDENTIFICATION	SPECIES	REF	701	702	703	704	705	706	707	708	709	710	711	712	713	714	715	716	717	718	719	720	721	722	723	724	725	726	727	728	729	730	731	732	733	734	735	736	737	738	739	740	741	742	743	744	745	746	747	748	749	750
Alpha-Lacrustotoxin-Lt1a Q9XZC0	L.tredecimguttatus	[1]	S	T	R	A	D	Q	A	I	T	P	L	H	L	A	A	A	T	G	N	S	Q	I	V	K	T	I	L	N	S	G	A	V	V	D	Q	E	T	A	N	G	F	T	A	L	H	L	A	I	M	N
Alpha-Lacrustotoxin-Lt1a Q02989	L.tredecimguttatus	[1]	R	L	E	T	N	G	G	I	N	P	L	H	L	A	S	A	T	G	N	K	Q	L	V	I	E	L	L	A	K	N	A	D	V	T	R	L	T	S	K	G	F	S	A	L	H	L	G	I	I	G
Alpha-Latrocrustotoxin-Sno1a	S. nobilis	This study	N	I	G	A	G	E	G	V	T	P	F	H	L	A	A	A	T	G	N	Q	Q	L	V	E	D	I	L	N	R	G	V	D	I	N	I	Q	T	I	K	G	L	T	A	L	H	L	A	I	M	N

Supplementary dataset 2 – Table2b- Alpha-Latrocrustotoxins- Sequences from position 701 to 700: Sequence alignments of the closest two alpha-latrocrustotoxins already published and the one found in the venom of *Steatoda nobilis*. Yellow windows indicate positions where the identity and/or the physico-chemical properties of the aminoacids are fully maintained, bold positions are the ones where 66% of the aminoacids are conserved. Cysteines are highlighted in red.

IDENTIFICATION	SPECIES	REF	751	752	753	754	755	756	757	758	759	760	761	762	763	764	765	766	767	768	769	770	771	772	773	774	775	776	777	778	779	780	781	782	783	784	785	786	787	788	789	790	791	792	793	794	795	796	797	798	799	800
Alpha-Lacrustotoxin-Lt1a Q9XZC0	*L.tredecimguttatus*	[1]	P	N	T	E	T	P	Q	F	L	I	A	K	G	A	N	I	N	A	K	T	N	D	G	S	T	P	L	H	F	A	A	A	L	G	K	T	N	I	F	Q	L	L	M	D	K	G	A	N	I	K
Alpha-Lacrustotoxin-Lt1a Q02989	*L.tredecimguttatus*	[1]	K	N	E	E	I	P	F	F	L	V	E	K	G	A	N	V	N	D	K	T	N	S	G	V	T	P	L	H	F	A	A	G	L	G	K	A	N	I	F	R	L	L	L	S	R	G	A	D	I	K
Alpha-Latrocrustotoxin-Sno1a	*S. nobilis*	*This study*	K	N	E	A	T	P	P	F	L	I	E	K	G	A	N	I	N	A	K	T	N	F	G	A	T	P	L	H	L	A	A	A	L	G	K	V	S	I	F	K	T	L	L	G	K	G	A	N	I	K

IDENTIFICATION	SPECIES	REF	801	802	803	804	805	806	807	808	809	810	811	812	813	814	815	816	817	818	819	820	821	822	823	824	825	826	827	828	829	830	831	832	833	834	835	836	837	838	839	840	841	842	843	844	845	846	847	848	849	850
Alpha-Lacrustotoxin-Lt1a Q9XZC0	*L.tredecimguttatus*	[1]	A	E	N	L	I	N	Q	M	P	I	H	E	A	V	V	N	G	H	L	A	I	V	K	M	L	I	E	Q	D	S	S	L	M	N	A	K	N	M	R	D	E	Y	P	F	Y	L	A	A	E	K
Alpha-Lacrustotoxin-Lt1a Q02989	*L.tredecimguttatus*	[1]	A	E	D	I	N	S	Q	M	P	I	H	E	A	V	S	N	G	H	L	E	I	V	R	I	L	I	E	K	D	P	S	L	M	N	V	K	N	I	R	N	E	Y	P	F	Y	L	A	V	E	K
Alpha-Latrocrustotoxin-Sno1a	*S. nobilis*	*This study*	A	Q	D	I	N	L	Q	M	P	I	H	E	A	V	S	N	G	H	L	E	I	V	Q	I	L	I	D	K	D	S	S	L	M	N	E	K	N	I	R	Q	E	Y	P	F	Y	L	A	A	E	K

IDENTIFICATION	SPECIES	REF	851	852	853	854	855	856	857	858	859	860	861	862	863	864	865	866	867	868	869	870	871	872	873	874	875	876	877	878	879	880	881	882	883	884	885	886	887	888	889	890	891	892	893	894	895	896	897	898	899	900
Alpha-Lacrustotoxin-Lt1a Q9XZC0	*L.tredecimguttatus*	[1]	R	Y	K	D	V	F	N	Y	L	E	S	K	G	A	D	V	N	E	K	N	N	D	G	N	T	L	L	H	L	F	S	I	N	G	E	V	E	V	V	Q	F	L	I	Q	N	G	A	D	F	R
Alpha-Lacrustotoxin-Lt1a Q02989	*L.tredecimguttatus*	[1]	R	Y	K	D	I	F	D	Y	F	V	S	K	D	A	N	V	N	E	V	D	H	N	G	N	T	L	L	H	L	F	S	S	T	G	E	L	E	V	V	Q	F	L	M	Q	N	G	A	N	F	R
Alpha-Latrocrustotoxin-Sno1a	*S. nobilis*	*This study*	R	Y	K	D	V	F	N	Y	L	E	S	K	G	A	D	V	N	E	K	D	E	E	G	S	T	L	L	H	L	F	S	T	T	G	E	L	E	V	V	Q	F	L	I	Q	N	G	A	D	F	R

IDENTIFICATION	SPECIES	REF	901	902	903	904	905	906	907	908	909	910	911	912	913	914	915	916	917	918	919	920	921	922	923	924	925	926	927	928	929	930	931	932	933	934	935	936	937	938	939	940	941	942	943	944	945	946	947	948	949	950
Alpha-Lacrustotoxin-Lt1a Q9XZC0	*L.tredecimguttatus*	[1]	L	R	N	K	E	R	K	S	F	F	D	L	A	V	E	F	G	H	A	G	I	V	G	Y	A	I	E	E	N	K	V	D	L	Q	E	P	Y	R	G	K	T	I	L	Y	H	A	I	C	D	S
Alpha-Lacrustotoxin-Lt1a Q02989	*L.tredecimguttatus*	[1]	L	K	N	N	E	R	K	T	F	F	D	L	A	I	E	N	G	R	L	N	I	V	A	F	A	V	E	K	N	K	V	N	L	Q	A	A	H	R	G	K	T	I	L	Y	H	A	I	C	D	S
Alpha-Latrocrustotoxin-Sno1a	*S. nobilis*	*This study*	L	R	N	K	K	G	K	N	F	F	D	F	A	V	E	N	G	H	A	N	I	V	T	F	A	V	D	E	N	K	V	D	L	H	E	K	Y	K	G	E	N	V	L	Y	H	A	L	C	D	A

IDENTIFICATION	SPECIES	REF	951	952	953	954	955	956	957	958	959	960	961	962	963	964	965	966	967	968	969	970	971	972	973	974	975	976	977	978	979	980	981	982	983	984	985	986	987	988	989	990	991	992	993	994	995	996	997	998	999	1000
Alpha-Lacrustotoxin-Lt1a Q9XZC0	*L.tredecimguttatus*	[1]	V	K	Y	D	R	I	E	V	V	R	Y	F	V	E	T	L	N	E	D	Q	C	S	P	L	Q	E	A	A	A	Y	A	H	L	D	L	H	K	I	D	V	N	T	R	N	K	E	Q	-	F	T
Alpha-Lacrustotoxin-Lt1a Q02989	*L.tredecimguttatus*	[1]	A	K	Y	D	K	I	E	I	V	K	Y	F	I	E	K	L	N	E	S	E	C	N	P	L	H	E	A	A	A	Y	A	H	L	D	L	H	K	I	D	V	N	T	R	N	K	E	Q	-	F	T
Alpha-Latrocrustotoxin-Sno1a	*S. nobilis*	*This study*	V	K	Y	D	S	F	E	V	V	K	Y	F	V	E	T	M	N	E	V	V	C	S	P	L	H	E	A	A	A	Y	A	H	L	D	L	V	K	Y	F	V	Q	E	R	G	I	H	P	S	A	F

IDENTIFICATION	SPECIES	REF	1001	1002	1003	1004	1005	1006	1007	1008	1009	1010	1011	1012	1013	1014	1015	1016	1017	1018	1019	1020	1021	1022	1023	1024	1025	1026	1027	1028	1029	1030	1031	1032	1033	1034	1035	1036	1037	1038	1039	1040	1041	1042	1043	1044	1045	1046	1047	1048	1049	1050
Alpha-Lacrustotoxin-Lt1a Q9XZC0	*L.tredecimguttatus*	[1]	P	L	H	A	A	A	S	N	D	H	I	D	V	V	K	Y	L	I	Q	K	G	A	D	V	N	A	K	G	D	E	N	L	K	P	I	D	L	A	G	E	K	S	K	A	Y	E	C	D	T	Q
Alpha-Lacrustotoxin-Lt1a Q02989	*L.tredecimguttatus*	[1]	P	L	H	A	A	A	S	N	D	H	I	D	V	V	K	Y	L	I	Q	K	G	A	D	V	N	A	K	G	D	E	N	L	K	P	I	D	L	A	G	E	K	S	K	A	Y	K	C	D	V	Q
Alpha-Latrocrustotoxin-Sno1a	*S. nobilis*	*This study*	D	E	E	N	Q	V	S	P	L	C	L	A	V	S	G	N	V	C	G	V	L	V	S	C	D	T	P	E	R	L	D	V	V	K	Y	L	A	G	K	T	T	D	L	N	R	E	C	D	T	Q

IDENTIFICATION	SPECIES	REF	1051	1052	1053	1054	1055	1056	1057	1058	1059	1060	1061	1062	1063	1064	1065	1066	1067	1068	1069	1070	1071	1072	1073	1074	1075	1076	1077	1078	1079	1080	1081	1082	1083	1084	1085	1086	1087	1088	1089	1090	1091	1092	1093	1094	1095	1096	1097	1098	1099	1100
Alpha-Lacrustotoxin-Lt1a Q9XZC0	*L.tredecimguttatus*	[1]	Q	S	T	P	V	S	S	A	V	Y	G	N	K	V	S	I	L	N	Y	L	I	R	N	G	A	D	P	N	K	K	V	R	G	D	P	P	L	F	I	A	A	M	I	G	Q	Y	D	I	V	K
Alpha-Lacrustotoxin-Lt1a Q02989	*L.tredecimguttatus*	[1]	E	N	T	P	I	T	V	A	I	F	A	N	K	V	S	I	L	N	Y	L	V	G	I	G	A	D	P	N	Q	Q	V	D	G	D	P	P	L	Y	I	A	A	R	Q	G	R	F	E	I	V	R
Alpha-Latrocrustotoxin-Sno1a	*S. nobilis*	*This study*	N	G	T	P	I	L	H	A	V	Y	G	N	K	V	N	I	L	N	Y	L	V	P	A	G	A	D	P	N	K	K	V	N	R	D	P	P	L	Y	T	A	A	M	I	G	H	Y	E	I	V	K

IDENTIFICATION	SPECIES	REF	1101	1102	1103	1104	1105	1106	1107	1108	1109	1110	1111	1112	1113	1114	1115	1116	1117	1118	1119	1120	1121	1122	1123	1124	1125	1126	1127	1128	1129	1130	1131	1132	1133	1134	1135	1136	1137	1138	1139	1140	1141	1142	1143	1144	1145	1146	1147	1148	1149	1150
Alpha-Lacrustotoxin-Lt1a Q9XZC0	*L.tredecimguttatus*	[1]	S	L	V	E	Q	H	K	I	D	V	N	T	R	N	K	E	Q	-	F	T	P	L	H	A	A	A	S	N	D	H	I	D	V	V	K	Y	L	I	Q	K	G	A	D	V	N	A	K	G	D	E
Alpha-Lacrustotoxin-Lt1a Q02989	*L.tredecimguttatus*	[1]	C	L	I	E	V	H	K	V	D	I	N	T	R	N	K	E	R	-	F	T	A	L	H	A	A	A	R	N	D	F	M	D	V	V	K	Y	L	V	R	Q	G	A	D	V	N	A	K	G	I	D
Alpha-Latrocrustotoxin-Sno1a	*S. nobilis*	*This study*	S	L	I	E	K	H	G	V	D	V	N	T	R	N	E	E	E	N	F	T	P	L	H	A	A	A	R	N	D	H	I	D	V	V	K	Y	L	I	S	K	G	A	D	V	N	A	V	G	I	D

IDENTIFICATION	SPECIES	REF	1151	1152	1153	1154	1155	1156	1157	1158	1159	1160	1161	1162	1163	1164	1165	1166	1167
Alpha-Lacrustotoxin-Lt1a Q9XZC0	*L.tredecimguttatus*	[1]	N	L	K	P	I	D	L	A	G	E	K	S	K	A	Y	L	R
Alpha-Lacrustotoxin-Lt1a Q02989	*L.tredecimguttatus*	[1]	D	L	R	P	I	D	I	A	G	E	K	A	K	A	Y	L	-
Alpha-Latrocrustotoxin-Sno1a	*S. nobilis*	*This study*	N	L	K	P	I	D	L	A	G	E	K	S	Q	A	Y	L	-

Reference

[1] Grishin E.V. 1998, Toxicon, 36, 1693-1701.

Supplementary dataset 2 – Table 3a- Alpha-Latrotoxins- Sequences from position 1 to 500: Sequence alignments of the closest two alpha-latrotoxins already published and the five most abundant found in the venom of *Steatoda nobilis*. Yellow windows indicate positions where the identity and/or the physico-chemical properties of the aminoacids is maintained at least for 4 possibilities over 7, bold positions are the ones where the positions are maintained 2 or 3 times (over 7). Cysteines are highlighted in red.

IDENTIFICATION	SPECIES	REF	1	2	3	4	5	6	7	8	9	10	11	12	13	14	15	16	17	18	19	20	21	22	23	24	25	26	27	28	29	30	31	32	33	34	35	36	37	38	39	40	41	42	43	44	45	46	47	48	49	50
Alpha-Latrotoxin L7X8P2	S. grossa	/	E	D	V	E	L	S	L	E	E	K	A	E	I	C	S	E	L	E	L	Q	Q	K	S	T	D	I	A	A	N	I	I	G	D	I	S	S	L	P	I	V	G	K	I	V	G	T	M	A	A	A
Alpha-Latrotoxin L7XCU0	L. geometricus	/	-	N	E	E	L	T	L	E	E	K	A	E	I	C	S	E	L	E	L	Q	Q	K	Y	A	D	I	A	S	N	I	I	G	D	L	S	S	L	P	M	V	G	K	I	V	G	T	I	A	A	A
Alpha-Latrotoxin-Sn1a	S. nobilis	This Study	-	-	-	-	-	-	-	-	-	-	-	-	-	-	-	-	-	-	-	-	-	-	-	-	-	-	-	-	-	-	-	-	-	-	-	-	-	-	-	-	-	-	-	-	-	-	-	-	-	-
Alpha-Latrotoxin-Sn1b	S. nobilis	This Study	S	K	R	D	K	A	R	D	E	K	A	A	I	C	K	A	M	E	I	E	S	L	S	I	G	I	A	A	D	V	S	S	A	C	S	G	I	E	I	V	G	P	F	A	A	-	-	-	-	G
Alpha-Latrotoxin-Sn1c	S. nobilis	This Study	S	R	R	E	M	S	L	K	E	R	G	K	L	C	K	D	M	E	L	S	Q	K	S	F	D	M	V	S	N	V	L	G	D	I	N	S	I	P	V	I	G	K	V	A	G	I	V	T	A	G
Alpha-Latrotoxin-Sn1d	S. nobilis	This Study	S	K	R	K	M	S	V	Q	E	R	G	K	L	C	K	D	L	E	L	S	Q	K	S	F	D	M	V	S	N	V	L	G	D	I	N	S	I	P	V	I	G	K	V	A	G	I	V	T	A	G
Alpha-Latrotoxin-Sn1e	S. nobilis	This Study	S	R	R	E	M	S	L	K	E	R	G	K	L	C	K	D	L	E	I	S	Q	K	S	F	D	M	V	S	N	V	L	G	D	I	N	S	I	P	V	I	G	K	V	A	G	I	V	T	A	G

IDENTIFICATION	SPECIES	REF	51	52	53	54	55	56	57	58	59	60	61	62	63	64	65	66	67	68	69	70	71	72	73	74	75	76	77	78	79	80	81	82	83	84	85	86	87	88	89	90	91	92	93	94	95	96	97	98	99	100
Alpha-Latrotoxin L7X8P2	S. grossa	/	M	M	A	I	A	H	V	G	S	G	A	L	D	I	Q	Q	T	L	L	G	C	S	D	L	P	F	D	E	I	K	E	I	M	E	Q	K	F	N	E	I	D	R	K	L	D	S	H	S	K	A
Alpha-Latrotoxin L7XCU0	L. geometricus	/	A	M	T	V	A	H	V	G	S	G	R	L	D	I	E	Q	T	L	L	G	C	S	D	L	P	F	D	Q	I	K	E	I	L	E	T	R	F	N	E	V	D	R	K	L	E	S	H	S	A	A
Alpha-Latrotoxin-Sn1a	S. nobilis	This Study	-	-	-	-	-	-	-	-	-	-	-	-	-	-	-	-	-	-	-	-	C	-	-	-	-	-	-	-	-	-	-	-	-	-	-	-	-	-	-	-	-	-	-	-	-	-	-	-	-	-
Alpha-Latrotoxin-Sn1b	S. nobilis	This Study	A	A	A	V	S	Y	A	F	K	G	G	I	D	I	A	R	V	A	M	D	C	A	D	I	P	F	D	E	I	K	E	I	L	E	T	R	F	N	D	I	D	R	K	L	D	R	N	T	E	A
Alpha-Latrotoxin-Sn1c	S. nobilis	This Study	V	S	V	V	T	H	L	A	A	G	G	L	D	I	A	Q	T	A	L	D	C	A	D	V	P	F	D	D	L	K	E	F	M	Q	K	Q	I	N	E	V	N	R	K	L	D	S	N	S	A	A
Alpha-Latrotoxin-Sn1d	S. nobilis	This Study	V	S	I	A	T	H	M	T	A	G	G	L	D	I	A	M	T	A	L	D	C	G	D	V	P	F	D	D	L	K	E	F	M	Q	K	Q	I	N	E	V	N	R	K	L	D	S	N	S	A	A
Alpha-Latrotoxin-Sn1e	S. nobilis	This Study	V	S	V	V	T	H	L	A	A	G	G	L	D	I	A	Q	T	A	L	D	C	A	D	V	P	F	D	E	L	K	E	F	M	Q	K	Q	I	N	E	V	N	R	K	L	D	S	N	S	A	A

IDENTIFICATION	SPECIES	REF	101	102	103	104	105	106	107	108	109	110	111	112	113	114	115	116	117	118	119	120	121	122	123	124	125	126	127	128	129	130	131	132	133	134	135	136	137	138	139	140	141	142	143	144	145	146	147	148	149	150
Alpha-Latrotoxin L7X8P2	S. grossa	/	L	E	E	I	T	K	L	A	E	K	T	L	S	S	V	E	K	T	R	K	Q	M	N	K	R	F	D	E	V	L	E	S	I	K	N	T	N	I	A	P	I	V	S	K	I	S	N	F	G	R
Alpha-Latrotoxin L7XCU0	L. geometricus	/	L	E	E	I	T	K	L	V	D	K	S	I	S	A	V	E	K	T	R	K	Q	M	N	K	R	F	D	E	V	M	K	S	I	Q	D	A	K	V	S	P	I	V	S	K	I	N	N	F	A	K
Alpha-Latrotoxin-Sn1a	S. nobilis	This Study	-	-	-	-	-	-	-	-	-	-	-	-	-	-	-	-	-	-	-	-	-	-	-	-	-	-	-	-	-	-	-	-	-	-	-	-	-	-	-	-	-	-	-	-	-	-	-	-	-	-
Alpha-Latrotoxin-Sn1b	S. nobilis	This Study	L	R	E	V	R	F	M	V	S	K	T	L	K	G	I	E	N	I	R	I	E	M	K	Q	Q	F	K	K	V	L	D	S	I	R	S	S	R	I	E	P	I	V	D	K	I	N	D	F	V	D
Alpha-Latrotoxin-Sn1c	S. nobilis	This Study	L	E	T	I	S	R	M	V	S	K	T	L	S	A	V	Q	E	S	R	K	E	M	N	E	R	F	D	E	V	L	V	S	I	E	S	T	K	I	E	P	I	V	S	K	I	N	N	F	V	R
Alpha-Latrotoxin-Sn1d	S. nobilis	This Study	L	E	T	I	S	R	M	V	S	K	T	L	S	A	V	Q	E	S	R	K	E	M	N	E	R	F	D	E	V	L	V	S	I	E	S	T	K	I	E	P	I	V	S	K	I	N	N	F	V	R
Alpha-Latrotoxin-Sn1e	S. nobilis	This Study	L	A	E	I	S	R	M	I	N	K	T	L	S	A	V	Q	E	S	R	D	E	M	N	E	R	F	D	E	V	L	E	S	I	E	S	T	K	I	E	P	I	V	S	K	I	N	N	F	V	R

IDENTIFICATION	SPECIES	REF	151	152	153	154	155	156	157	158	159	160	161	162	163	164	165	166	167	168	169	170	171	172	173	174	175	176	177	178	179	180	181	182	183	184	185	186	187	188	189	190	191	192	193	194	195	196	197	198	199	200
Alpha-Latrotoxin L7X8P2	S. grossa	/	Y	F	D	X	E	A	F	K	A	L	D	A	L	L	Y	G	T	Q	T	Y	V	S	V	M	F	F	L	L	E	Q	Y	S	F	L	A	N	Y	Y	Y	G	K	G	E	L	D	I	Y	N	K	Y
Alpha-Latrotoxin L7XCU0	L. geometricus	/	Y	F	D	A	E	A	F	K	V	L	Y	A	I	L	Y	G	T	Q	T	Y	V	S	V	M	F	F	L	L	E	Q	Y	S	F	L	A	N	H	Y	Y	E	K	G	D	L	E	K	Y	D	E	Y
Alpha-Latrotoxin-Sn1a	S. nobilis	This Study	-	-	-	-	-	-	-	-	-	-	-	-	-	-	-	-	-	-	-	-	-	-	-	-	-	-	-	-	-	-	-	-	-	-	-	-	-	-	-	-	-	-	-	-	-	-	-	-	-	-
Alpha-Latrotoxin-Sn1b	S. nobilis	This Study	S	F	D	K	E	A	I	S	A	L	R	A	L	Y	Y	G	T	Q	A	Y	L	E	L	M	Y	F	L	I	E	K	Y	T	Y	L	A	E	Y	H	Y	Q	N	R	M	F	D	L	Y	N	T	Y
Alpha-Latrotoxin-Sn1c	S. nobilis	This Study	Y	F	E	I	E	S	F	K	A	L	Y	A	L	L	Y	G	T	E	A	H	I	S	V	M	S	F	L	I	E	Q	Y	S	Y	L	A	N	Y	H	Y	K	R	G	E	I	E	E	Y	N	E	C
Alpha-Latrotoxin-Sn1d	S. nobilis	This Study	Y	F	E	I	E	S	F	K	A	L	Y	A	L	L	Y	G	T	E	A	H	I	S	V	M	S	F	L	I	E	Q	Y	S	Y	L	A	N	Y	H	Y	K	R	G	E	I	E	E	Y	N	E	C
Alpha-Latrotoxin-Sn1e	S. nobilis	This Study	Y	F	E	S	E	S	F	K	A	L	Y	A	L	L	Y	G	T	E	A	H	V	S	V	M	S	F	L	I	E	Q	Y	S	Y	L	A	N	Y	H	Y	K	R	G	E	I	D	E	Y	N	E	C

IDENTIFICATION	SPECIES	REF	201	202	203	204	205	206	207	208	209	210	211	212	213	214	215	216	217	218	219	220	221	222	223	224	225	226	227	228	229	230	231	232	233	234	235	236	237	238	239	240	241	242	243	244	245	246	247	248	249	250
Alpha-Latrotoxin L7X8P2	S. grossa	/	F	D	S	I	T	N	I	F	L	D	F	K	S	S	L	V	G	S	G	G	S	D	S	A	A	L	L	E	S	V	L	Q	V	L	V	A	V	K	S	K	E	F	L	K	F	L	E	M	D	V
Alpha-Latrotoxin L7XCU0	L. geometricus	/	F	N	N	L	N	N	V	F	L	D	F	K	S	S	L	V	G	S	G	A	S	N	N	E	G	L	I	D	N	V	L	Q	V	L	M	T	V	K	S	N	E	F	L	G	L	G	K	N	S	L
Alpha-Latrotoxin-Sn1a	S. nobilis	This Study	-	-	-	-	-	-	-	-	-	-	-	-	-	-	-	-	-	-	-	-	-	-	-	-	-	-	-	-	-	-	-	-	-	-	-	-	-	-	-	-	-	-	-	-	-	-	-	-	-	-
Alpha-Latrotoxin-Sn1b	S. nobilis	This Study	F	D	S	I	I	T	N	F	N	T	F	Q	T	Y	L	I	G	D	G	K	-	-	-	D	G	L	I	P	K	V	I	K	V	F	Q	T	A	I	D	E	K	L	-	-	-	L	P	T	D	V
Alpha-Latrotoxin-Sn1c	S. nobilis	This Study	F	H	S	I	N	N	I	F	S	E	F	K	S	S	L	L	G	T	G	T	L	K	S	E	S	L	L	D	Q	V	I	Q	T	L	I	E	V	T	K	L	D	F	L	L	F	Q	R	A	D	I
Alpha-Latrotoxin-Sn1d	S. nobilis	This Study	F	H	S	I	N	N	I	F	S	E	F	K	S	S	L	L	G	T	G	T	L	K	S	E	S	L	L	D	Q	V	I	Q	T	L	I	E	V	T	K	L	D	F	L	L	F	Q	R	A	D	I
Alpha-Latrotoxin-Sn1e	S. nobilis	This Study	F	D	S	I	N	N	I	F	L	E	F	K	S	S	L	V	G	T	G	I	A	K	G	E	P	L	L	D	Q	V	I	Q	T	L	I	D	V	S	K	I	D	F	L	V	I	Q	R	H	D	I

IDENTIFICATION	SPECIES	REF	251	252	253	254	255	256	257	258	259	260	261	262	263	264	265	266	267	268	269	270	271	272	273	274	275	276	277	278	279	280	281	282	283	284	285	286	287	288	289	290	291	292	293	294	295	296	297	298	299	300
Alpha-Latrotoxin L7X8P2	S. grossa	/	V	V	M	L	N	E	K	I	S	L	F	S	K	I	K	E	D	V	D	G	K	E	R	I	T	S	S	L	T	P	E	N	Y	V	H	I	S	F	E	K	D	I	P	T	P	F	G	D	W	K
Alpha-Latrotoxin L7XCU0	L. geometricus	/	E	E	M	L	N	E	K	I	N	L	F	S	K	I	K	E	E	I	E	G	K	Q	R	M	T	L	S	E	T	P	E	N	F	A	R	I	S	F	E	K	D	I	T	T	P	I	G	D	W	R
Alpha-Latrotoxin-Sn1a	S. nobilis	This Study	-	-	-	-	-	-	-	-	-	-	-	-	-	-	-	-	-	-	-	-	M	V	L	P	I	[illegible]	I	E	T	P	E	N	Y	V	Q	I	S	F	K	K	D	I	K	T	P	I	G	E	W	E
Alpha-Latrotoxin-Sn1b	S. nobilis	This Study	K	D	Q	V	A	K	Q	I	E	S	L	N	S	I	F	N	E	V	K	N	M	T	L	S	I	I	E	D	R	P	D	N	Y	K	R	I	A	F	Q	L	R	L	P	-	S	H	G	S	W	K
Alpha-Latrotoxin-Sn1c	S. nobilis	This Study	V	Q	M	L	N	N	K	I	E	I	F	K	K	I	K	E	D	I	R	E	M	K	P	T	I	T	A	N	T	P	S	N	Y	A	Q	I	S	F	D	N	K	I	G	A	P	I	G	E	W	E
Alpha-Latrotoxin-Sn1d	S. nobilis	This Study	V	Q	M	L	N	N	K	I	E	I	F	K	K	I	K	E	D	I	R	E	M	K	P	T	I	T	A	N	T	P	S	N	Y	A	Q	I	S	F	D	N	K	I	G	A	P	I	G	E	W	E
Alpha-Latrotoxin-Sn1e	S. nobilis	This Study	V	Q	M	L	K	N	K	I	E	I	F	K	K	I	K	D	D	I	R	D	M	E	P	S	I	T	T	N	T	P	S	N	Y	V	H	I	S	F	D	T	K	I	G	V	P	I	L	E	W	E

IDENTIFICATION	SPECIES	REF	301	302	303	304	305	306	307	308	309	310	311	312	313	314	315	316	317	318	319	320	321	322	323	324	325	326	327	328	329	330	331	332	333	334	335	336	337	338	339	340	341	342	343	344	345	346	347	348	349	350
Alpha-Latrotoxin L7X8P2	S. grossa	/	E	D	K	E	V	R	Y	A	V	Q	Y	E	S	E	T	L	F	S	K	V	S	H	W	S	N	P	I	T	V	R	G	K	A	C	P	T	L	R	-	M	P	V	D	Q	N	R	K	S	I	L
Alpha-Latrotoxin L7XCU0	L. geometricus	/	D	S	R	E	V	R	Y	A	V	Q	Y	A	S	E	T	L	F	S	K	I	G	H	W	S	D	P	V	S	V	R	A	K	A	C	P	T	L	R	-	M	P	V	D	Q	T	R	R	N	V	L
Alpha-Latrotoxin-Sn1a	S. nobilis	This Study	N	G	K	D	V	R	Y	A	V	Q	Y	E	S	E	N	A	Y	S	N	V	G	D	W	S	K	A	T	I	V	R	A	K	A	C	P	S	L	R	-	I	P	V	D	Q	R	R	K	T	R	L
Alpha-Latrotoxin-Sn1b	S. nobilis	This Study	E	G	K	K	V	R	Y	A	V	Q	F	E	K	K	G	R	H	S	K	I	G	P	W	S	D	F	F	S	L	N	G	E	A	N	P	T	I	E	A	L	P	P	A	K	D	G	S	K	R	L
Alpha-Latrotoxin-Sn1c	S. nobilis	This Study	K	H	K	K	V	Q	Y	A	V	Q	Y	R	N	K	T	S	F	S	G	V	G	E	W	S	E	P	L	T	I	N	N	Y	A	C	P	I	L	R	-	M	G	N	D	P	T	R	N	T	V	L
Alpha-Latrotoxin-Sn1d	S. nobilis	This Study	K	H	K	K	V	Q	Y	A	V	Q	Y	R	N	K	T	S	F	S	G	V	G	E	W	S	E	P	L	T	I	N	N	Y	A	C	P	I	L	R	-	M	G	N	D	P	T	R	N	T	V	L
Alpha-Latrotoxin-Sn1e	S. nobilis	This Study	K	H	K	R	V	Q	Y	A	V	Q	Y	K	N	R	T	S	F	S	R	V	G	E	W	S	E	L	L	T	I	N	N	Y	A	C	P	I	L	R	-	M	A	N	D	P	S	R	N	T	V	L

IDENTIFICATION	SPECIES	REF	351	352	353	354	355	356	357	358	359	360	361	362	363	364	365	366	367	368	369	370	371	372	373	374	375	376	377	378	379	380	381	382	383	384	385	386	387	388	389	390	391	392	393	394	395	396	397	398	399	400
Alpha-Latrotoxin L7X8P2	S. grossa	/	V	F	R	K	F	E	N	R	K	P	Q	L	V	G	S	V	S	P	Y	Q	S	N	F	I	D	I	D	R	D	L	Y	N	A	A	G	N	P	D	L	D	V	G	H	X	E	V	V	K	L	V
Alpha-Latrotoxin L7XCU0	L. geometricus	/	V	F	R	K	F	D	N	S	K	P	Q	L	V	G	E	I	T	P	Y	Q	S	N	F	I	D	I	D	R	D	L	Y	N	A	A	S	N	P	D	S	A	V	G	F	M	E	F	K	K	L	I
Alpha-Latrotoxin-Sn1a	S. nobilis	This Study	I	F	R	K	F	D	N	D	Q	P	Q	L	A	G	R	V	K	P	Y	E	T	L	F	T	D	I	N	R	D	V	Y	N	A	A	E	M	S	D	R	D	T	G	L	A	E	A	K	I	F	L
Alpha-Latrotoxin-Sn1b	S. nobilis	This Study	I	F	R	Q	I	D	D	G	N	P	Q	F	V	G	K	I	S	S	G	K	N	K	F	I	D	I	S	R	D	I	Y	N	A	A	G	N	I	H	A	N	I	A	L	P	E	M	E	L	L	L
Alpha-Latrotoxin-Sn1c	S. nobilis	This Study	I	F	R	K	F	Q	G	S	K	P	Q	L	V	G	S	V	E	P	Y	Q	Q	E	F	T	D	I	H	R	D	L	Y	D	A	A	E	Y	P	E	K	E	T	G	F	E	T	A	K	I	L	L
Alpha-Latrotoxin-Sn1d	S. nobilis	This Study	I	F	R	K	F	Q	G	S	K	P	Q	L	V	G	S	V	E	P	Y	Q	Q	E	F	L	D	I	H	R	D	L	Y	N	A	A	E	Y	A	E	K	E	T	G	F	K	T	A	E	I	L	L
Alpha-Latrotoxin-Sn1e	S. nobilis	This Study	I	F	R	K	F	H	G	S	K	P	Q	L	V	G	S	V	E	P	Y	Q	Q	E	F	L	D	I	H	R	D	L	Y	N	A	A	E	Y	A	E	K	E	T	G	F	K	T	A	E	I	L	L

IDENTIFICATION	SPECIES	REF	401	402	403	404	405	406	407	408	409	410	411	412	413	414	415	416	417	418	419	420	421	422	423	424	425	426	427	428	429	430	431	432	433	434	435	436	437	438	439	440	441	442	443	444	445	446	447	448	449	450
Alpha-Latrotoxin L7X8P2	S. grossa	/	A	-	N	G	A	N	I	K	A	P	F	E	Q	G	R	T	A	L	H	A	A	A	E	S	G	N	A	K	V	M	F	G	L	V	L	-	X	S	L	G	D	L	N	K	P	D	K	N	G	F
Alpha-Latrotoxin L7XCU0	L. geometricus	/	S	-	N	G	A	N	I	R	A	V	F	N	Q	G	R	T	V	F	H	A	A	A	K	S	G	N	D	K	I	M	I	E	L	T	F	F	S	K	Y	T	D	I	N	Q	P	D	K	K	G	Y
Alpha-Latrotoxin-Sn1a	S. nobilis	This Study	S	-	N	G	G	D	I	N	T	A	F	E	K	G	R	R	A	F	H	A	V	A	Y	S	G	N	F	R	L	A	T	Y	F	L	L	V	D	N	S	T	D	I	N	V	R	D	K	N	G	Y
Alpha-Latrotoxin-Sn1b	S. nobilis	This Study	K	Y	K	E	A	N	I	D	E	K	F	D	D	G	R	R	A	I	H	A	A	A	K	S	D	N	V	D	L	A	K	R	F	I	I	D	P	K	K	T	D	V	N	S	Q	D	D	M	G	Y
Alpha-Latrotoxin-Sn1c	S. nobilis	This Study	K	-	K	G	A	N	F	T	A	V	F	E	K	G	R	T	V	L	H	A	A	A	K	S	G	N	A	E	I	A	L	G	I	L	L	R	G	N	P	K	E	I	N	V	A	D	E	D	G	Y
Alpha-Latrotoxin-Sn1d	S. nobilis	This Study	K	-	K	G	A	N	F	T	A	T	F	E	K	D	R	T	V	L	H	A	A	A	K	S	G	N	T	E	I	A	L	G	V	L	L	R	G	N	T	K	D	I	N	I	A	D	E	D	G	Y
Alpha-Latrotoxin-Sn1e	S. nobilis	This Study	K	-	K	G	A	N	F	T	A	T	F	E	K	D	R	T	V	L	H	A	A	A	K	S	G	N	T	E	I	A	L	G	V	L	L	R	G	N	T	K	D	I	N	I	A	D	E	D	G	Y

IDENTIFICATION	SPECIES	REF	451	452	453	454	455	456	457	458	459	460	461	462	463	464	465	466	467	468	469	470	471	472	473	474	475	476	477	478	479	480	481	482	483	484	485	486	487	488	489	490	491	492	493	494	495	496	497	498	499	500
Alpha-Latrotoxin L7X8P2	S. grossa	/	T	A	I	H	A	A	A	D	K	G	N	A	G	I	V	N	L	L	I	D	Y	S	A	S	V	N	V	K	T	Y	R	T	L	Q	T	P	L	H	L	A	A	R	R	G	F	K	R	T	F	D
Alpha-Latrotoxin L7XCU0	L. geometricus	/	T	P	I	H	V	A	A	D	S	G	N	A	G	I	V	N	L	L	I	R	S	G	I	S	V	N	S	K	T	Y	N	F	L	Q	T	P	L	H	L	A	A	Q	R	G	F	V	A	T	F	Q
Alpha-Latrotoxin-Sn1a	S. nobilis	This Study	T	P	L	H	V	A	A	S	V	G	Q	S	G	F	V	K	F	L	L	N	H	E	A	L	V	N	A	K	T	D	P	E	L	S	T	P	L	H	L	A	A	R	L	G	F	V	K	S	I	S
Alpha-Latrotoxin-Sn1b	S. nobilis	This Study	A	P	I	H	V	S	V	E	V	K	S	K	N	F	T	L	M	L	L	N	N	G	A	N	I	N	I	E	T	N	L	G	-	Y	T	P	L	H	L	A	V	K	L	G	Y	K	G	I	I	R
Alpha-Latrotoxin-Sn1c	S. nobilis	This Study	T	P	M	H	I	A	A	D	S	G	N	A	G	F	V	K	F	L	I	N	H	D	G	L	V	N	V	K	T	N	R	G	H	L	T	P	L	H	F	A	A	R	R	G	F	L	K	S	F	N
Alpha-Latrotoxin-Sn1d	S. nobilis	This Study	T	P	M	H	I	A	A	N	S	G	N	A	G	F	V	K	F	L	I	N	H	D	A	S	V	N	V	K	T	N	R	R	L	L	T	P	L	H	L	A	A	R	R	G	F	L	K	T	F	N
Alpha-Latrotoxin-Sn1e	S. nobilis	This Study	T	P	M	H	I	A	A	N	S	G	N	A	G	F	V	K	F	L	I	N	H	D	A	S	V	N	V	K	T	N	R	R	L	L	T	P	L	H	L	A	A	R	R	G	F	L	K	T	F	N

Supplementary dataset 2 – Table 3b- Alpha-Latrotoxins- Sequences from position 501 to 950: Sequence alignments of the closest two alpha-latrotoxins already published and the five most abundant found in the venom of *Steatoda nobilis*. Yellow windows indicate positions where the identity and/or the physico-chemical properties of the aminoacids is maintained at least for 4 possibilities over 7, bold positions are the ones where the positions are maintained 2 or 3 times (over 7). Cysteines are highlighted in red.

IDENTIFICATION	SPECIES	REF	501	502	503	504	505	506	507	508	509	510	511	512	513	514	515	516	517	518	519	520	521	522	523	524	525	526	527	528	529	530	531	532	533	534	535	536	537	538	539	540	541	542	543	544	545	546	547	548	549	550
Alpha-Latrotoxin L7X8P2	S. grossa	/	R	L	L	E	S	P	D	I	N	I	N	E	K	D	K	D	G	F	T	P	L	H	S	A	V	L	G	G	K	E	I	V	K	A	F	I	N	Q	P	G	T	D	I	N	A	K	S	E	-	K
Alpha-Latrotoxin L7XCU0	L. geometricus	/	R	L	M	E	S	P	E	I	N	I	N	E	R	D	K	D	G	F	T	P	L	H	Y	A	V	R	G	G	E	R	I	L	E	A	F	I	N	Q	A	G	I	D	V	N	V	K	S	D	-	K
Alpha-Latrotoxin-Sn1a	S. nobilis	This Study	T	L	L	A	S	P	K	I	N	I	N	E	R	N	R	S	G	F	T	A	L	H	N	A	V	L	G	G	R	Y	V	L	S	A	L	I	S	H	D	R	I	D	I	N	A	K	S	K	-	T
Alpha-Latrotoxin-Sn1b	S. nobilis	This Study	S	L	I	E	R	P	G	I	E	L	N	K	K	D	K	N	G	F	T	P	L	H	F	A	V	A	H	N	H	K	V	V	K	L	L	V	S	K	T	D	T	K	V	N	E	K	S	N	-	L
Alpha-Latrotoxin-Sn1c	S. nobilis	This Study	R	L	L	E	S	P	E	I	N	I	N	E	R	N	E	E	G	F	T	P	L	H	Y	A	V	L	G	G	K	F	I	V	M	E	F	I	K	R	K	E	T	E	I	N	P	K	S	A	K	K
Alpha-Latrotoxin-Sn1d	S. nobilis	This Study	R	L	L	E	S	P	D	I	K	I	N	E	R	N	E	E	G	F	T	P	L	H	Y	A	V	L	G	G	K	L	M	I	T	V	F	M	K	N	S	K	T	E	I	N	P	R	S	T	K	K
Alpha-Latrotoxin-Sn1e	S. nobilis	This Study	R	L	L	E	S	P	D	I	K	I	N	E	R	N	E	E	G	F	T	P	L	H	Y	A	V	L	G	G	K	L	M	I	T	V	F	M	K	N	S	K	T	E	I	N	P	R	S	T	K	K

IDENTIFICATION	SPECIES	REF	551	552	553	554	555	556	557	558	559	560	561	562	563	564	565	566	567	568	569	570	571	572	573	574	575	576	577	578	579	580	581	582	583	584	585	586	587	588	589	590	591	592	593	594	595	596	597	598	599	600
Alpha-Latrotoxin L7X8P2	S. grossa	/	G	L	T	P	F	H	L	A	I	I	N	D	D	W	P	-	-	-	-	V	A	E	T	L	L	T	S	E	S	L	Q	V	N	A	N	D	E	N	N	N	T	P	L	H	Y	A	V	M	L	G
Alpha-Latrotoxin L7XCU0	L. geometricus	/	G	L	T	P	F	H	L	A	V	I	K	N	D	W	P	-	-	-	-	V	A	S	T	L	L	R	S	K	K	I	D	I	N	A	I	D	E	N	N	M	T	A	L	H	Y	A	A	I	L	G
Alpha-Latrotoxin-Sn1a	S. nobilis	This Study	G	L	T	P	F	H	L	A	A	I	N	N	D	R	D	-	-	-	-	V	A	E	V	L	I	G	S	S	K	V	D	V	N	A	G	D	S	N	S	M	T	P	L	H	Y	A	A	M	L	G
Alpha-Latrotoxin-Sn1b	S. nobilis	This Study	H	L	T	P	F	H	I	A	V	M	K	D	V	N	Q	E	F	N	K	T	L	M	A	L	L	K	S	G	R	V	N	I	N	E	G	N	D	N	N	M	T	A	L	H	F	A	C	F	G	D
Alpha-Latrotoxin-Sn1c	S. nobilis	This Study	E	L	T	P	F	H	M	A	V	I	N	D	D	I	P	-	-	-	-	V	A	Q	I	L	I	Q	S	Q	K	V	E	V	N	A	G	D	K	D	S	M	T	S	L	H	Y	A	A	M	L	G
Alpha-Latrotoxin-Sn1d	S. nobilis	This Study	E	L	T	P	F	H	M	A	V	I	N	N	D	L	P	-	-	-	-	V	V	Q	T	L	M	E	S	R	K	I	E	I	N	A	G	D	K	N	N	M	A	P	L	H	Y	S	A	M	L	G
Alpha-Latrotoxin-Sn1e	S. nobilis	This Study	E	L	T	P	F	H	M	A	V	I	N	N	D	L	P	-	-	-	-	V	V	Q	T	L	M	E	S	R	K	I	E	I	N	A	G	D	K	N	N	M	A	P	L	H	Y	S	A	M	L	G

IDENTIFICATION	SPECIES	REF	601	602	603	604	605	606	607	608	609	610	611	612	613	614	615	616	617	618	619	620	621	622	623	624	625	626	627	628	629	630	631	632	633	634	635	636	637	638	639	640	641	642	643	644	645	646	647	648	649	650
Alpha-Latrotoxin L7X8P2	S. grossa	/	H	-	L	E	I	M	K	K	L	I	N	L	S	Q	V	N	V	N	A	V	S	I	P	N	L	W	S	A	L	H	Y	A	I	F	F	K	R	D	D	V	T	L	E	L	L	K	I	L	K	V
Alpha-Latrotoxin L7XCU0	L. geometricus	/	F	-	L	E	T	T	K	Q	L	I	N	L	K	E	I	N	A	N	A	V	S	S	P	G	L	L	S	A	L	H	Y	A	I	L	Y	K	H	D	D	V	A	L	F	L	L	K	S	S	K	V
Alpha-Latrotoxin-Sn1a	S. nobilis	This Study	N	-	A	E	M	V	H	Y	L	M	T	L	S	G	I	D	I	N	A	V	T	H	D	N	K	W	S	P	L	H	Y	A	I	M	F	K	K	D	N	V	A	L	E	L	L	N	T	G	E	I
Alpha-Latrotoxin-Sn1b	S. nobilis	This Study	N	S	V	A	K	I	Q	F	L	L	N	L	K	D	I	N	V	N	A	K	T	I	D	N	N	W	T	P	L	H	L	A	L	T	L	R	K	D	E	V	V	F	E	L	L	N	A	A	D	I
Alpha-Latrotoxin-Sn1c	S. nobilis	This Study	H	-	S	K	M	T	Q	Y	L	L	S	L	D	R	I	N	P	N	M	A	T	T	D	S	K	W	S	P	L	H	Y	A	I	F	F	K	H	D	D	I	A	M	Q	L	L	N	F	P	N	I
Alpha-Latrotoxin-Sn1d	S. nobilis	This Study	H	-	S	K	M	V	E	Y	L	L	T	L	N	G	I	K	L	N	L	I	T	N	D	K	K	W	S	P	L	H	Y	S	I	Y	F	K	H	D	E	I	A	L	M	L	L	N	S	P	N	I
Alpha-Latrotoxin-Sn1e	S. nobilis	This Study	H	-	S	K	M	V	E	Y	L	L	T	L	N	G	I	K	L	N	L	I	T	N	D	K	K	W	S	P	L	H	Y	S	I	Y	F	K	H	D	E	I	A	L	M	L	L	N	S	P	N	I

IDENTIFICATION	SPECIES	REF	651	652	653	654	655	656	657	658	659	660	661	662	663	664	665	666	667	668	669	670	671	672	673	674	675	676	677	678	679	680	681	682	683	684	685	686	687	688	689	690	691	692	693	694	695	696	697	698	699	700
Alpha-Latrotoxin L7X8P2	S. grossa	/	D	K	D	L	Q	S	L	G	G	I	T	P	L	H	L	A	V	S	L	G	R	K	T	V	V	S	E	M	V	R	I	G	I	N	L	E	Q	K	T	T	E	D	F	T	P	L	H	L	A	A
Alpha-Latrotoxin L7XCU0	L. geometricus	/	N	Y	N	L	K	A	F	G	D	I	T	P	L	H	L	A	V	M	Q	G	R	K	Q	V	L	S	E	M	F	N	I	G	I	N	I	N	Q	K	T	A	E	K	Y	T	P	L	H	L	A	A
Alpha-Latrotoxin-Sn1a	S. nobilis	This Study	N	K	A	L	K	A	D	G	D	I	T	P	L	H	F	A	V	T	F	G	R	K	N	V	V	Q	E	L	I	A	K	K	V	N	T	E	E	K	T	A	E	G	Y	T	A	L	H	L	A	S
Alpha-Latrotoxin-Sn1b	S. nobilis	This Study	N	V	D	L	P	D	S	K	G	V	T	P	L	A	A	A	V	A	S	G	S	S	G	V	V	K	A	L	I	K	K	N	A	N	T	K	T	V	T	K	E	G	Y	T	L	L	H	A	A	A
Alpha-Latrotoxin-Sn1c	S. nobilis	This Study	D	D	S	L	K	A	D	G	D	N	T	P	L	H	F	A	V	V	F	E	R	K	N	V	L	F	E	L	L	K	R	K	V	N	L	E	V	K	T	T	E	G	Y	T	P	L	H	L	A	A
Alpha-Latrotoxin-Sn1d	S. nobilis	This Study	D	D	S	L	K	A	D	S	D	S	T	P	L	H	F	A	V	I	F	E	R	K	N	V	L	S	K	L	L	T	R	N	V	N	L	E	A	K	T	K	E	G	Y	T	P	L	H	L	A	A
Alpha-Latrotoxin-Sn1e	S. nobilis	This Study	D	D	S	L	K	A	D	S	D	S	T	P	L	H	F	A	V	I	F	E	R	K	N	V	L	S	K	L	L	T	R	N	V	N	L	E	A	K	T	K	E	G	Y	T	P	L	H	L	A	A

IDENTIFICATION	SPECIES	REF	701	702	703	704	705	706	707	708	709	710	711	712	713	714	715	716	717	718	719	720	721	722	723	724	725	726	727	728	729	730	731	732	733	734	735	736	737	738	739	740	741	742	743	744	745	746	747	748	749	750
Alpha-Latrotoxin L7X8P2	S. grossa	/	M	S	K	Y	P	E	I	V	T	F	L	I	D	Q	G	S	N	M	E	A	K	T	A	N	G	A	T	P	L	L	L	A	T	L	K	G	R	E	Q	A	S	M	I	L	L	G	N	E	V	N
Alpha-Latrotoxin L7XCU0	L. geometricus	/	M	S	K	Y	P	E	L	V	E	I	L	L	D	Q	G	S	N	L	E	A	K	T	I	T	G	A	T	P	L	N	L	A	T	F	K	G	K	S	Q	A	A	L	I	L	L	K	D	E	V	N
Alpha-Latrotoxin-Sn1a	S. nobilis	This Study	M	R	K	I	P	D	L	I	N	I	L	L	D	S	G	A	N	I	E	A	Q	T	L	N	S	S	T	P	L	H	V	A	S	K	N	G	R	V	E	C	A	V	T	L	L	D	H	K	A	N
Alpha-Latrotoxin-Sn1b	S. nobilis	This Study	L	R	K	E	R	E	L	I	A	S	L	P	I	N	D	F	D	V	N	G	K	T	T	D	G	S	T	P	L	H	L	A	I	S	R	N	N	T	E	P	A	K	F	L	L	E	N	N	A	D
Alpha-Latrotoxin-Sn1c	S. nobilis	This Study	M	R	E	D	P	E	M	L	S	V	L	L	N	N	R	A	N	I	E	A	K	T	I	N	E	S	T	P	L	H	L	A	T	F	T	G	R	S	K	T	A	M	A	L	V	D	S	G	A	N
Alpha-Latrotoxin-Sn1d	S. nobilis	This Study	M	R	E	D	P	E	M	L	S	V	L	L	N	N	R	A	N	I	E	A	K	T	I	N	E	S	T	P	L	H	L	A	T	F	T	G	R	S	K	T	A	L	T	L	I	D	R	G	A	S
Alpha-Latrotoxin-Sn1e	S. nobilis	This Study	M	R	E	D	P	E	M	L	S	V	L	L	N	N	R	A	N	I	E	A	K	T	I	N	E	S	T	P	L	H	L	A	T	F	T	G	R	S	K	T	A	L	T	L	I	D	R	G	A	S

IDENTIFICATION	SPECIES	REF	751	752	753	754	755	756	757	758	759	760	761	762	763	764	765	766	767	768	769	770	771	772	773	774	775	776	777	778	779	780	781	782	783	784	785	786	787	788	789	790	791	792	793	794	795	796	797	798	799	800
Alpha-Latrotoxin L7X8P2	S. grossa	/	F	R	A	V	D	K	D	G	Q	M	P	I	H	G	A	A	M	N	G	M	L	E	I	A	R	E	I	I	N	L	E	P	S	I	V	A	A	Q	N	N	N	L	D	T	P	L	N	L	A	A
Alpha-Latrotoxin L7XCU0	L. geometricus	/	W	R	E	P	D	E	N	G	Q	M	P	I	H	G	A	A	M	N	G	L	L	D	V	A	Q	A	I	L	Y	L	D	A	T	V	L	D	I	E	D	K	N	L	D	T	P	L	N	L	A	A
Alpha-Latrotoxin-Sn1a	S. nobilis	This Study	M	K	A	S	D	I	D	G	Q	V	P	I	H	V	A	A	F	N	D	Q	L	E	M	V	R	A	F	L	A	K	D	A	I	L	M	E	F	K	N	R	D	G	E	T	P	L	L	V	A	A
Alpha-Latrotoxin-Sn1b	S. nobilis	This Study	V	T	I	W	N	K	K	D	F	T	P	M	Q	L	A	A	L	N	D	N	T	E	I	L	R	C	L	L	D	H	K	A	D	I	-	N	T	E	T	H	G	K	M	T	A	F	H	F	A	A
Alpha-Latrotoxin-Sn1c	S. nobilis	This Study	L	K	V	S	D	K	N	N	K	T	P	I	H	G	A	A	V	N	G	Q	L	A	V	I	Q	K	L	L	T	R	D	P	S	I	I	D	I	K	N	K	N	L	D	T	P	L	N	L	A	A
Alpha-Latrotoxin-Sn1d	S. nobilis	This Study	L	K	V	S	D	K	D	N	Q	T	P	L	H	G	A	A	I	R	G	Q	L	E	V	I	Q	K	L	L	T	R	D	P	S	V	I	D	L	K	N	K	N	F	D	T	P	L	N	L	A	A
Alpha-Latrotoxin-Sn1e	S. nobilis	This Study	L	K	V	S	D	K	D	N	Q	T	P	L	H	G	A	A	I	R	G	Q	L	E	V	I	Q	K	L	L	T	R	D	P	S	V	I	D	L	K	N	K	N	F	D	T	P	L	N	L	A	A

IDENTIFICATION	SPECIES	REF	801	802	803	804	805	806	807	808	809	810	811	812	813	814	815	816	817	818	819	820	821	822	823	824	825	826	827	828	829	830	831	832	833	834	835	836	837	838	839	840	841	842	843	844	845	846	847	848	849	850
Alpha-Latrotoxin L7X8P2	S. grossa	/	Q	H	L	H	P	E	M	V	K	F	L	V	G	R	K	A	D	I	N	T	K	N	K	D	G	N	A	P	L	L	A	F	S	K	M	G	N	L	D	M	I	K	F	L	I	S	N	G	A	D
Alpha-Latrotoxin L7XCU0	L. geometricus	/	Q	N	S	H	I	D	M	V	K	Y	F	I	D	L	G	A	K	V	N	T	R	N	K	K	G	Q	A	P	L	L	A	F	S	K	K	G	N	L	D	M	V	K	Y	L	F	D	K	N	A	N
Alpha-Latrotoxin-Sn1a	S. nobilis	This Study	E	K	S	N	T	A	I	V	K	D	L	L	D	K	G	A	D	I	N	S	Q	D	N	V	G	I	A	A	L	H	I	F	A	K	K	G	N	L	S	M	V	K	Y	F	F	D	K	G	A	N
Alpha-Latrotoxin-Sn1b	S. nobilis	This Study	S	S	G	S	V	N	A	L	K	F	L	Q	S	N	G	A	K	V	D	A	K	A	N	G	G	M	T	A	L	H	L	A	A	S	E	G	K	L	D	A	I	L	Y	L	I	E	N	R	A	D
Alpha-Latrotoxin-Sn1c	S. nobilis	This Study	Q	N	F	H	T	A	T	S	K	Y	L	I	D	T	G	A	D	I	N	T	I	N	L	D	G	I	S	P	L	I	V	F	S	K	I	G	S	L	N	M	V	K	Y	L	I	D	K	G	A	D
Alpha-Latrotoxin-Sn1d	S. nobilis	This Study	Q	N	F	H	T	A	T	A	K	Y	L	I	D	K	R	A	D	V	N	A	R	N	L	D	G	I	S	P	L	I	V	F	A	K	F	G	S	L	N	M	V	K	Y	L	I	D	K	G	A	D
Alpha-Latrotoxin-Sn1e	S. nobilis	This Study	Q	N	F	H	T	A	T	A	K	Y	L	I	D	K	R	A	D	V	N	A	R	N	L	D	G	I	S	P	L	I	V	F	A	K	F	G	S	L	N	M	V	K	Y	L	I	D	K	G	A	D

IDENTIFICATION	SPECIES	REF	851	852	853	854	855	856	857	858	859	860	861	862	863	864	865	866	867	868	869	870	871	872	873	874	875	876	877	878	879	880	881	882	883	884	885	886	887	888	889	890	891	892	893	894	895	896	897	898	899	900
Alpha-Latrotoxin L7X8P2	S. grossa	/	V	Y	I	A	D	N	D	G	L	N	F	F	Y	Y	A	V	R	H	G	H	L	N	I	I	K	Y	A	M	S	E	-	R	K	F	T	W	S	T	P	D	N	N	R	R	R	E	C	P	N	-
Alpha-Latrotoxin L7XCU0	L. geometricus	/	V	Y	I	A	D	N	D	G	L	N	F	F	Y	Y	A	V	R	N	G	H	L	N	I	V	K	Y	A	M	S	E	K	D	K	F	E	W	S	T	T	D	N	N	R	S	D	G	C	S	G	-
Alpha-Latrotoxin-Sn1a	S. nobilis	This Study	L	Y	V	S	D	N	Q	G	L	N	F	F	Y	F	A	I	Q	N	D	H	L	N	V	I	K	Y	A	I	S	D	-	K	K	F	N	W	D	T	Q	D	S	K	R	Q	Y	C	M	A	V	-
Alpha-Latrotoxin-Sn1b	S. nobilis	This Study	V	N	I	V	D	D	E	R	N	I	Y	F	Y	H	A	V	R	K	G	H	L	S	V	V	E	H	L	I	S	N	-	K	R	I	K	I	N	S	L	D	N	N	K	N	A	G	Q	V	I	T
Alpha-Latrotoxin-Sn1c	S. nobilis	This Study	V	Y	V	A	D	N	N	G	I	N	F	I	Y	H	A	I	R	N	N	H	L	N	I	V	R	Y	A	N	M	L	K	E	R	I	K	W	N	K	I	D	D	K	R	K	K	E	C	S	I	T
Alpha-Latrotoxin-Sn1d	S. nobilis	This Study	V	Y	V	A	D	N	N	G	I	N	F	I	Y	H	A	I	R	N	N	H	L	N	I	V	R	Y	A	N	M	L	K	E	R	I	K	W	N	K	I	D	D	K	R	K	K	E	C	S	I	T
Alpha-Latrotoxin-Sn1e	S. nobilis	This Study	V	Y	V	A	D	N	N	G	I	N	F	I	Y	H	A	I	R	N	N	H	L	N	I	V	R	Y	A	N	M	L	K	E	R	I	K	W	N	K	I	D	D	K	R	K	K	E	C	S	I	T

IDENTIFICATION	SPECIES	REF	901	902	903	904	905	906	907	908	909	910	911	912	913	914	915	916	917	918	919	920	921	922	923	924	925	926	927	928	929	930	931	932	933	934	935	936	937	938	939	940	941	942	943	944	945	946	947	948	949	950
Alpha-Latrotoxin L7X8P2	S. grossa	/	-	-	-	-	-	E	E	C	A	I	S	H	F	A	V	C	D	A	I	Q	Y	G	K	I	N	V	V	K	Y	F	V	G	T	L	Q	H	F	S	I	C	R	P	L	I	T	A	A	M	Y	G
Alpha-Latrotoxin L7XCU0	L. geometricus	/	-	-	-	-	-	-	E	C	A	I	S	H	F	A	V	C	D	A	V	Q	Y	D	K	I	E	I	V	K	Y	F	V	G	T	L	G	H	Y	S	I	C	S	P	L	H	Q	A	A	R	Y	G
Alpha-Latrotoxin-Sn1a	S. nobilis	This Study	-	-	-	-	-	-	R	C	S	I	K	Y	F	A	L	C	D	A	A	K	F	G	R	L	D	I	L	K	Y	F	I	G	T	L	N	A	-	N	L	C	K	P	L	E	V	G	A	R	E	G
Alpha-Latrotoxin-Sn1b	S. nobilis	This Study	L	-	-	-	P	I	R	K	P	I	I	Y	W	A	M	R	D	A	I	K	Y	G	K	L	D	M	V	E	Y	F	V	G	T	L	G	Y	-	K	V	C	N	P	L	Y	N	A	A	F	F	G
Alpha-Latrotoxin-Sn1c	S. nobilis	This Study	T	K	K	T	P	N	V	C	S	I	E	Y	F	A	V	C	D	S	V	K	Y	G	K	L	D	I	V	K	Y	L	V	G	E	L	G	K	-	N	L	C	Q	P	L	I	E	A	V	N	F	G
Alpha-Latrotoxin-Sn1d	S. nobilis	This Study	T	K	K	T	P	N	V	C	S	I	E	Y	F	A	V	C	D	S	V	K	Y	G	K	L	D	I	V	K	Y	L	V	G	E	L	G	K	-	N	L	C	Q	P	L	I	E	A	V	N	F	G
Alpha-Latrotoxin-Sn1e	S. nobilis	This Study	T	K	K	T	P	N	V	C	S	I	E	Y	F	A	V	C	D	S	V	K	Y	G	K	L	D	I	V	K	Y	L	V	G	E	L	G	K	-	N	L	C	Q	P	L	I	E	A	V	N	F	G

Supplementary dataset 2 – Table 3c- Alpha-Latrotoxins- Sequences from position 951 to 1398: Sequence alignments of the closest two alpha-latrotoxins already published and the five most abundant found in the venom of *Steatoda nobilis*. Yellow windows indicate positions where the identity and/or the physico-chemical properties of the aminoacids is maintained at least for 4 possibilities over 7, bold positions are the ones where the positions are maintained 2 or 3 times (over 7). Cysteines are highlighted in red.

IDENTIFICATION	SPECIES	REF	951	952	953	954	955	956	957	958	959	960	961	962	963	964	965	966	967	968	969	970	971	972	973	974	975	976	977	978	979	980	981	982	983	984	985	986	987	988	989	990	991	992	993	994	995	996	997	998	999	1000
Alpha-Latrotoxin L7X8P2	S. grossa	/	Q	L	D	I	V	K	Y	L	V	E	E	E	E	L	N	V	N	V	A	-	-	-	-	-	K	P	G	T	P	L	C	H	A	A	E	N	G	Y	L	D	V	V	K	Y	L	V	E	N	G	X
Alpha-Latrotoxin L7XCU0	L. geometricus	/	H	I	H	I	V	K	Y	L	V	E	E	E	V	L	S	V	D	G	S	-	-	-	-	-	K	P	D	T	P	L	C	Y	A	S	E	N	G	H	L	A	V	V	Q	Y	L	I	R	N	G	A
Alpha-Latrotoxin-Sn1a	S. nobilis	This Study	H	L	E	I	V	K	Y	M	V	E	D	R	K	Q	K	P	D	G	P	R	-	R	A	V	V	I	N	S	P	M	C	D	A	A	F	R	G	H	L	E	I	V	K	Y	L	K	E	K	K	A
Alpha-Latrotoxin-Sn1b	S. nobilis	This Study	E	Y	K	I	V	K	Y	L	V	E	E	V	F	L	D	V	D	T	S	Y	D	D	D	R	P	N	D	V	P	L	L	G	A	I	F	G	S	S	L	K	I	V	K	Y	L	V	G	K	G	A
Alpha-Latrotoxin-Sn1c	S. nobilis	This Study	W	L	G	I	V	K	Y	L	V	E	E	I	H	M	D	V	N	P	Y	K	D	W	Y	N	E	F	N	T	P	L	C	T	A	A	K	R	G	H	L	E	I	V	H	F	L	V	Q	E	K	A
Alpha-Latrotoxin-Sn1d	S. nobilis	This Study	W	L	G	I	V	K	Y	L	V	E	E	I	H	M	D	V	N	P	Y	K	D	W	Y	N	E	F	N	T	P	L	C	T	A	A	K	R	G	H	L	E	I	V	H	F	L	V	Q	E	K	A
Alpha-Latrotoxin-Sn1e	S. nobilis	This Study	W	L	G	I	V	K	Y	L	V	E	E	I	H	M	D	V	N	P	Y	K	D	W	Y	N	E	F	N	T	P	L	C	T	A	A	K	R	G	H	L	E	I	V	H	F	L	V	Q	E	K	A

IDENTIFICATION	SPECIES	REF	1001	1002	1003	1004	1005	1006	1007	1008	1009	1010	1011	1012	1013	1014	1015	1016	1017	1018	1019	1020	1021	1022	1023	1024	1025	1026	1027	1028	1029	1030	1031	1032	1033	1034	1035	1036	1037	1038	1039	1040	1041	1042	1043	1044	1045	1046	1047	1048	1049	1050
Alpha-Latrotoxin L7X8P2	S. grossa	/	E	I	N	R	D	C	V	R	G	-	-	E	T	A	I	D	M	A	I	S	K	N	H	I	N	V	V	R	Y	L	V	K	K	G	A	D	L	R	R	R	N	A	L	G	S	T	P	F	F	T
Alpha-Latrotoxin L7XCU0	L. geometricus	/	K	V	N	H	D	C	A	N	G	-	-	M	T	A	I	D	K	A	I	T	K	N	Q	L	Q	V	V	Q	I	L	A	E	N	G	V	D	F	R	R	K	N	R	L	D	A	T	P	F	L	T
Alpha-Latrotoxin-Sn1a	S. nobilis	This Study	D	V	N	K	R	C	M	F	Y	-	-	Q	S	A	L	D	M	A	I	M	G	N	K	L	E	I	V	K	Y	L	A	E	N	G	A	K	I	R	R	S	D	I	A	G	N	S	P	L	H	N
Alpha-Latrotoxin-Sn1b	S. nobilis	This Study	N	I	K	A	S	R	H	F	V	N	A	-	-	-	-	-	-	A	A	Q	I	P	R	P	E	I	L	Q	Y	I	V	D	Q	G	V	D	L	K	E	N	S	S	S	F	P	L	I	Q	T	I
Alpha-Latrotoxin-Sn1c	S. nobilis	This Study	N	I	H	T	N	C	N	F	-	-	-	Q	L	A	I	D	I	A	I	Q	F	D	H	L	N	I	V	K	F	L	V	E	N	G	A	D	L	K	K	R	N	R	Q	G	Y	S	P	L	G	S
Alpha-Latrotoxin-Sn1d	S. nobilis	This Study	N	I	H	T	N	C	N	F	-	-	-	Q	L	A	I	D	I	A	I	Q	F	D	H	L	N	I	V	K	F	L	V	E	N	G	A	D	L	K	K	R	N	R	Q	G	Y	S	P	L	G	S
Alpha-Latrotoxin-Sn1e	S. nobilis	This Study	N	I	H	T	N	C	N	F	-	-	-	Q	L	A	I	D	I	A	I	Q	F	D	H	L	N	I	V	K	F	L	V	E	N	G	A	D	L	K	K	R	N	R	Q	G	Y	S	P	L	G	S

IDENTIFICATION	SPECIES	REF	1051	1052	1053	1054	1055	1056	1057	1058	1059	1060	1061	1062	1063	1064	1065	1066	1067	1068	1069	1070	1071	1072	1073	1074	1075	1076	1077	1078	1079	1080	1081	1082	1083	1084	1085	1086	1087	1088	1089	1090	1091	1092	1093	1094	1095	1096	1097	1098	1099	1100
Alpha-Latrotoxin L7X8P2	S. grossa	/	A	V	S	S	N	S	L	D	I	A	R	Y	F	I	N	E	K	I	G	D	I	D	I	N	E	Q	N	E	K	G	E	S	A	L	H	L	A	V	Y	Y	R	N	L	-	-	-	-	Q	M	V
Alpha-Latrotoxin L7XCU0	L. geometricus	/	A	V	A	S	N	S	Y	E	I	A	E	Y	L	I	R	E	K	R	Q	N	I	N	I	N	E	Q	N	G	N	K	E	T	A	L	H	L	A	V	Y	Y	K	N	L	-	-	-	-	Q	M	I
Alpha-Latrotoxin-Sn1a	S. nobilis	This Study	A	A	I	N	E	R	L	E	A	A	K	Y	L	V	E	N	H	G	-	-	A	E	I	N	D	T	N	N	D	R	D	T	P	L	H	L	A	A	Q	H	G	C	L	-	-	-	-	D	M	V
Alpha-Latrotoxin-Sn1b	S. nobilis	This Study	A	M	N	V	Y	C	F	D	C	L	K	Y	V	V	E	N	K	L	-	-	L	N	V	N	D	K	N	Y	N	G	D	T	I	C	H	-	I	I	V	K	N	M	H	P	L	A	K	K	I	I
Alpha-Latrotoxin-Sn1c	S. nobilis	This Study	A	F	R	Y	Q	K	Y	E	I	L	K	Y	F	I	E	E	K	T	H	L	I	N	I	N	E	R	D	F	I	N	D	T	P	L	I	T	A	V	C	V	G	R	L	-	-	-	-	D	V	V
Alpha-Latrotoxin-Sn1d	S. nobilis	This Study	A	F	R	Y	Q	K	Y	E	I	L	K	Y	F	I	E	E	K	T	H	L	I	N	I	N	E	R	D	F	I	N	D	T	P	L	I	T	A	V	C	V	G	R	L	-	-	-	-	D	V	V
Alpha-Latrotoxin-Sn1e	S. nobilis	This Study	A	F	R	Y	Q	K	Y	E	I	L	K	Y	F	I	E	E	K	T	H	L	I	N	I	N	E	R	D	F	I	N	D	T	P	L	I	T	A	V	C	V	G	R	L	-	-	-	-	D	V	V

IDENTIFICATION	SPECIES	REF	1101	1102	1103	1104	1105	1106	1107	1108	1109	1110	1111	1112	1113	1114	1115	1116	1117	1118	1119	1120	1121	1122	1123	1124	1125	1126	1127	1128	1129	1130	1131	1132	1133	1134	1135	1136	1137	1138	1139	1140	1141	1142	1143	1144	1145	1146	1147	1148	1149	1150
Alpha-Latrotoxin L7X8P2	S. grossa	/	K	L	L	V	E	N	G	I	N	V	G	I	R	N	A	I	G	K	T	A	L	E	I	A	R	D	A	R	F	S	R	I	V	E	Y	L	N	T	K	S	R	R	F	R	K	G	-	-	-	-
Alpha-Latrotoxin L7XCU0	L. geometricus	/	K	L	L	K	Y	G	I	D	E	N	I	R	N	G	Y	D	K	T	A	L	D	I	A	R	D	N	K	I	S	S	I	V	E	Y	L	Q	T	K	S	G	K	F	R	R	E	-	-	-	-	-
Alpha-Latrotoxin-Sn1a	S. nobilis	This Study	K	Y	F	V	E	K	G	A	D	I	F	L	P	N	R	E	G	K	T	A	L	D	V	A	K	F	Y	S	N	K	D	I	I	S	Y	L	E	S	K	S	R	F	L	R	N	V	T	P	L	Q
Alpha-Latrotoxin-Sn1b	S. nobilis	This Study	S	Y	L	C	S	Q	E	A	D	V	T	I	R	N	N	D	D	K	T	A	Y	E	I	A	E	E	N	G	T	E	S	L	Y	P	C	L	K	P	N	T	Y	-	-	-	-	-	-	-	-	-
Alpha-Latrotoxin-Sn1c	S. nobilis	This Study	K	S	L	V	E	H	G	A	D	L	T	A	K	N	N	D	G	E	T	A	L	D	A	A	R	N	L	K	Q	Y	K	T	I	E	Y	L	Q	S	R	S	K	R	F	I	Q	R	-	-	-	-
Alpha-Latrotoxin-Sn1d	S. nobilis	This Study	K	S	L	V	E	H	G	A	D	L	T	A	K	N	N	D	G	E	T	A	L	D	A	A	R	N	L	K	Q	Y	K	T	I	E	Y	L	Q	S	R	S	K	R	F	I	Q	R	-	-	-	-
Alpha-Latrotoxin-Sn1e	S. nobilis	This Study	K	S	L	V	E	H	G	A	D	L	T	A	K	N	N	D	G	E	T	A	L	D	A	A	R	N	L	K	Q	Y	K	T	I	E	Y	L	Q	S	R	S	K	R	F	I	Q	R	-	-	-	-

IDENTIFICATION	SPECIES	REF	1151	1152	1153	1154	1155	1156	1157	1158	1159	1160	1161	1162	1163	1164	1165	1166	1167	1168	1169	1170	1171	1172	1173	1174	1175	1176	1177	1178	1179	1180	1181	1182	1183	1184	1185	1186	1187	1188	1189	1190	1191	1192	1193	1194	1195	1196	1197	1198	1199	1200
Alpha-Latrotoxin L7X8P2	S. grossa	/	-	-	F	Q	I	L	R	N	A	Q	G	Y	P	-	-	-	-	-	-	-	-	-	H	M	N	M	I	S	N	F	I	G	-	S	K	N	I	E	-	-	-	-	-	-	-	-	-	-	-	-
Alpha-Latrotoxin L7XCU0	L. geometricus	/	-	-	Y	K	S	S	N	G	E	H	D	L	L	-	-	-	-	-	-	-	-	-	H	E	N	R	I	S	N	F	T	D	-	S	K	N	L	E	-	-	-	-	-	-	-	-	-	-	-	-
Alpha-Latrotoxin-Sn1a	S. nobilis	This Study	G	S	M	D	F	S	T	N	K	R	F	I	P	-	-	-	-	-	-	-	-	-	Y	N	N	V	A	L	N	S	R	A	V	K	R	E	I	R	-	-	-	-	-	-	-	-	-	-	-	-
Alpha-Latrotoxin-Sn1b	S. nobilis	This Study	-	-	-	-	-	-	-	-	-	-	-	-	-	N	L	Y	K	R	S	T	H	S	Y	V	S	S	K	-	-	-	-	-	-	N	G	N	R	L	Y	R	K	A	A	S	Q	S	E	N	D	S
Alpha-Latrotoxin-Sn1c	S. nobilis	This Study	-	-	A	M	-	-	-	-	K	N	N	V	P	-	-	-	-	-	-	-	-	-	Y	I	N	F	S	S	N	M	K	L	-	N	K	E	T	G	-	-	-	-	-	-	-	-	-	-	-	-
Alpha-Latrotoxin-Sn1d	S. nobilis	This Study	-	-	A	M	-	-	-	-	K	N	N	V	P	-	-	-	-	-	-	-	-	-	Y	I	N	F	S	S	N	M	K	L	-	N	K	E	T	G	-	-	-	-	-	-	-	-	-	-	-	-
Alpha-Latrotoxin-Sn1e	S. nobilis	This Study	-	-	A	M	-	-	-	-	K	N	N	V	P	-	-	-	-	-	-	-	-	-	Y	I	N	F	S	S	N	M	K	L	-	N	K	E	T	G	-	-	-	-	-	-	-	-	-	-	-	-

IDENTIFICATION	SPECIES	REF	1201	1202	1203	1204	1205	1206	1207	1208	1209	1210	1211	1212	1213	1214	1215	1216	1217	1218	1219	1220	1221	1222	1223	1224	1225	1226	1227	1228	1229	1230	1231	1232	1233	1234	1235	1236	1237	1238	1239	1240	1241	1242	1243	1244	1245	1246	1247	1248	1249	1250
Alpha-Latrotoxin L7X8P2	S. grossa	/	-	-	H	D	H	Q	R	F	L	N	S	D	V	E	E	S	K	-	-	-	-	-	-	-	-	-	-	-	-	-	-	-	-	-	-	-	T	F	L	N	S	P	S	F	Q	N	I	D	V	I
Alpha-Latrotoxin L7XCU0	L. geometricus	/	-	-	Y	Q	H	Q	H	F	I	N	A	D	N	D	A	S	Q	-	-	-	-	-	-	-	-	-	-	-	-	-	-	-	-	-	-	-	S	F	S	N	T	A	A	N	Q	N	I	D	V	I
Alpha-Latrotoxin-Sn1a	S. nobilis	This Study	-	-	Y	N	N	K	K	F	A	N	E	N	D	D	A	G	N	-	-	-	-	-	-	-	-	-	-	-	-	-	-	-	-	-	-	-	K	I	S	G	S	N	S	P	Q	D	I	N	I	I
Alpha-Latrotoxin-Sn1b	S. nobilis	This Study	R	Y	Y	K	T	K	D	L	F	Y	G	R	V	G	N	I	R	S	I	P	K	S	P	R	S	T	L	A	I	T	G	D	T	T	Y	S	A	F	P	E	R	Y	L	N	P	N	I	N	V	N
Alpha-Latrotoxin-Sn1c	S. nobilis	This Study	-	-	F	S	N	P	K	Y	I	N	G	E	E	D	I	I	R	-	-	-	-	-	-	-	-	-	-	-	-	-	-	-	-	-	-	-	L	K	K	N	N	V	D	I	R	N	V	D	V	I
Alpha-Latrotoxin-Sn1d	S. nobilis	This Study	-	-	F	S	N	P	K	Y	I	N	G	E	E	D	I	I	R	-	-	-	-	-	-	-	-	-	-	-	-	-	-	-	-	-	-	-	L	K	K	N	N	V	D	I	R	N	V	D	V	I
Alpha-Latrotoxin-Sn1e	S. nobilis	This Study	-	-	F	S	N	P	K	Y	I	N	G	E	E	D	I	I	R	-	-	-	-	-	-	-	-	-	-	-	-	-	-	-	-	-	-	-	L	K	K	N	N	V	D	I	R	N	V	D	V	I

IDENTIFICATION	SPECIES	REF	1251	1252	1253	1254	1255	1256	1257	1258	1259	1260	1261	1262	1263	1264	1265	1266	1267	1268	1269	1270	1271	1272	1273	1274	1275	1276	1277	1278	1279	1280	1281	1282	1283	1284	1285	1286	1287	1288	1289	1290	1291	1292	1293	1294	1295	1296	1297	1298	1299	1300
Alpha-Latrotoxin L7X8P2	S. grossa	/	G	T	L	L	L	V	D	V	L	I	R	Y	F	T	K	Q	G	Y	I	N	E	E	N	D	S	A	S	D	E	G	V	Q	A	A	A	L	E	I	T	E	N	F	E	N	F	L	N	V	F	D
Alpha-Latrotoxin L7XCU0	L. geometricus	/	G	T	L	L	L	I	D	V	L	I	R	R	F	S	K	Q	G	Y	I	N	N	K	P	D	S	A	S	D	G	I	A	Q	A	A	A	L	A	I	T	E	K	F	E	D	I	L	N	S	L	H
Alpha-Latrotoxin-Sn1a	S. nobilis	This Study	S	T	L	L	L	I	D	V	I	I	R	M	F	T	G	E	K	Y	I	N	T	T	G	D	S	L	S	E	T	D	A	Q	A	Y	A	L	K	I	V	E	K	F	E	K	L	F	N	L	F	S
Alpha-Latrotoxin-Sn1b	S. nobilis	This Study	S	N	L	L	L	I	D	F	F	L	K	A	L	T	G	Q	K	Y	K	S	I	D	N	V	S	I	S	D	F	E	A	Q	E	A	A	I	D	I	I	D	R	F	G	K	V	L	K	S	F	S
Alpha-Latrotoxin-Sn1c	S. nobilis	This Study	G	T	L	M	L	L	D	V	I	I	R	Y	F	T	N	Q	K	Y	I	G	E	G	N	D	S	V	S	D	A	E	A	Q	A	T	A	L	K	I	T	E	K	F	E	N	L	L	N	F	F	Q
Alpha-Latrotoxin-Sn1d	S. nobilis	This Study	G	T	L	M	L	L	D	V	I	I	R	Y	F	T	N	Q	K	Y	I	G	E	G	N	D	S	V	S	D	A	E	A	Q	A	T	A	L	K	I	T	E	K	F	E	N	L	L	N	F	F	Q
Alpha-Latrotoxin-Sn1e	S. nobilis	This Study	G	T	L	M	L	L	D	V	I	I	R	Y	F	T	N	Q	K	Y	I	G	E	G	N	D	S	V	S	D	A	E	A	Q	A	T	A	L	K	I	T	E	K	F	E	N	L	L	N	F	F	Q

IDENTIFICATION	SPECIES	REF	1301	1302	1303	1304	1305	1306	1307	1308	1309	1310	1311	1312	1313	1314	1315	1316	1317	1318	1319	1320	1321	1322	1323	1324	1325	1326	1327	1328	1329	1330	1331	1332	1333	1334	1335	1336	1337	1338	1339	1340	1341	1342	1343	1344	1345	1346	1347	1348	1349	1350
Alpha-Latrotoxin L7X8P2	S. grossa	/	-	D	T	P	K	E	Q	V	D	L	T	E	V	H	R	K	V	Y	A	A	L	K	S	G	R	K	S	Q	I	H	Q	V	L	C	S	S	L	K	S	V	S	K	L	E	P	A	E	I	E	T
Alpha-Latrotoxin L7XCU0	L. geometricus	/	N	K	S	T	E	E	Q	V	D	F	A	E	V	H	R	K	I	Y	A	A	L	R	S	G	R	N	S	Q	V	H	Q	V	L	C	S	S	L	K	S	I	S	T	L	K	P	E	D	I	K	K
Alpha-Latrotoxin-Sn1a	S. nobilis	This Study	N	L	P	T	G	E	E	I	D	L	S	E	V	H	A	N	I	Y	K	A	L	K	S	G	K	G	S	K	I	H	E	I	L	C	S	Y	F	D	M	L	T	S	L	N	P	E	E	K	E	K
Alpha-Latrotoxin-Sn1b	S. nobilis	This Study	N	R	P	V	E	V	H	V	D	M	S	K	I	H	S	D	V	Y	K	A	I	K	R	G	T	A	N	E	I	P	N	I	L	Y	S	H	V	K	S	F	A	D	L	N	S	K	Q	I	A	E
Alpha-Latrotoxin-Sn1c	S. nobilis	This Study	-	N	L	P	E	E	Q	I	D	F	A	E	V	H	N	N	I	Y	K	A	I	K	N	D	N	Q	N	E	I	Q	Q	V	L	C	S	S	V	K	T	L	S	T	V	D	V	E	E	M	D	E
Alpha-Latrotoxin-Sn1d	S. nobilis	This Study	-	N	L	P	E	E	Q	I	D	F	A	E	V	H	N	N	I	Y	K	A	I	K	N	D	N	Q	N	E	I	Q	Q	V	L	C	S	S	V	K	T	L	S	T	V	D	V	E	E	M	D	E
Alpha-Latrotoxin-Sn1e	S. nobilis	This Study	-	N	L	P	E	E	Q	I	D	F	A	E	V	H	N	N	I	Y	K	A	I	K	N	D	N	Q	N	E	I	Q	Q	V	L	C	S	S	V	K	T	L	S	T	V	D	V	E	E	M	D	E

IDENTIFICATION	SPECIES	REF	1351	1352	1353	1354	1355	1356	1357	1358	1359	1360	1361	1362	1363	1364	1365	1366	1367	1368	1369	1370	1371	1372	1373	1374	1375	1376	1377	1378	1379	1380	1381	1382	1383	1384	1385	1386	1387	1388	1389	1390	1391	1392	1393	1394	1395	1396	1397	1398
Alpha-Latrotoxin L7X8P2	S. grossa	/	L	V	S	E	L	L	N	L	Q	S	S	F	P	L	T	K	F	P	P	S	T	A	E	A	F	E	N	S	L	H	I	L	G	E	S	C	L	N	S	E	W	S	P	S	L	M	L	M
Alpha-Latrotoxin L7XCU0	L. geometricus	/	L	V	S	V	V	L	S	F	Q	S	S	I	S	L	P	E	V	S	A	S	A	R	E	V	Y	G	E	T	L	H	L	F	H	E	S	-	-	-	-	-	-	-	-	-	-	-	-	-
Alpha-Latrotoxin-Sn1a	S. nobilis	This Study	L	V	A	A	F	N	S	T	L	P	S	I	K	N	A	E	S	P	S	S	S	A	P	D	F	K	N	L	P	S	A	I	I	K	S	C	F	N	L	E	E	N	S	S	L	L	R	I
Alpha-Latrotoxin-Sn1b	S. nobilis	This Study	L	L	S	-	-	-	-	-	-	-	-	-	-	-	-	-	-	-	-	-	-	-	-	-	-	-	-	-	-	-	-	-	-	-	-	-	-	-	-	-	-	-	-	-	-	-	-	-
Alpha-Latrotoxin-Sn1c	S. nobilis	This Study	L	L	S	E	L	L	S	T	H	P	P	G	N	S	T	L	-	-	-	-	S	I	A	E	S	A	A	T	Y	Q	L	L	A	Q	H	C	L	E	-	-	-	-	-	-	-	-	-	-
Alpha-Latrotoxin-Sn1d	S. nobilis	This Study	L	L	S	E	L	L	S	T	H	P	P	G	N	S	T	L	-	-	-	-	S	I	A	E	S	A	A	T	Y	Q	L	L	A	Q	H	C	L	E	-	-	-	-	-	-	-	-	-	-
Alpha-Latrotoxin-Sn1e	S. nobilis	This Study	L	L	S	E	L	L	S	T	H	P	P	G	N	S	T	L	-	-	-	-	S	I	A	E	S	A	A	T	Y	Q	L	L	A	Q	H	C	L	E	-	-	-	-	-	-	-	-	-	-

Supplementary dataset 2 – Table 4a- Delta-Latroinsectotoxins- Sequences from position 1 to 400: Sequence alignments of the closest two delta-latroinsectotoxins already published and the five most abundant found in the venom of *Steatoda nobilis*. Yellow windows indicate positions where the identity and/or the physico-chemical properties of the aminoacids is maintained at least for 4 possibilities over 7, bold positions are the ones where the positions are maintained 2 or 3 times (over 7). Cysteines are highlighted in red.

IDENTIFICATION	SPECIES	REF	1	2	3	4	5	6	7	8	9	10	11	12	13	14	15	16	17	18	19	20	21	22	23	24	25	26	27	28	29	30	31	32	33	34	35	36	37	38	39	40	41	42	43	44	45	46	47	48	49	50
Delta-latroinsectotoxin-Lt1a Q25338	*L. tredecimguttatus*	[1]	D	E	E	D	G	E	M	T	L	E	E	R	Q	A	Q	C	K	A	I	E	Y	S	N	S	V	F	G	M	I	A	D	V	A	N	D	I	G	S	I	P	V	I	G	E	V	V	G	I	V	T
Delta-latroinsectotoxin-XP_015914373.1	*P. tepidariorum*	/	D	-	-	-	-	L	N	E	I	A	E	R	K	Q	Q	C	Q	I	L	E	Y	A	S	I	A	T	E	L	V	G	N	L	A	S	D	V	S	D	I	P	V	V	G	E	V	A	G	V	Y	T
Delta-latroinsectotoxin-Sno1a	*S. nobilis*	*This study*	F	L	R	G	G	N	M	T	L	E	E	R	A	E	K	C	Q	T	I	E	Y	R	N	K	I	T	G	F	M	D	N	M	A	S	D	V	G	D	I	P	V	F	G	E	V	A	K	A	F	T
Delta-latroinsectotoxin-Sno1b	*S. nobilis*	*This study*	E	-	E	E	E	E	M	S	L	E	E	R	Q	A	Q	C	K	A	I	E	Y	S	G	H	V	A	G	M	V	A	D	V	A	G	D	I	G	S	I	P	V	I	G	D	V	V	G	V	F	T
Delta-latroinsectotoxin-Sno1c	*S. nobilis*	*This study*	E	-	E	E	E	E	M	S	L	E	E	R	Q	A	Q	C	K	A	I	E	Y	S	G	H	V	A	G	M	V	A	D	V	A	G	D	I	G	S	I	P	V	I	G	D	V	V	G	V	F	T
Delta-latroinsectotoxin-Sno1d	*S. nobilis*	*This study*	-	-	-	-	-	-	-	-	-	-	-	-	-	-	-	-	-	-	M	K	I	A	N	T	A	V	G	T	Y	G	D	N	F	S	L	L	T	S	I	P	V	I	G	-	-	-	-	M	F	F
Delta-latroinsectotoxin-Sno1e	*S. nobilis*	*This study*	-	-	R	K	T	K	T	A	W	E	K	K	Q	A	Q	C	K	A	L	E	Q	G	S	N	V	S	D	A	V	G	N	V	A	K	G	I	G	S	I	P	V	I	G	E	A	V	S	F	I	T

IDENTIFICATION	SPECIES	REF	51	52	53	54	55	56	57	58	59	60	61	62	63	64	65	66	67	68	69	70	71	72	73	74	75	76	77	78	79	80	81	82	83	84	85	86	87	88	89	90	91	92	93	94	95	96	97	98	99	100
Delta-latroinsectotoxin-Lt1a Q25338	*L. tredecimguttatus*	[1]	A	P	I	A	I	V	S	H	I	T	S	A	G	L	D	I	A	S	T	A	L	D	C	D	D	I	P	F	D	E	I	K	E	I	L	E	E	R	F	N	E	I	D	R	K	L	D	K	S	M
Delta-latroinsectotoxin-XP_015914373.1	*P. tepidariorum*	/	A	G	I	S	M	V	S	H	V	A	K	A	G	M	D	I	A	S	Q	A	L	D	C	D	D	V	N	F	D	E	I	K	E	I	M	D	E	R	F	N	E	V	N	R	K	L	D	K	Q	M
Delta-latroinsectotoxin-Sno1a	*S. nobilis*	*This study*	V	P	A	K	M	A	F	H	V	D	Q	F	K	Q	D	M	K	S	M	A	L	R	C	D	D	V	P	F	D	E	L	K	E	I	L	E	E	K	F	N	E	V	D	R	K	L	D	K	N	T
Delta-latroinsectotoxin-Sno1b	*S. nobilis*	*This study*	A	P	I	A	I	V	A	H	I	T	S	A	G	L	D	I	A	S	T	A	L	D	C	D	D	I	P	F	D	E	I	K	Q	I	L	E	E	R	F	N	E	V	D	R	K	L	D	K	N	T
Delta-latroinsectotoxin-Sno1c	*S. nobilis*	*This study*	A	P	I	A	I	V	A	H	I	T	S	A	G	L	D	I	A	S	T	A	L	D	C	D	D	I	P	F	D	E	I	K	Q	I	L	E	E	R	F	N	E	V	N	R	K	L	D	E	Q	T
Delta-latroinsectotoxin-Sno1d	*S. nobilis*	*This study*	V	P	V	S	M	A	F	N	V	V	K	G	A	L	D	I	T	Q	T	A	M	D	C	D	D	V	P	F	E	E	I	K	L	M	L	E	E	K	F	H	E	V	F	S	L	L	T	S	I	P
Delta-latroinsectotoxin-Sno1e	*S. nobilis*	*This study*	A	P	L	S	V	L	A	Y	V	T	S	A	G	L	N	I	A	S	S	A	L	D	C	D	K	L	P	F	D	E	I	K	N	L	L	N	E	R	F	N	E	V	D	R	K	L	D	Q	Q	A

IDENTIFICATION	SPECIES	REF	101	102	103	104	105	106	107	108	109	110	111	112	113	114	115	116	117	118	119	120	121	122	123	124	125	126	127	128	129	130	131	132	133	134	135	136	137	138	139	140	141	142	143	144	145	146	147	148	149	150
Delta-latroinsectotoxin-Lt1a Q25338	*L. tredecimguttatus*	[1]	E	A	L	E	E	V	S	R	L	V	S	K	T	M	L	I	V	D	Q	T	R	V	M	M	R	E	G	F	D	K	I	L	D	A	I	E	N	K	E	L	K	D	S	I	N	K	I	K	N	F
Delta-latroinsectotoxin-XP_015914373.1	*P. tepidariorum*	/	E	M	L	Q	E	I	S	K	S	V	K	E	T	Y	E	N	V	E	R	L	R	S	E	M	K	A	G	F	Q	D	I	L	E	T	I	D	I	K	E	I	R	A	I	V	F	R	I	N	N	F
Delta-latroinsectotoxin-Sno1a	*S. nobilis*	*This study*	A	A	L	E	E	V	S	K	L	V	S	K	T	Y	T	V	V	E	K	T	R	T	E	M	N	E	N	F	K	L	V	L	E	T	I	E	S	K	E	I	K	S	I	V	F	K	I	D	D	F
Delta-latroinsectotoxin-Sno1b	*S. nobilis*	*This study*	A	A	L	E	E	V	S	K	L	V	S	K	T	Y	T	V	V	E	K	T	R	T	E	M	N	E	N	F	K	L	V	L	E	T	I	E	S	K	E	I	K	S	I	V	F	K	I	D	D	F
Delta-latroinsectotoxin-Sno1c	*S. nobilis*	*This study*	A	L	L	K	R	V	H	N	L	V	G	R	N	F	E	A	I	E	R	S	R	L	E	M	K	E	G	F	Q	N	I	L	E	T	I	H	N	N	D	L	S	K	I	V	F	N	I	N	N	F
Delta-latroinsectotoxin-Sno1d	*S. nobilis*	*This study*	V	I	G	-	-	-	-	M	F	F	V	P	V	S	M	A	F	N	V	V	K	G	A	L	D	I	T	Q	T	A	M	D	C	D	D	V	P	F	E	E	I	K	L	M	L	E	E	K	F	H
Delta-latroinsectotoxin-Sno1e	*S. nobilis*	*This study*	A	A	L	D	K	V	S	K	I	A	S	K	T	F	A	E	V	E	N	T	R	S	E	M	N	E	G	F	K	L	V	L	D	T	I	Q	S	Q	S	I	K	S	I	V	F	S	I	N	N	F

IDENTIFICATION	SPECIES	REF	151	152	153	154	155	156	157	158	159	160	161	162	163	164	165	166	167	168	169	170	171	172	173	174	175	176	177	178	179	180	181	182	183	184	185	186	187	188	189	190	191	192	193	194	195	196	197	198	199	200
Delta-latroinsectotoxin-Lt1a Q25338	*L. tredecimguttatus*	[1]	V	K	F	F	E	K	E	R	Q	R	I	K	G	L	P	K	D	R	Y	V	A	K	L	L	E	Q	K	G	I	L	G	S	L	K	E	V	R	E	P	S	G	N	S	L	S	S	A	L	N	E
Delta-latroinsectotoxin-XP_015914373.1	*P. tepidariorum*	/	I	E	Y	F	E	N	E	K	R	A	M	R	S	L	P	E	D	Q	Y	I	F	K	L	Q	E	P	N	S	V	L	T	Y	L	R	N	T	R	L	P	N	E	D	S	L	H	S	A	L	H	K
Delta-latroinsectotoxin-Sno1a	*S. nobilis*	*This study*	K	G	Y	F	E	M	E	R	N	E	M	K	G	M	N	K	N	E	Y	I	G	K	L	E	E	E	N	G	L	L	V	Y	L	K	N	S	R	T	P	R	G	D	S	L	S	S	A	L	K	E
Delta-latroinsectotoxin-Sno1b	*S. nobilis*	*This study*	K	G	F	F	D	K	E	R	Q	R	I	K	G	L	P	K	D	K	Y	V	A	K	L	L	E	Q	K	G	I	L	S	S	L	K	E	V	R	E	P	S	G	N	S	L	G	S	A	L	N	E
Delta-latroinsectotoxin-Sno1c	*S. nobilis*	*This study*	V	T	F	F	D	K	E	R	Q	R	I	K	G	L	P	K	D	K	Y	V	A	K	L	L	E	Q	K	G	I	L	S	S	L	K	E	V	R	E	P	S	G	N	S	L	G	S	A	L	N	E
Delta-latroinsectotoxin-Sno1d	*S. nobilis*	*This study*	E	V	Y	F	D	N	E	R	A	S	M	R	G	D	S	K	E	R	Y	I	A	K	L	Q	D	P	D	S	I	L	V	Y	L	K	S	M	R	T	S	S	E	D	S	L	D	Y	A	L	F	E
Delta-latroinsectotoxin-Sno1e	*S. nobilis*	*This study*	I	R	Y	F	D	E	E	R	E	K	L	K	G	L	S	K	D	N	Y	I	A	K	L	Q	E	Q	D	S	I	L	F	Y	L	K	D	S	R	T	P	K	G	D	S	L	S	S	A	L	Y	K

IDENTIFICATION	SPECIES	REF	201	202	203	204	205	206	207	208	209	210	211	212	213	214	215	216	217	218	219	220	221	222	223	224	225	226	227	228	229	230	231	232	233	234	235	236	237	238	239	240	241	242	243	244	245	246	247	248	249	250
Delta-latroinsectotoxin-Lt1a Q25338	*L. tredecimguttatus*	[1]	L	L	D	K	N	N	N	Y	A	I	P	K	V	V	D	D	N	K	A	F	Q	A	L	Y	A	L	F	Y	G	T	Q	T	Y	A	A	V	M	F	F	L	L	E	Q	H	S	Y	L	A	D	Y
Delta-latroinsectotoxin-XP_015914373.1	*P. tepidariorum*	/	I	I	D	S	-	-	D	Y	A	I	P	K	N	A	N	D	S	K	A	F	S	A	L	H	A	L	F	H	G	A	Q	T	Y	V	S	I	M	F	F	L	L	K	E	H	S	Y	L	A	D	Y
Delta-latroinsectotoxin-Sno1a	*S. nobilis*	*This study*	L	I	D	P	E	N	H	Y	A	V	P	K	A	E	N	D	R	K	A	F	Q	A	L	F	A	L	F	Y	G	T	Q	A	Y	V	S	V	M	F	F	L	L	E	Q	H	S	F	L	A	D	Y
Delta-latroinsectotoxin-Sno1b	*S. nobilis*	*This study*	L	L	D	K	N	N	N	Y	A	I	P	K	V	A	D	D	N	K	A	F	Q	A	L	Y	A	L	F	Y	G	T	Q	T	Y	A	S	V	M	Y	F	L	L	E	Q	H	S	Y	L	A	D	Y
Delta-latroinsectotoxin-Sno1c	*S. nobilis*	*This study*	L	L	D	K	N	N	N	Y	A	I	P	K	V	A	D	D	N	K	A	F	Q	A	L	Y	A	L	F	Y	G	T	Q	T	Y	A	S	V	M	Y	F	L	L	E	Q	H	S	Y	L	A	D	Y
Delta-latroinsectotoxin-Sno1d	*S. nobilis*	*This study*	L	M	E	E	D	K	M	Y	V	I	P	K	S	I	D	D	E	N	A	F	K	A	L	N	A	L	F	Y	G	T	Q	S	Y	V	S	I	M	F	F	L	V	E	Q	Y	S	Y	L	A	E	Y
Delta-latroinsectotoxin-Sno1e	*S. nobilis*	*This study*	L	I	D	K	N	N	N	Y	A	I	P	K	V	F	D	D	N	K	A	Y	Q	A	L	Y	G	L	F	Y	G	T	Q	A	Y	V	S	I	M	F	F	L	L	G	E	F	S	Y	L	A	D	Y

IDENTIFICATION	SPECIES	REF	251	252	253	254	255	256	257	258	259	260	261	262	263	264	265	266	267	268	269	270	271	272	273	274	275	276	277	278	279	280	281	282	283	284	285	286	287	288	289	290	291	292	293	294	295	296	297	298	299	300
Delta-latroinsectotoxin-Lt1a Q25338	*L. tredecimguttatus*	[1]	Y	Y	Q	K	G	D	D	V	N	F	N	A	E	F	N	N	V	A	I	I	F	D	D	F	K	S	S	L	T	G	G	-	-	-	-	D	D	G	L	I	D	N	V	I	E	V	L	N	-	T
Delta-latroinsectotoxin-XP_015914373.1	*P. tepidariorum*	/	N	Y	Q	K	G	D	M	E	E	F	N	K	Q	F	D	T	L	K	S	I	F	E	D	F	Q	T	S	L	T	G	G	T	S	-	-	N	N	S	F	L	D	K	A	I	E	T	L	N	-	K
Delta-latroinsectotoxin-Sno1a	*S. nobilis*	*This study*	Y	Y	E	Q	G	E	T	A	K	F	N	N	Q	F	E	K	I	L	T	L	F	K	G	F	K	T	S	L	I	G	S	I	K	N	-	P	K	P	L	L	D	K	V	I	K	I	F	K	T	D
Delta-latroinsectotoxin-Sno1b	*S. nobilis*	*This study*	Y	Y	Q	K	G	D	D	E	A	F	N	R	E	F	S	L	V	S	T	I	F	D	D	F	K	S	S	L	T	G	G	-	-	-	-	D	D	G	L	V	D	N	V	I	A	V	L	K	-	T
Delta-latroinsectotoxin-Sno1c	*S. nobilis*	*This study*	Y	Y	Q	K	G	D	D	E	A	F	N	R	E	F	S	L	V	S	T	I	F	D	D	F	K	S	S	L	T	G	G	-	-	-	-	D	D	G	L	V	D	N	V	V	A	V	L	K	-	I
Delta-latroinsectotoxin-Sno1d	*S. nobilis*	*This study*	H	Y	R	E	G	N	Y	E	E	F	N	K	N	F	N	N	V	E	V	T	F	E	E	F	K	S	S	L	I	G	K	D	Q	G	V	R	G	N	V	I	D	N	V	I	E	R	L	N	-	R
Delta-latroinsectotoxin-Sno1e	*S. nobilis*	*This study*	H	Y	E	S	G	N	D	E	K	F	N	R	Y	F	D	L	V	I	N	T	Y	K	D	F	K	F	S	L	V	G	G	S	H	E	G	H	D	V	L	I	D	D	V	I	D	T	L	N	-	T

IDENTIFICATION	SPECIES	REF	301	302	303	304	305	306	307	308	309	310	311	312	313	314	315	316	317	318	319	320	321	322	323	324	325	326	327	328	329	330	331	332	333	334	335	336	337	338	339	340	341	342	343	344	345	346	347	348	349	350
Delta-latroinsectotoxin-Lt1a Q25338	*L. tredecimguttatus*	[1]	V	K	A	L	P	F	I	K	N	A	D	S	K	L	Y	R	E	L	V	T	R	T	K	A	L	E	T	L	K	N	Q	I	K	T	T	D	L	P	L	I	D	D	I	P	E	T	L	S	Q	V
Delta-latroinsectotoxin-XP_015914373.1	*P. tepidariorum*	/	A	K	D	K	A	F	V	K	D	L	E	S	K	L	Y	Q	E	L	S	H	R	E	G	N	L	R	K	L	K	R	E	I	K	S	I	K	L	P	I	I	S	D	V	P	K	T	A	A	Q	I
Delta-latroinsectotoxin-Sno1a	*S. nobilis*	*This study*	V	L	D	K	N	I	I	K	N	R	N	S	K	L	Y	Q	T	V	V	N	Y	T	S	T	L	R	K	L	K	L	D	I	V	L	T	K	L	P	L	I	K	D	I	P	K	P	P	V	Q	I
Delta-latroinsectotoxin-Sno1b	*S. nobilis*	*This study*	V	K	D	K	P	F	M	K	N	A	D	S	K	L	Y	R	D	I	V	N	R	T	N	T	L	S	D	L	K	T	Q	I	K	M	T	E	L	P	L	I	D	D	I	P	Q	N	P	V	Q	I
Delta-latroinsectotoxin-Sno1c	*S. nobilis*	*This study*	V	K	D	K	P	F	M	K	N	A	D	S	K	L	Y	R	D	I	V	N	R	T	N	T	L	S	D	L	K	T	Q	I	K	M	T	E	L	P	L	I	D	D	I	P	Q	N	P	V	Q	I
Delta-latroinsectotoxin-Sno1d	*S. nobilis*	*This study*	V	K	N	K	N	F	I	R	Y	P	K	T	K	F	H	Y	D	I	V	N	Q	T	N	T	L	K	Q	L	K	S	Q	I	K	Y	M	I	L	P	L	I	K	S	T	P	E	N	T	I	T	I
Delta-latroinsectotoxin-Sno1e	*S. nobilis*	*This study*	V	K	E	K	K	F	I	R	H	V	E	S	K	L	Y	Q	D	I	A	N	R	T	E	V	L	H	S	L	K	D	Q	I	K	E	M	E	L	P	L	I	E	D	R	P	K	N	A	I	Q	I

IDENTIFICATION	SPECIES	REF	351	352	353	354	355	356	357	358	359	360	361	362	363	364	365	366	367	368	369	370	371	372	373	374	375	376	377	378	379	380	381	382	383	384	385	386	387	388	389	390	391	392	393	394	395	396	397	398	399	400
Delta-latroinsectotoxin-Lt1a Q25338	*L. tredecimguttatus*	[1]	N	F	P	N	D	E	N	Q	L	P	T	P	I	G	N	W	V	D	G	V	E	V	R	Y	A	V	Q	Y	E	S	K	G	M	Y	S	K	F	S	E	W	S	E	P	F	T	V	Q	G	N	A
Delta-latroinsectotoxin-XP_015914373.1	*P. tepidariorum*	/	S	F	E	D	-	-	S	A	L	S	A	P	H	G	D	W	E	D	R	A	K	V	R	Y	A	V	Q	L	E	K	N	G	L	F	S	K	L	S	E	W	T	E	P	V	F	V	Q	G	K	A
Delta-latroinsectotoxin-Sno1a	*S. nobilis*	*This study*	N	F	N	N	-	-	S	A	L	S	P	P	L	A	Y	W	R	K	D	A	E	V	A	Y	A	V	Q	Y	K	K	N	G	T	Y	S	K	L	S	D	W	T	D	D	F	R	V	L	N	K	T
Delta-latroinsectotoxin-Sno1b	*S. nobilis*	*This study*	S	F	P	G	E	-	D	E	L	T	T	P	I	G	D	W	V	D	G	A	K	V	R	Y	A	V	Q	Y	E	D	K	G	K	Y	S	K	F	S	E	W	T	D	P	F	T	V	E	G	K	A
Delta-latroinsectotoxin-Sno1c	*S. nobilis*	*This study*	S	F	P	G	E	-	D	E	L	T	T	P	I	G	D	W	V	D	G	A	K	V	R	Y	A	V	Q	Y	E	D	K	G	K	Y	S	K	F	S	E	W	T	D	P	F	T	V	E	G	K	A
Delta-latroinsectotoxin-Sno1d	*S. nobilis*	*This study*	S	I	S	S	-	-	S	K	I	S	T	P	V	G	G	W	V	K	G	V	T	F	Q	Y	A	L	Q	Y	E	L	N	G	K	F	S	K	L	S	K	W	T	N	K	N	T	I	H	E	T	A
Delta-latroinsectotoxin-Sno1e	*S. nobilis*	*This study*	S	F	N	E	-	-	P	L	S	P	T	P	L	G	E	W	V	D	G	T	E	V	T	Y	A	V	Q	Y	E	Y	M	G	K	F	S	K	I	S	E	W	S	D	P	F	T	V	R	G	K	A

Supplementary dataset 2 – Table 4b- Delta-Latroinsectotoxins- Sequences from position 401 to 800: Sequence alignments of the closest two delta-latroinsectotoxins already published and the five most abundant found in the venom of *Steatoda nobilis*. Yellow windows indicate positions where the identity and/or the physico-chemical properties of the aminoacids is maintained at least for 4 possibilities over 7, bold positions are the ones where the positions are maintained 2 or 3 times (over 7). Cysteines are highlighted in red.

IDENTIFICATION	SPECIES	REF	401	402	403	404	405	406	407	408	409	410	411	412	413	414	415	416	417	418	419	420	421	422	423	424	425	426	427	428	429	430	431	432	433	434	435	436	437	438	439	440	441	442	443	444	445	446	447	448	449	450
Delta-latroinsectotoxin-Lt1a Q25338	*L. tredecimguttatus*	[1]	C	P	T	I	K	V	R	V	D	P	K	-	K	R	N	R	L	I	F	R	K	F	N	S	G	K	P	Q	F	A	G	T	M	T	H	S	Q	T	N	F	K	D	I	H	R	D	L	Y	D	A
Delta-latroinsectotoxin-XP_015914373.1	*P. tepidariorum*	/	N	P	T	L	K	F	P	I	D	L	K	-	R	R	K	R	L	V	F	R	K	F	N	D	N	K	P	Q	L	V	G	V	L	S	K	L	Q	I	L	F	K	D	I	N	R	D	L	Y	N	A
Delta-latroinsectotoxin-Sno1a	*S. nobilis*	This study	N	P	I	L	K	V	A	I	D	Q	E	N	N	R	Y	R	L	I	F	R	K	F	K	D	H	E	A	Q	Y	I	G	M	L	S	E	T	Q	T	D	F	W	D	I	N	R	D	L	Y	D	A
Delta-latroinsectotoxin-Sno1b	*S. nobilis*	This study	S	P	V	L	K	V	P	V	D	K	K	-	R	R	N	R	L	I	F	R	K	V	N	S	E	K	Y	Q	F	A	G	V	M	S	H	T	Q	T	N	F	R	D	I	D	R	D	L	Y	N	A
Delta-latroinsectotoxin-Sno1c	*S. nobilis*	This study	S	P	V	L	K	V	P	V	D	K	K	-	R	R	N	R	L	I	F	R	K	V	N	S	E	K	Y	Q	F	A	G	V	M	S	H	T	Q	T	N	F	R	D	I	D	R	D	L	Y	N	A
Delta-latroinsectotoxin-Sno1d	*S. nobilis*	This study	N	P	K	L	T	I	P	V	D	P	N	-	N	N	N	R	L	I	F	R	R	F	N	N	H	K	P	Q	L	V	G	V	L	R	A	Y	Q	H	T	F	K	D	I	N	R	D	L	Y	N	A
Delta-latroinsectotoxin-Sno1e	*S. nobilis*	This study	N	P	T	I	K	V	P	V	D	Q	K	-	R	R	N	R	L	I	F	R	K	F	N	K	G	K	P	Q	L	V	H	I	M	K	K	T	E	T	S	F	R	D	L	N	R	D	L	Y	N	A

IDENTIFICATION	SPECIES	REF	451	452	453	454	455	456	457	458	459	460	461	462	463	464	465	466	467	468	469	470	471	472	473	474	475	476	477	478	479	480	481	482	483	484	485	486	487	488	489	490	491	492	493	494	495	496	497	498	499	500
Delta-latroinsectotoxin-Lt1a Q25338	*L. tredecimguttatus*	[1]	A	L	N	I	N	K	L	K	A	V	D	E	A	T	T	L	-	I	E	K	G	A	D	I	E	A	K	F	D	N	D	R	S	A	M	H	A	V	A	Y	R	G	N	N	K	I	A	L	R	F
Delta-latroinsectotoxin-XP_015914373.1	*P. tepidariorum*	/	A	G	R	V	D	K	K	L	A	L	K	E	I	N	L	L	-	I	E	N	G	A	D	I	N	A	V	F	D	S	D	R	S	V	M	H	A	A	A	K	S	G	N	D	K	A	A	L	R	I
Delta-latroinsectotoxin-Sno1a	*S. nobilis*	This study	A	M	G	V	D	K	D	L	S	I	S	D	V	Q	R	L	-	I	E	L	G	A	N	I	T	S	A	F	E	L	G	R	T	A	M	H	G	A	A	L	R	G	N	K	E	V	A	L	H	F
Delta-latroinsectotoxin-Sno1b	*S. nobilis*	This study	A	L	N	I	N	K	N	K	A	V	N	E	V	A	T	L	-	L	G	K	G	A	N	I	N	S	K	F	D	N	A	R	S	A	M	H	A	V	A	L	R	G	N	G	K	V	A	L	R	F
Delta-latroinsectotoxin-Sno1c	*S. nobilis*	This study	A	L	N	I	N	K	N	K	A	V	N	E	V	A	T	L	-	L	G	K	G	A	N	I	N	S	K	F	D	N	A	R	S	A	M	H	A	V	A	L	R	G	N	G	K	V	A	L	R	F
Delta-latroinsectotoxin-Sno1d	*S. nobilis*	This study	A	M	D	V	N	K	N	L	G	Y	K	D	F	M	K	L	L	Q	S	N	E	I	N	M	N	A	K	F	D	L	G	W	T	V	L	H	A	A	A	Y	I	G	N	F	E	I	V	K	H	V
Delta-latroinsectotoxin-Sno1e	*S. nobilis*	This study	A	L	S	V	D	K	D	I	A	M	D	E	V	K	N	L	-	I	D	I	G	A	D	I	S	S	V	F	D	Q	G	R	S	A	L	H	A	V	A	L	R	G	N	E	K	V	A	M	R	F

IDENTIFICATION	SPECIES	REF	501	502	503	504	505	506	507	508	509	510	511	512	513	514	515	516	517	518	519	520	521	522	523	524	525	526	527	528	529	530	531	532	533	534	535	536	537	538	539	540	541	542	543	544	545	546	547	548	549	550
Delta-latroinsectotoxin-Lt1a Q25338	*L. tredecimguttatus*	[1]	L	L	K	N	Q	S	I	D	I	E	L	K	D	K	N	G	F	T	P	L	H	I	A	A	E	A	G	Q	A	G	F	V	K	L	L	I	N	H	G	A	D	V	N	A	K	T	S	K	T	N
Delta-latroinsectotoxin-XP_015914373.1	*P. tepidariorum*	/	L	S	N	N	S	S	I	N	L	N	V	K	D	K	K	G	F	T	P	M	H	L	A	A	D	A	G	Q	A	E	F	I	K	M	L	I	Q	H	G	G	D	I	N	A	K	T	K	T	D	H
Delta-latroinsectotoxin-Sno1a	*S. nobilis*	This study	L	L	K	N	D	-	A	N	I	E	V	K	D	K	K	G	Y	T	P	L	H	V	A	A	K	A	G	Q	S	D	F	V	Q	L	L	I	T	Q	G	A	N	V	N	A	K	T	Y	V	H	R
Delta-latroinsectotoxin-Sno1b	*S. nobilis*	This study	L	L	N	D	E	D	T	D	I	E	L	R	D	K	N	G	Y	T	P	L	H	I	A	A	E	A	G	Q	S	G	F	V	K	L	L	I	N	H	G	A	D	V	N	A	K	A	V	K	K	N
Delta-latroinsectotoxin-Sno1c	*S. nobilis*	This study	L	L	N	D	E	D	T	D	I	E	L	R	D	K	N	G	Y	T	P	L	H	I	A	A	E	A	G	Q	S	G	F	V	K	L	L	I	N	H	G	A	D	V	N	A	K	A	V	K	K	N
Delta-latroinsectotoxin-Sno1d	*S. nobilis*	This study	L	L	N	D	D	T	F	D	I	N	E	A	D	T	E	G	F	T	P	L	H	I	A	C	K	H	S	R	E	D	V	V	E	F	L	V	E	H	G	A	D	V	T	A	K	T	K	T	G	L
Delta-latroinsectotoxin-Sno1e	*S. nobilis*	This study	L	L	K	N	N	S	T	D	I	N	I	R	D	K	R	G	Y	T	P	L	H	V	A	A	E	A	G	Q	S	G	F	V	N	L	L	L	S	H	E	A	D	V	N	A	K	T	E	K	Y	S

IDENTIFICATION	SPECIES	REF	551	552	553	554	555	556	557	558	559	560	561	562	563	564	565	566	567	568	569	570	571	572	573	574	575	576	577	578	579	580	581	582	583	584	585	586	587	588	589	590	591	592	593	594	595	596	597	598	599	600
Delta-latroinsectotoxin-Lt1a Q25338	*L. tredecimguttatus*	[1]	L	T	P	L	H	L	A	T	R	S	G	F	S	K	T	V	R	N	L	L	E	S	P	N	I	K	V	N	E	K	E	D	D	G	F	T	P	L	H	T	A	V	M	S	T	Y	M	V	V	D
Delta-latroinsectotoxin-XP_015914373.1	*P. tepidariorum*	/	L	T	P	L	H	I	A	T	R	N	G	F	D	K	T	V	L	I	L	M	A	N	E	N	L	Q	I	N	A	K	D	K	S	G	F	T	P	L	H	Y	V	I	Q	G	G	A	K	V	L	E
Delta-latroinsectotoxin-Sno1a	*S. nobilis*	This study	L	S	P	L	H	L	S	T	R	Y	G	F	L	E	T	V	W	R	L	L	N	S	T	D	I	K	V	N	Q	V	E	N	G	G	Y	T	P	L	H	T	A	I	S	G	G	P	H	V	V	K
Delta-latroinsectotoxin-Sno1b	*S. nobilis*	This study	L	T	A	L	H	L	S	S	S	S	G	F	S	K	T	V	K	T	L	L	K	S	P	N	I	N	V	N	Q	K	A	D	E	G	F	T	P	L	H	T	A	V	M	G	G	Y	Q	V	V	D
Delta-latroinsectotoxin-Sno1c	*S. nobilis*	This study	L	T	A	L	H	L	S	S	S	S	G	F	S	K	T	V	K	T	L	L	K	S	P	N	I	N	V	N	Q	K	A	D	E	G	F	T	P	L	H	T	A	V	M	G	G	Y	Q	V	V	D
Delta-latroinsectotoxin-Sno1d	*S. nobilis*	This study	M	S	A	L	H	T	S	A	E	Y	G	F	A	N	V	V	K	N	L	L	K	S	T	A	I	S	V	N	Q	V	A	Y	F	G	Y	T	S	L	H	A	A	V	V	G	G	I	Q	V	V	R
Delta-latroinsectotoxin-Sno1e	*S. nobilis*	This study	L	T	A	L	H	L	A	A	R	N	G	F	P	K	T	V	E	N	L	L	K	S	P	N	I	I	I	N	E	K	S	D	S	G	Y	T	A	L	H	A	A	I	D	G	G	F	R	V	L	E

IDENTIFICATION	SPECIES	REF	601	602	603	604	605	606	607	608	609	610	611	612	613	614	615	616	617	618	619	620	621	622	623	624	625	626	627	628	629	630	631	632	633	634	635	636	637	638	639	640	641	642	643	644	645	646	647	648	649	650
Delta-latroinsectotoxin-Lt1a Q25338	*L. tredecimguttatus*	[1]	A	L	L	N	H	P	D	I	D	K	N	A	Q	S	T	S	G	L	T	P	F	H	L	A	I	I	N	E	S	Q	E	V	A	E	S	L	V	E	S	N	A	-	D	L	N	I	Q	D	V	N
Delta-latroinsectotoxin-XP_015914373.1	*P. tepidariorum*	/	A	L	L	S	S	K	E	T	D	V	N	T	K	S	E	S	G	L	T	P	F	H	L	A	I	M	N	S	D	Q	E	V	A	E	V	L	E	D	S	Q	A	L	D	V	N	A	G	D	N	N
Delta-latroinsectotoxin-Sno1a	*S. nobilis*	This study	A	L	L	E	H	N	K	I	N	I	T	A	K	S	K	T	G	L	T	P	F	H	L	A	V	M	K	E	N	M	R	V	V	R	L	F	E	M	H	Q	D	-	D	L	N	I	A	D	N	N
Delta-latroinsectotoxin-Sno1b	*S. nobilis*	This study	A	M	L	Q	N	K	D	I	D	K	N	A	Q	S	D	T	G	L	T	P	F	H	L	A	I	I	N	E	N	K	E	V	A	E	S	L	V	E	Y	N	A	-	D	V	N	A	Q	D	F	N
Delta-latroinsectotoxin-Sno1c	*S. nobilis*	This study	A	M	L	Q	N	K	D	I	D	K	N	A	Q	S	D	T	G	L	T	P	F	H	L	A	I	I	N	E	N	K	E	V	A	E	S	L	V	E	Y	N	A	-	D	V	N	A	Q	D	F	N
Delta-latroinsectotoxin-Sno1d	*S. nobilis*	This study	P	L	L	E	H	P	D	I	D	V	N	A	K	S	E	N	G	L	T	P	L	H	L	A	V	I	K	N	N	L	T	I	T	K	A	L	I	G	G	E	A	-	D	I	N	S	E	D	I	D
Delta-latroinsectotoxin-Sno1e	*S. nobilis*	This study	V	L	M	K	K	E	D	L	D	L	N	A	K	S	D	D	G	L	T	P	F	H	L	A	V	M	N	G	D	I	E	V	A	Q	L	L	V	K	S	Q	A	-	D	L	N	A	R	D	I	N

IDENTIFICATION	SPECIES	REF	651	652	653	654	655	656	657	658	659	660	661	662	663	664	665	666	667	668	669	670	671	672	673	674	675	676	677	678	679	680	681	682	683	684	685	686	687	688	689	690	691	692	693	694	695	696	697	698	699	700
Delta-latroinsectotoxin-Lt1a Q25338	*L. tredecimguttatus*	[1]	H	M	A	P	I	H	F	A	A	S	M	G	S	I	K	M	L	R	Y	L	I	S	I	K	D	K	V	S	I	N	S	V	T	E	N	N	N	W	T	P	L	H	F	A	I	Y	F	K	K	E
Delta-latroinsectotoxin-XP_015914373.1	*P. tepidariorum*	/	G	L	T	A	L	H	F	A	C	M	A	E	N	T	K	M	I	E	F	L	V	S	R	-	K	G	I	D	V	N	A	V	T	Q	D	K	K	W	S	P	L	H	L	S	I	Y	F	R	K	E
Delta-latroinsectotoxin-Sno1a	*S. nobilis*	This study	G	V	T	A	L	H	Y	A	A	M	N	G	S	K	D	M	V	K	Y	L	L	S	L	A	D	R	I	N	V	N	A	V	T	L	N	R	R	W	T	P	L	H	F	A	I	Y	F	K	R	E
Delta-latroinsectotoxin-Sno1b	*S. nobilis*	This study	Y	M	A	P	I	H	F	A	V	T	S	G	S	T	N	M	L	K	Y	L	L	S	I	K	D	K	V	N	I	N	S	V	T	K	N	N	N	W	S	P	L	H	F	A	I	Y	F	K	Q	E
Delta-latroinsectotoxin-Sno1c	*S. nobilis*	This study	Y	M	A	P	I	H	F	A	V	T	S	G	S	T	N	M	L	K	Y	L	L	S	I	K	D	K	V	N	I	N	S	V	T	K	N	N	N	W	S	P	L	H	F	A	I	Y	F	K	Q	E
Delta-latroinsectotoxin-Sno1d	*S. nobilis*	This study	N	M	T	P	L	H	Y	A	S	I	N	G	T	K	Q	M	I	Q	Y	L	L	S	Q	N	-	-	G	N	G	N	A	V	T	L	E	N	N	F	T	P	L	Y	V	S	I	F	F	K	N	R
Delta-latroinsectotoxin-Sno1e	*S. nobilis*	This study	G	M	T	A	M	H	L	V	A	M	D	G	S	L	E	M	L	K	Y	L	L	S	L	K	K	K	I	D	I	N	S	V	V	L	D	S	N	W	T	P	L	H	F	A	I	Q	F	K	K	E

IDENTIFICATION	SPECIES	REF	701	702	703	704	705	706	707	708	709	710	711	712	713	714	715	716	717	718	719	720	721	722	723	724	725	726	727	728	729	730	731	732	733	734	735	736	737	738	739	740	741	742	743	744	745	746	747	748	749	750
Delta-latroinsectotoxin-Lt1a Q25338	*L. tredecimguttatus*	[1]	D	A	A	K	E	L	L	K	Q	D	D	I	N	L	T	I	V	A	D	G	N	L	T	V	L	H	L	A	V	S	T	G	Q	I	N	I	I	K	E	L	L	K	R	G	S	N	I	E	-	-
Delta-latroinsectotoxin-XP_015914373.1	*P. tepidariorum*	/	T	S	A	S	E	I	M	K	D	A	R	T	D	V	N	I	F	A	S	E	N	I	T	P	L	H	I	S	V	A	T	G	Q	L	N	I	I	T	G	L	L	D	R	R	A	N	I	E	-	-
Delta-latroinsectotoxin-Sno1a	*S. nobilis*	This study	E	A	Y	S	L	L	L	E	A	A	N	I	N	I	E	L	V	A	I	G	G	I	S	P	L	H	L	A	V	S	R	G	E	I	S	A	V	R	K	L	L	E	I	G	S	K	L	E	T	I
Delta-latroinsectotoxin-Sno1b	*S. nobilis*	This study	A	A	A	L	E	L	L	K	Q	E	D	T	D	V	T	I	V	T	N	D	N	L	T	V	L	H	L	A	V	S	K	G	Q	I	N	I	V	K	E	L	L	K	R	G	S	N	I	E	-	-
Delta-latroinsectotoxin-Sno1c	*S. nobilis*	This study	A	A	A	L	E	L	L	K	Q	E	D	T	D	V	T	I	V	T	N	D	N	L	T	V	L	H	L	A	V	S	K	G	Q	L	S	I	V	K	E	L	L	N	R	G	C	N	L	E	-	-
Delta-latroinsectotoxin-Sno1d	*S. nobilis*	This study	D	V	A	L	T	L	L	E	Y	T	T	I	N	H	H	L	P	A	I	K	N	V	T	P	F	H	L	A	V	A	T	G	Q	I	D	I	V	K	K	L	L	S	K	G	I	D	L	E	-	-
Delta-latroinsectotoxin-Sno1e	*S. nobilis*	This study	N	A	A	L	E	L	L	K	D	E	Y	V	D	L	S	I	A	A	H	D	N	I	T	V	L	H	L	A	S	A	T	G	Q	A	N	I	V	K	E	L	L	N	R	G	I	S	L	E	-	-

IDENTIFICATION	SPECIES	REF	751	752	753	754	755	756	757	758	759	760	761	762	763	764	765	766	767	768	769	770	771	772	773	774	775	776	777	778	779	780	781	782	783	784	785	786	787	788	789	790	791	792	793	794	795	796	797	798	799	800
Delta-latroinsectotoxin-Lt1a Q25338	*L. tredecimguttatus*	[1]	-	-	-	-	-	-	-	-	-	E	K	T	G	E	G	Y	T	S	L	H	I	A	A	M	R	K	E	P	E	I	A	V	V	L	I	E	N	G	A	D	I	E	A	R	S	A	D	N	L	T
Delta-latroinsectotoxin-XP_015914373.1	*P. tepidariorum*	/	-	-	-	-	-	-	-	-	-	A	K	T	I	E	G	F	T	P	L	H	L	A	A	L	R	P	E	P	T	I	S	S	L	L	L	R	N	G	A	N	I	N	S	K	S	N	D	G	S	T
Delta-latroinsectotoxin-Sno1a	*S. nobilis*	This study	I	K	E	G	Y	T	Y	L	E	Q	P	T	N	K	G	Y	T	P	L	H	L	A	V	L	R	N	E	P	E	I	V	S	L	L	I	Q	N	N	A	N	I	E	E	T	S	G	E	K	E	T
Delta-latroinsectotoxin-Sno1b	*S. nobilis*	This study	-	-	-	-	-	-	-	-	-	D	R	T	S	E	G	Y	T	P	L	H	L	G	A	M	R	K	E	P	E	M	A	L	L	L	I	D	K	G	A	N	I	E	A	R	A	A	N	N	T	T
Delta-latroinsectotoxin-Sno1c	*S. nobilis*	This study	-	-	-	-	-	-	-	-	-	D	K	T	S	E	G	Y	T	P	L	H	L	G	A	M	R	K	E	P	E	M	A	L	L	L	I	D	N	G	A	N	I	E	A	R	A	A	N	N	T	T
Delta-latroinsectotoxin-Sno1d	*S. nobilis*	This study	-	-	-	-	-	-	-	-	-	E	K	T	E	E	G	Y	T	A	L	H	L	A	S	F	R	D	E	T	K	L	S	S	L	L	I	S	K	G	A	D	L	D	S	R	T	S	N	N	Y	T
Delta-latroinsectotoxin-Sno1e	*S. nobilis*	This study	-	-	-	-	-	-	-	-	-	E	K	T	D	E	G	Y	T	A	L	H	V	G	A	M	R	K	E	S	E	I	V	K	L	L	I	E	K	G	A	N	I	E	A	R	S	A	D	N	L	T

Supplementary dataset 2 – Table 4c- Delta-Latroinsectotoxins- Sequences from position 801 to 1176: Sequence alignments of the closest two delta-latroinsectotoxins already published and the five most abundant found in the venom of *Steatoda nobilis*. Yellow windows indicate positions where the identity and/or the physico-chemical properties of the aminoacids is maintained at least for 4 possibilities over 7, bold positions are the ones where the positions are maintained 2 or 3 times (over 7). Cysteines are highlighted in red.

IDENTIFICATION	SPECIES	REF	801	802	803	804	805	806	807	808	809	810	811	812	813	814	815	816	817	818	819	820	821	822	823	824	825	826	827	828	829	830	831	832	833	834	835	836	837	838	839	840	841	842	843	844	845	846	847	848	849	850
Delta-latroinsectotoxin-Lt1a Q25338	*L. tredecimguttatus*	[1]	P	L	H	S	A	A	K	I	G	R	K	S	T	V	L	**Y**	L	L	E	K	G	A	D	I	G	A	K	T	A	D	G	S	T	A	L	H	L	A	V	S	G	R	K	M	K	T	V	E	T	L
Delta-latroinsectotoxin-XP_015914373.1	*P. tepidariorum*	/	P	L	H	L	A	T	-	-	-	-	-	-	-	-	-	-	-	-	-	-	-	-	-	-	-	-	-	-	-	-	-	-	-	-	-	-	-	-	-	K	A	E	R	I	E	T	L	T	N	L
Delta-latroinsectotoxin-Sno1a	*S. nobilis*	*This study*	P	L	H	L	A	A	S	-	-	-	-	-	-	-	-	-	-	-	-	-	-	-	-	-	-	-	-	-	-	-	-	-	-	-	-	-	-	-	-	-	R	G	A	L	D	I	V	K	M	L
Delta-latroinsectotoxin-Sno1b	*S. nobilis*	*This study*	P	L	H	L	A	A	K	A	G	R	K	T	T	L	L	**N**	L	L	E	K	G	A	D	I	**E**	A	K	T	**E**	D	G	S	T	A	L	H	I	A	A	S	G	R	K	I	**N**	T	V	K	A	L
Delta-latroinsectotoxin-Sno1c	*S. nobilis*	*This study*	P	L	H	L	A	A	K	A	G	R	K	T	T	L	L	**N**	L	L	E	K	G	A	D	I	**E**	A	K	T	**E**	D	G	S	T	A	L	H	I	A	A	S	G	R	K	I	**N**	T	V	K	A	L
Delta-latroinsectotoxin-Sno1d	*S. nobilis*	*This study*	P	L	H	F	A	T	R	H	G	N	I	N	T	L	L	**Y**	L	L	N	M	G	A	D	I	**S**	A	K	T	S	D	G	S	T	A	L	H	I	A	T	**E**	F	K	L	L	D	A	V	R	A	L
Delta-latroinsectotoxin-Sno1e	*S. nobilis*	*This study*	P	L	H	L	A	A	K	T	G	R	M	S	T	L	L	E	L	I	D	K	G	A	D	M	**S**	S	K	T	P	D	G	S	T	P	L	H	L	A	V	**D**	N	Q	Q	L	D	T	V	K	A	L

IDENTIFICATION	SPECIES	REF	851	852	853	854	855	856	857	858	859	860	861	862	863	864	865	866	867	868	869	870	871	872	873	874	875	876	877	878	879	880	881	882	883	884	885	886	887	888	889	890	891	892	893	894	895	896	897	898	899	900
Delta-latroinsectotoxin-Lt1a Q25338	*L. tredecimguttatus*	[1]	L	**N**	K	G	A	N	L	K	E	Y	D	**N**	N	**K**	Y	L	P	I	H	K	A	I	T	V	L	**Y**	L	L	E	K	G	A	D	I	G	A	K	T	A	D	G	S	T	A	L	H	L	A	V	S
Delta-latroinsectotoxin-XP_015914373.1	*P. tepidariorum*	/	L	**N**	K	G	A	**D**	S	R	S	V	D	H	N	**N**	L	L	P	I	Q	I	A	I	-	-	-	-	-	-	-	-	-	-	-	-	-	-	-	-	-	-	-	-	-	-	-	-	-	-	-	K
Delta-latroinsectotoxin-Sno1a	*S. nobilis*	*This study*	L	**A**	E	K	A	T	L	L	K	Y	D	I	H	**G**	Y	T	P	A	N	Y	A	A	-	-	-	-	-	-	-	-	-	-	-	-	-	-	-	-	-	-	-	-	-	-	-	-	-	-	-	-
Delta-latroinsectotoxin-Sno1b	*S. nobilis*	*This study*	L	**S**	K	G	A	N	L	K	E	F	D	**N**	H	**K**	Y	L	P	**F**	H	K	A	I	T	L	L	**N**	L	L	E	K	G	A	D	I	**E**	A	K	T	**E**	D	G	S	T	A	L	H	I	A	A	S
Delta-latroinsectotoxin-Sno1c	*S. nobilis*	*This study*	L	**S**	K	G	A	N	L	K	E	F	D	**N**	H	**K**	Y	L	P	**F**	H	K	A	I	T	L	L	**N**	L	L	E	K	G	A	D	I	**E**	A	K	T	**E**	D	G	S	T	A	L	H	I	A	A	S
Delta-latroinsectotoxin-Sno1d	*S. nobilis*	*This study*	L	**S**	K	G	A	N	**A**	K	E	Y	D	**D**	N	**G**	Y	L	P	I	H	K	P	I	T	L	L	**Y**	L	L	N	M	G	A	D	I	**S**	A	K	T	S	D	G	S	T	A	L	H	I	A	T	E
Delta-latroinsectotoxin-Sno1e	*S. nobilis*	*This study*	L	**G**	K	G	A	**D**	**V**	K	A	T	D	**D**	N	**N**	Y	L	P	I	H	K	A	M	T	L	L	E	L	I	D	K	G	A	D	M	**S**	S	K	T	P	D	G	S	T	P	L	H	L	A	V	D

IDENTIFICATION	SPECIES	REF	901	902	903	904	905	906	907	908	909	910	911	912	913	914	915	916	917	918	919	920	921	922	923	924	925	926	927	928	929	930	931	932	933	934	935	936	937	938	939	940	941	942	943	944	945	946	947	948	949	950
Delta-latroinsectotoxin-Lt1a Q25338	*L. tredecimguttatus*	[1]	G	R	K	M	K	T	V	E	T	L	L	**N**	K	G	A	N	L	K	E	Y	D	**N**	N	K	Y	L	P	I	H	K	A	I	I	N	**D**	D	L	D	M	V	R	L	F	L	E	K	D	**P**	S	L
Delta-latroinsectotoxin-XP_015914373.1	*P. tepidariorum*	/	A	E	R	I	E	T	L	T	N	L	L	**N**	K	G	A	D	S	R	S	V	D	H	N	**N**	L	L	P	I	Q	I	A	I	Q	N	Y	N	T	D	**I**	I	R	K	F	M	N	H	D	**R**	I	I
Delta-latroinsectotoxin-Sno1a	*S. nobilis*	*This study*	R	G	**A**	L	D	I	V	K	M	L	L	**A**	E	K	A	T	L	L	K	Y	D	I	**H**	G	Y	T	P	A	N	Y	A	A	K	N	**N**	D	V	E	M	**M**	K	L	F	V	Q	Y	D	**P**	K	L
Delta-latroinsectotoxin-Sno1b	*S. nobilis*	*This study*	G	R	K	I	**N**	T	V	K	A	L	L	**S**	K	G	A	N	L	K	E	F	D	**N**	**H**	K	Y	L	P	**F**	H	K	A	I	I	N	**E**	D	**K**	D	M	**M**	R	L	F	I	D	Q	D	**P**	S	L
Delta-latroinsectotoxin-Sno1c	*S. nobilis*	*This study*	G	R	K	I	**N**	T	V	K	A	L	L	**S**	K	G	A	N	L	K	E	F	D	**N**	**H**	K	Y	L	P	**F**	H	K	A	I	I	N	**E**	D	**K**	D	M	**M**	R	L	F	I	E	K	D	A	S	L
Delta-latroinsectotoxin-Sno1d	*S. nobilis*	*This study*	F	K	**L**	L	D	A	V	R	A	L	L	**S**	K	G	A	N	A	K	E	Y	D	**D**	N	G	Y	L	P	I	H	K	P	I	L	**D**	N	E	I	D	**I**	V	K	A	V	I	E	H	D	**H**	N	L
Delta-latroinsectotoxin-Sno1e	*S. nobilis*	*This study*	N	Q	Q	L	D	T	V	K	A	L	L	**G**	K	G	A	D	V	K	A	T	D	**D**	N	**N**	Y	L	P	I	H	K	A	M	L	**E**	N	D	A	E	**I**	V	R	M	L	V	K	H	D	**H**	S	L

IDENTIFICATION	SPECIES	REF	951	952	953	954	955	956	957	958	959	960	961	962	963	964	965	966	967	968	969	970	971	972	973	974	975	976	977	978	979	980	981	982	983	984	985	986	987	988	989	990	991	992	993	994	995	996	997	998	999	1000
Delta-latroinsectotoxin-Lt1a Q25338	*L. tredecimguttatus*	[1]	K	D	D	E	T	E	E	G	R	T	-	-	-	-	S	I	M	L	I	**V**	**Q**	K	L	L	L	E	L	Y	N	Y	F	I	N	**N**	Y	A	E	T	**L**	**D**	-	-	-	**E**	E	**A**	L	F	N	R
Delta-latroinsectotoxin-XP_015914373.1	*P. tepidariorum*	/	**V**	N	P	**K**	T	E	E	**F**	L	I	H	N	N	K	I	L	S	F	T	F	S	T	L	V	**R**	**Y**	L	Y	H	Y	Y	L	N	K	D	S	P	E	**Y**	Q	E	F	R	**D**	M	T	P	Y	**Q**	I
Delta-latroinsectotoxin-Sno1a	*S. nobilis*	*This study*	M	T	S	**D**	N	K	G	**F**	N	E	-	-	-	-	L	F	**D**	I	V	**M**	G	R	M	V	Q	D	S	F	K	Y	**L**	A	K	N	**D**	D	D	S	**V**	**E**	-	-	-	**I**	P	**D**	I	I	**K**	**R**
Delta-latroinsectotoxin-Sno1b	*S. nobilis*	*This study*	**S**	K	D	**D**	T	E	E	G	R	T	-	-	-	-	S	A	K	L	I	**M**	**Q**	K	I	V	V	**F**	L	Y	N	H	F	I	N	**T**	Y	D	E	T	**E**	**S**	-	-	-	**L**	E	**K**	I	H	**E**	H
Delta-latroinsectotoxin-Sno1c	*S. nobilis*	*This study*	**T**	N	D	N	T	E	E	G	R	T	-	-	-	-	S	**T**	I	L	I	**V**	**Q**	K	I	V	V	S	L	Y	N	H	F	I	N	**T**	Y	D	E	T	**E**	**S**	-	-	-	**L**	E	**K**	I	H	**E**	H
Delta-latroinsectotoxin-Sno1d	*S. nobilis*	*This study*	**L**	**D**	E	**K**	T	N	E	Y	K	S	-	-	-	-	I	**T**	**E**	S	I	R	R	K	L	L	I	D	I	Y	N	Y	**L**	K	D	S	N	Y	P	N	K	**T**	-	-	-	H	D	**A**	M	Y	**Q**	Y
Delta-latroinsectotoxin-Sno1e	*S. nobilis*	*This study*	**I**	**D**	D	S	V	E	N	A	Q	S	-	-	-	-	S	L	Q	L	I	I	D	I	L	M	**K**	D	I	Y	N	Y	Y	I	N	**N**	Y	D	E	T	Q	G	-	-	-	N	E	**E**	I	F	**K**	R

IDENTIFICATION	SPECIES	REF	1001	1002	1003	1004	1005	1006	1007	1008	1009	1010	1011	1012	1013	1014	1015	1016	1017	1018	1019	1020	1021	1022	1023	1024	1025	1026	1027	1028	1029	1030	1031	1032	1033	1034	1035	1036	1037	1038	1039	1040	1041	1042	1043	1044	1045	1046	1047	1048	1049	1050
Delta-latroinsectotoxin-Lt1a Q25338	*L. tredecimguttatus*	[1]	**L**	D	E	Q	G	K	L	E	L	A	Y	I	F	H	**N**	**K**	**E**	G	**D**	A	K	E	-	-	-	-	A	**V**	K	**P**	**T**	**I**	L	-	**V**	T	I	**K**	L	**M**	**E**	Y	C	L	K	**K**	**L**	R	**E**	**E**
Delta-latroinsectotoxin-XP_015914373.1	*P. tepidariorum*	/	F	E	K	Q	G	K	I	E	**M**	L	K	V	L	M	**E**	**G**	**S**	**E**	**E**	V	**-**	-	-	-	-	-	**K**	**I**	**L**	**P**	I	**I**	V	N	**G**	**I**	D	**N**	I	A	K	H	C	V	**T**	K	T	**T**	N	Q
Delta-latroinsectotoxin-Sno1a	*S. nobilis*	*This study*	**L**	E	N	E	G	I	L	D	**M**	Y	Y	V	L	G	**E**	**G**	**S**	R	**D**	L	S	S	N	D	L	K	**K**	Y	**A**	Y	G	**L**	M	T	**V**	**F**	V	**K**	I	I	R	K	C	F	**S**	**Y**	**L**	**S**	**K**	**A**
Delta-latroinsectotoxin-Sno1b	*S. nobilis*	*This study*	F	**H**	E	Q	G	K	L	E	L	A	Y	**T**	F	**T**	**N**	**K**	**P**	**N**	**S**	K	**E**	-	-	-	-	-	**E**	F	K	**T**	A	**F**	L	-	**F**	**M**	A	**A**	A	V	K	H	C	L	**E**	**E**	**K**	R	**L**	**A**
Delta-latroinsectotoxin-Sno1c	*S. nobilis*	*This study*	F	**H**	E	Q	G	K	L	E	L	A	Y	**T**	F	**T**	**N**	**K**	**P**	**N**	**S**	K	**E**	-	-	-	-	-	**E**	F	K	**T**	A	**F**	L	-	**F**	**M**	A	**A**	A	V	K	H	C	L	**E**	**E**	**K**	R	**L**	**A**
Delta-latroinsectotoxin-Sno1d	*S. nobilis*	*This study*	M	E	E	N	N	K	F	R	I	T	L	F	F	I	K	D	**E**	**D**	P	K	**E**	-	-	-	-	-	S	**L**	K	-	V	S	L	Q	M	**Y**	K	**G**	L	**M**	**E**	K	C	I	N	**G**	M	D	P	E
Delta-latroinsectotoxin-Sno1e	*S. nobilis*	*This study*	F	S	E	E	G	K	L	D	V	A	F	I	F	N	A	S	**E**	**D**	**S**	K	**-**	K	-	-	-	-	M	F	K	**S**	**T**	**F**	F	-	**F**	**M**	L	**N**	A	V	K	L	C	L	**E**	T	**K**	R	**K**	**E**

IDENTIFICATION	SPECIES	REF	1051	1052	1053	1054	1055	1056	1057	1058	1059	1060	1061	1062	1063	1064	1065	1066	1067	1068	1069	1070	1071	1072	1073	1074	1075	1076	1077	1078	1079	1080	1081	1082	1083	1084	1085	1086	1087	1088	1089	1090	1091	1092	1093	1094	1095	1096	1097	1098	1099	1100
Delta-latroinsectotoxin-Lt1a Q25338	*L. tredecimguttatus*	[1]	S	G	A	-	-	P	-	-	-	-	-	-	-	-	-	-	-	-	-	-	-	-	-	-	-	-	-	-	-	-	-	-	-	-	-	-	-	-	-	-	-	-	-	-	-	-	-	-	-	-
Delta-latroinsectotoxin-XP_015914373.1	*P. tepidariorum*	/	D	D	S	K	D	T	V	D	P	C	V	S	S	R	H	V	R	R	H	I	L	S	D	L	K	N	S	V	K	K	L	L	K	S	N	V	Y	N	F	V	L	S	E	A	S	F	N	R	M	N
Delta-latroinsectotoxin-Sno1a	*S. nobilis*	*This study*	S	I	**E**	-	-	D	L	S	**S**	S	S	-	-	-	-	-	-	-	-	-	-	-	-	-	-	-	-	-	-	-	-	-	-	-	-	-	-	-	-	-	-	-	-	-	-	-	-	-	-	-
Delta-latroinsectotoxin-Sno1b	*S. nobilis*	*This study*	S	G	**S**	-	-	S	G	D	-	S	T	-	-	-	-	-	-	-	-	-	-	-	-	-	-	-	-	-	-	-	-	-	-	-	-	-	-	-	-	-	-	-	-	-	-	-	-	-	-	-
Delta-latroinsectotoxin-Sno1c	*S. nobilis*	*This study*	S	G	**S**	-	-	S	G	D	-	S	T	-	-	-	-	-	-	-	-	-	-	-	-	-	-	-	-	-	-	-	-	-	-	-	-	-	-	-	-	-	-	-	-	-	-	-	-	-	-	-
Delta-latroinsectotoxin-Sno1d	*S. nobilis*	*This study*	T	S	**E**	I	D	D	-	-	-	-	-	-	-	-	-	-	-	-	-	-	-	-	-	-	-	-	-	-	-	-	-	-	-	-	-	-	-	-	-	-	-	-	-	-	-	-	-	-	-	-
Delta-latroinsectotoxin-Sno1e	*S. nobilis*	*This study*	S	I	**E**	-	-	T	V	D	**S**	S	S	-	-	-	-	-	-	-	-	-	-	-	-	-	-	-	-	-	-	-	-	-	-	-	-	-	-	-	-	-	-	-	-	-	-	-	-	-	-	-

IDENTIFICATION	SPECIES	REF	1101	1102	1103	1104	1105	1106	1107	1108	1109	1110	1111	1112	1113	1114	1115	1116	1117	1118	1119	1120	1121	1122	1123	1124	1125	1126	1127	1128	1129	1130	1131	1132	1133	1134	1135	1136	1137	1138	1139	1140	1141	1142	1143	1144	1145	1146	1147	1148	1149	1150
Delta-latroinsectotoxin-Lt1a Q25338	*L. tredecimguttatus*	[1]	-	-	-	-	-	-	-	-	-	-	-	-	-	E	G	**S**	F	**D**	S	P	**S**	-	-	-	-	-	-	-	-	-	-	**S**	**K**	**Q**	C	I	-	-	-	**S**	**T**	F	**S**	E	D	E	M	F	-	-
Delta-latroinsectotoxin-XP_015914373.1	*P. tepidariorum*	/	G	V	H	Q	N	K	S	E	Y	R	N	K	N	V	R	**I**	I	**Q**	S	P	D	S	V	**K**	**T**	S	**Y**	Q	M	K	N	I	T	**Q**	T	L	-	-	-	**S**	E	Y	**R**	N	N	N	**K**	Q	-	-
Delta-latroinsectotoxin-Sno1a	*S. nobilis*	*This study*	-	-	-	-	-	-	-	-	-	-	-	-	-	-	N	**I**	L	**K**	S	E	**N**	-	-	-	-	-	-	-	-	-	-	W	E	K	C	A	K	I	K	**Y**	**T**	F	**K**	**R**	**R**	S	L	**K**	-	-
Delta-latroinsectotoxin-Sno1b	*S. nobilis*	*This study*	-	-	-	-	-	-	-	-	-	-	-	-	-	-	-	-	-	-	-	-	-	-	-	**R**	**S**	V	**P**	-	-	-	-	-	-	-	-	A	S	S	L	Q	**S**	P	**T**	-	-	-	-	-	-	-
Delta-latroinsectotoxin-Sno1c	*S. nobilis*	*This study*	-	-	-	-	-	-	-	-	-	-	-	-	-	A	S	**S**	L	**Q**	S	P	**T**	-	-	-	-	-	-	-	-	-	-	**S**	G	**Q**	C	L	-	-	-	-	-	-	-	-	-	-	-	-	-	-
Delta-latroinsectotoxin-Sno1d	*S. nobilis*	*This study*	-	-	-	-	-	-	-	-	-	-	-	-	-	T	L	**I**	F	**D**	S	P	V	-	-	-	-	-	-	-	-	-	-	L	**K**	M	C	Y	-	-	-	**Y**	R	L	P	**K**	**K**	**L**	**R**	P	L	I
Delta-latroinsectotoxin-Sno1e	*S. nobilis*	*This study*	-	-	-	-	-	-	-	-	-	-	-	-	-	P	H	**S**	L	**K**	S	I	**N**	-	-	-	-	-	-	-	-	-	-	**S**	**K**	E	C	I	-	-	-	N	**T**	F	**S**	G	Y	**L**	**H**	**R**	-	-

IDENTIFICATION	SPECIES	REF	1151	1152	1153	1154	1155	1156	1157	1158	1159	1160	1161	1162	1163	1164	1165	1166	1167	1168	1169	1170	1171	1172	1173	1174	1175	1176
Delta-latroinsectotoxin-Lt1a Q25338	*L. tredecimguttatus*	[1]	-	-	-	-	-	-	-	-	-	-	R	**R**	T	L	P	-	-	-	-	-	-	-	-	-	-	E
Delta-latroinsectotoxin-XP_015914373.1	*P. tepidariorum*	/	-	-	A	**V**	Q	**S**	-	T	D	S	R	**K**	**A**	F	N	W	M	K	**S**	I	T	Q	N	H	**S**	E
Delta-latroinsectotoxin-Sno1a	*S. nobilis*	*This study*	-	-	-	-	-	-	-	-	-	-	-	-	-	-	-	-	-	-	-	-	-	A	D	V	E	E
Delta-latroinsectotoxin-Sno1b	*S. nobilis*	*This study*	-	-	-	S	G	Q	C	L	-	-	-	T	**E**	F	I	E	S	I	L	**S**	-	-	-	-	-	E
Delta-latroinsectotoxin-Sno1c	*S. nobilis*	*This study*	-	-	-	-	-	-	-	-	-	-	-	-	-	-	-	-	-	-	-	-	-	-	-	-	-	-
Delta-latroinsectotoxin-Sno1d	*S. nobilis*	*This study*	I	P	N	**V**	R	**S**	T	A	S	F	R	R	**G**	I	N	F	E	Q	**S**	**T**	-	-	-	-	**S**	E
Delta-latroinsectotoxin-Sno1e	*S. nobilis*	*This study*	-	-	-	-	-	-	-	-	-	-	R	S	**D**	F	S	-	-	-	-	-	-	-	-	-	-	D
